# Impact of Soil Drought on Yield and Leaf Sugar Content in Wheat: Genotypic and Phenotypic Relationships Compared Using a Doubled Haploid Population

**DOI:** 10.3390/ijms26167833

**Published:** 2025-08-13

**Authors:** Magdalena Grela, Steve Quarrie, Katarzyna Cyganek, Jan Bocianowski, Małgorzata Karbarz, Mirosław Tyrka, Dimah Habash, Michał Dziurka, Edyta Kowalczyk, Wojciech Szarski, Ilona Mieczysława Czyczyło-Mysza

**Affiliations:** 1 The Franciszek Gorski Institute of Plant Physiology, Polish Academy of Sciences, Niezapominajek 21, 30-239 Kraków, Poland; 2 Biology Faculty, Belgrade University, Studentski trg 16, 11000 Belgrade, Serbia; steve.quarrie@ncl.ac.uk; 3 Department of Mathematical and Statistical Methods, Poznań University of Life Sciences, Wojska Polskiego 28, 60-637 Poznań, Poland; jan.bocianowski@up.poznan.pl; 4 Faculty of Biology and Nature Protection, University of Rzeszow, Zelwerowicza Street 4, 35-601 Rzeszów, Poland; mkarbarz@ur.edu.pl; 5 Department of Biotechnology and Bioinformatics, Faculty of Chemistry, Rzeszów University of Technology, Al. Powstancow Warszawy 12, 35-959 Rzeszów, Poland; mtyrka@prz.edu.pl; 6 Plant Science Department, Rothamsted Research, West Common, Harpenden AL5 2JQ, UK; dimah.habash@securewheat.com

**Keywords:** *QTL*, drought, agronomic traits—yield, biomass, TGW, carbohydrates, DH population, *Triticum aestivum*

## Abstract

Improving yield stability under water-limited conditions is a key objective of wheat breeding programmes. One trait of particular interest is carbohydrate accumulation and remobilisation. This study assessed the genetic basis of aspects of yield and flag leaf sugar contents under drought and well-watered conditions using *QTL* mapping in a population of 90 doubled haploid lines derived from the cross Chinese Spring × SQ1. As well as soluble sugar content, glucose, fructose, sucrose, and maltose, the traits grain yield (Yld), biomass (Bio), and thousand grain weight (TGW) were also analysed. Analysis of variance showed that genotype, environment and their interactions significantly influenced all the traits studied, with environmental effects explaining up to 74.4% of the total variation. *QTL* analysis identified 40 *QTLs* for Yld, TGW, and Bio as well as 53 *QTLs* for soluble carbohydrates, accounting for up to 40% of phenotypic variation. *QTLs* coincident for more than one trait were identified on 21 chromosome regions, associated with carbohydrate metabolism and yield performance under drought, particularly on chromosomes 2D, 4A, 4B, 5B, 5D, 6B, and 7A. Candidate genes for several yield-related *QTLs* were identified. These results provide useful genetic markers for the development of more drought-resistant wheat cultivars.

## 1. Introduction

Common wheat (*Triticum aestivum*) is one of the most important species of cereal crops in the global food economy, and the efficiency of its production is closely correlated with soil water availability.

Drought can severely reduce crop yields by disrupting physiological and reproductive processes in plants. The reduction in yield under drought stress is primarily attributed to the inhibition of photosynthetic activity, limitations in nutrient acquisition, and impairments in reproductive development. Drought stress adversely affects both photosystems, PSI and PSII, resulting in substantial declines in crop productivity and quality. The impairment of photosynthetic efficiency primarily stems from compromised chlorophyll integrity, reduced CO_2_ availability due to stomatal closure, and oxidative damage to cellular structures. These detrimental effects are particularly pronounced in staple crops critical for global food security [1].

To cope with adverse environmental conditions, plants modulate their metabolic and physiological processes to enhance survival. In cereals, grain filling relies on carbon assimilated post-anthesis and directly translocated to the grain, as well as carbon remobilised from assimilates stored in vegetative tissues [2,3]. A diminished carbon supply under suboptimal conditions often results in reduced grain yield and may lead to an increased proportion of small, shrivelled grains, ultimately lowering crop value and diminishing economic returns for growers [4]. In many plants, including wheat and other winter cereals, stem-reserve carbohydrates are predominantly stored as water-soluble carbohydrates (WSCs), which mainly comprise fructosyl-oligosaccharides (fructans), along with sucrose and hexoses [4,5,6]. In wheat and its close relatives, such as barley and oat, the accumulation of water-soluble carbohydrates (WSCs) and their subsequent remobilisation into developing grains represent a key adaptive mechanism [7].

Under favourable conditions, assimilates derived from photosynthesis are directly allocated to the developing grain, whereas under suboptimal photosynthetic conditions, stored sugars serve as a crucial carbon source [2,8]. This suggests that the accumulation and remobilisation of WSCs in wheat play a vital role in stabilising grain yield under both optimal and stress conditions. WSCs primarily consist of glucose (Glu), fructose (Fru), sucrose (Suc), and fructans (Fruc), with fructans constituting the predominant fraction [9].

The accumulation of water-soluble carbohydrates is widely recognised as an adaptive mechanism that enables plants to cope with drought stress. These carbohydrates, along with other compatible solutes such as proline, function as osmolytes, helping to maintain cell turgor, preserve membrane integrity, and prevent protein denaturation [10,11]. This osmolyte build-up imposes a substantial carbohydrate demand at a time when carbon fixation is typically limited. Sucrose, glucose, and fructose play pivotal roles in plant metabolism at both the cellular and whole-plant levels. In addition to their function in abiotic stress responses, these sugars act as signalling molecules, regulating the expression of numerous metabolic genes through sugar-sensing pathways [12,13,14,15].

Furthermore, previous studies indicate that the remobilisation of WSCs to developing grains can contribute up to 20% under optimal conditions and up to 70% under water stress [16]. Consequently, WSCs play a critical role in sustaining grain growth under water-deficit conditions [17].

Molecular quantitative genetics provides a powerful framework for investigating complex quantitative traits by characterising continuous phenotypic distributions and estimating the number of contributing loci, the predominant gene action, and the extent of interactions between quantitative trait loci (*QTL*) and the environment [18].

Moreover, several candidate genes involved in the carbohydrate biosynthetic pathway co-localise with *QTLs* for soluble carbohydrates [19]. In barley subjected to drought conditions, additive *QTLs* for leaf WSCs were found to overlap with *QTLs* associated with plant water status and/or osmotic adjustment, forming distinct *QTL* clusters [7].

The remobilisation of stem WSCs is well known to contribute to grain yield in wheat. However, there is considerable genetic variation in the contribution of stem WSCs to grain yield under post-anthesis water deficit. Fructan 1-exohydrolase (1-FEH) is one of the major enzymes contributing to WSC remobilisation and the maintenance of grain yield under water-deficit conditions. 1-FEH has three isoforms (1-FEH w1, w2 and w3) that degrade β-(2-1) fructan linkages, thus contributing to fructan remobilisation to the grain [9].

To enhance drought resilience, it is imperative to understand the interplay between genetic control and environmental responses. The present study proposes an integrated approach that combines *QTL* mapping with phenotypic analysis using an additive main effects and multiplicative interaction (AMMI) model and phenotypic correlation analysis. The AMMI approach integrates analysis of variance (ANOVA) with principal component analysis (PCA), thus offering a robust framework for analysing genotype–environment interactions [20,21]. This combined approach provides novel insights into the selection of genotypes that consistently demonstrate superior performance under varying water availability.

Although the genetic analysis of WSCs in wheat stems and their remobilisation during grain filling has been well studied ([22] and references cited therein), very little information is currently available about the genetic control of WSCs in flag leaves during grain filling [23].

To fill this gap in our knowledge of the genetic control of WSCs in wheat flag leaves, our study investigated the impact of soil drought on grain yield and leaf sugar contents in wheat flag leaves and established the genetic and phenotypic relationships underlying this impact. Thus, we conducted a quantitative trait locus (*QTL*) analysis alongside a comprehensive statistical analysis in a doubled haploid wheat population.

## 2. Results

### 2.1. Phenotypic Variation

The three sources of variation (genotype, environment, and the GEI) were highly significant for all eight agronomic and sugar traits (Table 1). In the ANOVA, the sum of squares for the main environment effect ranged from 28.13% (for fructose) to 74.40% (for biomass) of the total trait variation, and this factor had the highest effect on all observed traits except glucose and fructose (Table 1). Differences among genotypes explained from 5.63% (for yield) to 21.19% (for fructose) of the total trait variation, while GEI explained from 9.75% (for biomass) to 42.14% (for fructose), and this factor had the highest effect on glucose and fructose (Table 1). Values for the first three principal components were also significant (at the 0.001 level) for all observed traits, together representing from 78.97% (for DUBOIS) to 89.72% (for fructose) of the entire effect (Table 1). The first principal component (IPCA_1_) accounted for from 31.84% (for TGW) to 53.71% (for fructose) of the variation caused by the interaction (Table 1). IPCA_2_ accounted for from 20.62% (for DUBOIS) to 26.87% (for TGW) of the variation caused by the interaction (Table 1). The data presented in Table 2 include means of the measured parameters over the course of the three-year study period, together with standard deviations (SD), minima (Min), and maxima (Max) for the doubled haploid lines (DHLs) and their parents. For many traits, transgressive segregation amongst DHLs was apparent, as ranges amongst DHLs were much greater than differences between the parent means (Table 2). Yield components for both parents (CS, SQ1) and the CSDH population were variable in both treatments (D, C). Drought adversely affected the production of the above-ground mass of plants. As shown in Table 2, higher contents of the ethanol-soluble sugars (Dubois) were observed in CS, which produces less ABA under drought, than SQ1. The CSDH population showed a wide range in the contents of individual sugars (from 0.251 to 174.7 μg mg^−1^ dry mass).

### 2.2. Phenotypic Analysis of Individual Traits

#### 2.2.1. Yield

Yield ranged from 0.107 g (for DH94 in 2013,D) to 4.437 g (for DH26 in 2010,C), with an average of 1.72 g (Table S1). DH26 had the highest average yield (2.731 g), while DH94 had the lowest (1.248 g). The average yield in individual environments ranged from 0.697 g (in 2013,D) to 3.052 g (in 2010,C) (Table S1). DHLs 8, 66, 18, and 24 were positively adapted to environment 2012,C. DH10 was adapted in environments with similar conditions to those in 2010,D and 2013,D, and not adapted in 2010,C (Figure S1). DH143 was adapted in environments with similar conditions to those in 2012,D, and not adapted in 2012,C. DHLs 26, 53, and 71 were positively adapted to environment 2010,C. Lines DH39 and DH19, with high average yields (1.902 g and 1.870 g, respectively) and ASVs of 0.078 and 0.190, respectively, had the best genotype selection indices (26 and 31, respectively).

#### 2.2.2. Biomass

Biomass ranged from 0.294 g (for DH62 in 2013,D) to 8.814 g (for DH26 in 2010,C), with an average of 3.54 g (Table S2). Line DH143 was positively adapted to environment 2012,D. DH10 and DH23 were adapted in environments with similar conditions to those in 2013,D, and not adapted in 2010,D (Figure S2). DH40, DH49, and DH60 were adapted in environments with similar conditions to those in 2010,D and not adapted in 2012,C. DH8 was positively adapted to environment 2012,C. Lines DH19 and DH69, with high average biomasses (4.136 g and 3.979 g, respectively) and ASVs of 0.163 and 0.059, respectively, had the best genotype selection indices (18 and 20, respectively).

#### 2.2.3. TGW

TGW ranged from 11.67 g (for DH49 in 2012,D) to 51.67 g (for DH144 in 2012,C), with an average of 27.84 g (Table S3). Line DH1 and SQ1 were positively adapted to environment 2010,C. DH37, DH49, and DH60 were adapted in environments with similar conditions to those in 2010,D, and not adapted in 2012,D (Figure S3). DH36, DH61, and DH92 were adapted in environments with similar conditions to those in 2013,D, and not adapted in 2012,C. DH34, DH51, and DH55 were positively adapted to environment 2012,D. Lines DH128 and DH39, with medium TGW means (31.21 g and 32.34 g, respectively) and ASVs of 0.091 and 0.335, respectively, had the best genotype selection indices (16 and 19, respectively).

#### 2.2.4. Soluble Sugar Content (DUBOIS)

DUBOIS sugars ranged from 17.83 μg mg^−1^ dry mass (for DH72 in 2013,D) to 181.66 μg mg^−1^ dry mass (for DH9 in 2013,D), with an average of 68.35 μg mg^−1^ dry mass (Table S4). Lines DH7, DH9, and DH14 were positively adapted to environment 2013,C. DH53 and DH104 were adapted in environments with similar conditions to those in 2010,D, and not adapted in 2012,C and 2012,D (Figure S4). DH67 and DH80 were adapted in environments with similar conditions to those in 2010,C, and not adapted in 2012,C and 2012,D. Lines DH51 and DH2, with medium DUBOIS means (78.89 μg mg^−1^ dry mass and 77.8 μg mg^−1^ dry mass, respectively) and ASVs of 1.017 and 1.097, respectively, had the best genotype selection indices (22 and 32, respectively).

#### 2.2.5. Glucose

Glucose concentrations ranged from 2.37 μg mg^−1^ dry mass (for DH62 in 2013,C) to 49.87 μg mg^−1^ dry mass (for DH97 in 2013,D), with an average of 15.62 μg mg^−1^ dry mass (Table S5). Lines DH9, DH97, DH24, DH14, and DH143 were positively adapted to environment 2013,D. DH58 was adapted in environments with similar conditions to those in 2012,C, and not adapted in 2013,D (Figure S5). DH65 was adapted in environments with similar conditions to those in 2013,C, and not adapted in 2012,C. Lines DH60 and DH5, with high glucose mean concentrations (19.75 μg mg^−1^ dry mass and 18.44 μg mg^−1^ dry mass, respectively) and ASVs of 0.464 and 0.656, respectively, had the best genotype selection indices (23 and 37, respectively).

#### 2.2.6. Fructose

Fructose concentrations ranged from 0.02 μg mg^−1^ dry mass (for DH55 in 2010,C) to 74.62 μg mg^−1^ dry mass (for DH14 in 2013,D), with an average of 15.48 μg mg^−1^ dry mass (Table S6). Lines DH14, DH9, DH97, and DH143 were positively adapted to environment 2013,D. DH39, DH71, and DH31 were adapted in environments with similar conditions to those in 2010,D, and not adapted in 2010,C (Figure S6). DH94 and DH104 were adapted in environments with similar conditions to those in 2013,C, and not adapted in 2010,D. Lines DH57 and DH5, with high fructose concentration means (21.6 μg mg^−1^ dry mass and 20.85 μg mg^−1^ dry mass, respectively) and ASVs of 0.893 and 0.752, respectively, had the best genotype selection indices (28 and 29, respectively).

#### 2.2.7. Sucrose

Sucrose concentrations ranged from 1.75 μg mg^−1^ dry mass (for SQ1 in 2012,D) to 149.02 μg mg^−1^ dry mass (for DH52 in 2010,D), with an average of 50.08 μg mg^−1^ dry mass (Table S7). Line DH86 was positively adapted to environment 2013,C. DH36, DH52, and DH104 were adapted in environments with similar conditions to those in 2010,D, and not adapted in 2012,D (Figure S7). SQ1 was adapted in environments with similar conditions to those in 2010,C, and not adapted in 2012,D. Lines DH30 and DH43, with high sucrose concentration means (61.81 μg mg^−1^ dry mass and 56.88 μg mg^−1^ dry mass, respectively) and ASVs of 0.635 and 0.219, respectively, had the best genotype selection indices (12 and 18, respectively).

#### 2.2.8. Maltose

Maltose concentrations ranged from 0.41 μg mg^−1^ dry mass (for DH41 in 2010,D and DH55 in 2010,C) to 47.41 μg mg^−1^ dry mass (for DH69 in 2012,D), with an average of 14.68 μg mg^−1^ dry mass (Table S8). Lines DH65, DH146, DH22, and DH128 were positively adapted to environment 2010,D. DH8, DH47, and DH51 were adapted in environments with similar conditions to those in 2010,C, and not adapted in 2012,D (Figure S8). DH87, DH2, DH97, and DH14 were positively adapted to environments 2012,C, 2013,C, and 2013,D. Lines DH127 and DH53, with medium maltose concentration means (18.82 μg mg^−1^ dry mass and 18.04 μg mg^−1^ dry mass, respectively) and ASVs of 0.479 and 0.632, respectively, had the best genotype selection indices (21 and 24, respectively).

### 2.3. Total Genotype Selection Index

The best total genotype selection index (for all eight traits combined) was observed for lines DH39 (TGSI = 392), DH51 (TGSI = 481) and DH57 (TGSI = 486), and the worst for lines DH49 (TGSI = 1620), DH144 (TGSI = 1093), and DH37 (TGSI = 996.5). Lines DH39, DH51 and DH57 would be suitable for further inclusion in breeding programmes due to their better stability in comparison with other lines and good average values for the observed traits.

### 2.4. Phenotypic Associations Amongst Agronomic and Sugar Traits

Correlation analysis was carried out separately for control and drought treatments using the DHL data averaged across the three years (Table 3). Among the agronomic traits, yield was highly significantly correlated with biomass in both treatments, though yield was significantly correlated with TGW only in the control treatment. This finding implies that yield was determined largely through variation in plant grain number under drought conditions.

Several significant correlations were found between the agronomic traits and various leaf sugars, though many significant correlations were treatment-dependent. Thus, under control conditions, yield was significantly associated with only leaf fructose concentration, whereas under drought conditions, yield was highly significantly associated with both glucose and fructose concentrations. Biomass was significantly associated with fructose and glucose concentrations under control conditions, but with all leaf sugar concentrations, except sucrose, under drought conditions. The same sugars were significantly associated with TGW under control conditions, though TGW was correlated with no sugar contents under drought conditions (Table 3).

Amongst the sugar traits, correlations for both glucose and fructose with other sugars were similar in both treatments. DUBOIS sugars were significantly correlated with all other sugars in both control and drought treatments. Sucrose was highly significantly correlated with only DUBOIS sugars in both treatments. Maltose showed highly significant correlations with other sugars under control conditions, though correlations were much weaker with other sugars under drought conditions (Table 3).

### 2.5. DArTseq-Extended Genetic Map

The first genetic map for CS×SQ1 contained 567 AFLP, RFLP, SSR, morphological, and biochemical markers [24]. This map was next supplemented with 472 unique DArT (Diversity Array Technology) markers in the second version [25]. For this study, a further 3586 SNP and 10288 silicoDArT polymorphic markers were included to improve saturation of the genetic map used by Czyczylo-Mysza et al. [25].

Within the linkage groups, markers were ordered and placed in bins having the same marker scores for each CSDH line, resulting in 1106 unique DArTseq marker segregations. For individual chromosomes, selected representative DArTseq markers were integrated with the previous version of the map [25], and missing marker data were filled in based on the genotype of neighbouring loci. The obtained map comprising 1564 markers was used for *QTL* identification.

### 2.6. QTL Analyses and Reliability of QTL Detection Using 90 DHLs

As skewness for trait frequency distributions averaged across years (Table 2) was less than 1.0 in absolute terms and trait kurtosis was typically less than 1.0 in absolute terms, trait data each year approximated normal distributions and were, therefore, suitable for *QTL* mapping without transformation.

Collectively, results of the phenotypic–genotypic comparisons (Table S10) and DH population size effect (Table S11) showed a relatively small impact of population size within the range of 75 to 91 DHLs on *QTL* detection, especially for *QTLs* with large LOD scores.

The phenotypic and genotypic correlation coefficients amongst traits showed considerable similarity (Table S10, three spreadsheets “Phenotyp + genetic…”). This was particularly true for pair-wise phenotypic trait correlations identified as significant at both anthesis and dough (data points identified in green in scatter plots), which were matched by similar high correlation coefficients between the same traits on the basis of LOD scores. This was also true for significant phenotypic correlations between anthesis and dough traits. Thus, highly correlated traits at the phenotypic level were reflected by several regions of coincident high LOD score peaks at the genetic level.

Regarding DH population size effects (Table S11), depending on the LOD score criterion used for declaring a *QTL*, between 5% and 15% *QTLs* identified using the full DHL dataset of 91 DHLs were missing from the reduced DHL dataset. Conversely, the reduced DHL dataset identified around 10% false *QTLs* compared with the full dataset. Thus, using LOD scores of only ≥1.20 as the criterion for declaring the presence of a *QTL* gave only around 10–15% missing and false *QTLs* for the large majority of trait comparisons. For both dataset comparisons, the LOD score peaks identified in one dataset were almost always clearly present in the other dataset, even though they did not reach the declared levels of significance of ≥1.50 and ≥1.20 (Table S11). Note that the selection of LOD score peaks of ≥1.2 and ≥1.5 for the comparison of full and reduced DHL *QTL* analyses does not imply that they represent *significant* *QTLs*.

The location of *QTL* peaks along the chromosomes was largely unaffected by population size, with the large majority (81%) of *QTLs* being at the same location or within 2 cM for full and reduced datasets. Peaks for a further 12% *QTLs* were 4 or more cM from the full dataset *QTL*, and the remaining 7% *QTLs* did not reach a LOD score of 1.0 in the full dataset (Table S11).

Both phenotypic–genotypic comparisons and DH population size effects demonstrated that, even with only around 75 DHLs for the *QTL* analysis of some traits, the majority of *QTLs* identified showed co-localisations between traits that were phenotypically correlated and which were also present with datasets using both the full and reduced number of DHLs for *QTL* analysis.

By studying the frequency of *QTL* LOD score maxima for a particular trait occurring at the same genetic map (cM) location (Table S12), we were able to identify several candidate genes for a range of agronomic, developmental, morphological, and physiological traits. Thus, for example, *QTL* peak maxima for plant height were frequently found exactly at the location of the qualitatively scored gene *Rht-B1*, and flowering time and frost resistance *QTLs* were frequently located exactly at the qualitatively scored *Vrn1-5A* locus. No LOD score peaks were in cells adjacent to the cM cell containing the candidate gene for 13 of 24 candidate genes in Table S12.

Therefore, we consider that in the context of *QTL* discovery and comparisons, our CSDH mapping population used in this present study, although relatively small by current standards, was still large enough to justify comparisons amongst traits not only phenotypically but also genotypically, and the extensive marker redundancy allowed bins of known bp length to be developed, thus facilitating candidate gene discovery.

#### 2.6.1. Localisation of *QTLs* for Agronomic Traits

*QTL* analysis allowed the identification of regions associated with yield components: Yld—yield per plant, Bio—biomass of the above-ground parts of the plant, and TGW—thousand grain weight, across 15 chromosomes: 1A, 1D, 2A, 2B, 3A, 4A, 4B, 4D, 5A, 5B, 5D, 6A, 6D, 7A, and 7D, with a predominance of *QTLs* detected under optimal water conditions (23/40). In total, 40 *QTLs* for yield and its components were mapped using CIM with 1000 permutations to determine threshold levels (Table 4). Their distribution across the genomes was as follows: 18 *QTLs* in the A genome, 12 *QTLs* in the B genome, and 10 *QTLs* in the D genome. *QTLs* conditioning productivity traits were most frequently located on chromosome 5B (seven *QTLs*) and chromosome 7A (seven *QTLs*), with chromosome 5D having four *QTLs* and chromosomes 2A, 4B and 6A each having three *QTLs*. In the control group of plants, chromosomes 5B and 7A each had five *QTLs*, and, under drought stress, chromosomes 4B and 5D both had three *QTLs*. The mapped *QTLs* explained variation in yield components with LOD scores ranging from 3.13 to 7.77 (Table 4).

Comparison of agronomic trait *QTLs* with the full dataset of *QTLs* from trials in both field and pot environments showed that the majority of *QTLs* for agronomic traits (23 of 40) were also present in at least one field trial of the CSDH lines (Table 4 and Table S9). In particular, *QTLs* for yield in our 3-year pot trials were frequently found (≥3 trials) in field trials at markers *3952240* (1D), *978479* (1D), *wmc177* (2A), *Rht-B1* (4B), *cfd7* (5D), *gwm635b* (7A), and *5050390* (7A). LOD scores and additive effects for *QTLs* reported in Table 4 are shown along each chromosome in Figure S9.

#### 2.6.2. *QTLs* for Yield (Yld)

CIM analysis identified a total of 13 *QTLs* at LOD scores ranging from 3.13 to 5.36 across six chromosomes: 1D, 2A, 4B, 5B, 5D, and 7A. Under optimal water conditions, nine *QTLs* were mapped across four chromosomes, with the highest number found on chromosome 7A (four *QTLs*). Under drought stress, four *QTLs* dispersed amongst the three genomes were identified. The coefficients of determination ranged from 9.23% to 17.94% (Table 4).

#### 2.6.3. *QTLs* for Biomass (Bio)

A total of 13 *QTLs* with LOD scores ranging from 3.30 to 6.56 were identified across ten chromosomes: 1A, 2B, 4A, 4B, 5A, 5B, 5D, 6A, 7A, and 7D. Under well-watered conditions, six *QTLs* were mapped across five chromosomes, with two *QTLs* found on chromosome 5A. Under drought stress, seven *QTLs* were mapped across five chromosomes, with two *QTLs* located on each of chromosomes 4B and 5D. The coefficients of determination ranged from 8.74% to 23.21% (Table 4).

#### 2.6.4. *QTLs* for Thousand Grain Weight (TGW)

CIM analysis of thousand-grain weight identified a total of 14 *QTLs* at LOD scores ranging from 3.26 to 7.77 across eight chromosomes: 2A, 2B, 3A, 4D, 5B, 6A, 6D, and 7A. Under optimal irrigation, eight *QTLs* were mapped across five chromosomes, with two *QTLs* found on each of chromosomes 2A, 4D, and 5D. Under drought stress, six *QTLs* were mapped across five chromosomes, with two *QTLs* located on chromosome 7A. The coefficients of determination ranged from 11.89 to 31.26% (Table 4).

#### 2.6.5. *QTLs* for Flag Leaf Soluble Sugar Contents (DUBOIS)

A total of nine *QTLs* were identified for Dubois sugar contents, with similar numbers of *QTLs* under optimal irrigation (five *QTLs*) and drought conditions (four *QTLs*). Two *QTLs* were mapped under optimal irrigation on 7A (Table 4).

#### 2.6.6. *QTLs* for Individual Water-Soluble Carbohydrates (WSC)

The mapping of *QTLs* for water-soluble carbohydrates in the flag leaf identified a total of 44 *QTLs* across the three experimental years, covering all chromosomes, except 3B, 3D, 4D and 6A, with LOD scores ranging from 2.8 to 7.5, but for maltose above 12.9, and *R*^2^ ranging from 8.4 to 40.0, the highest again for maltose. The highest total number of *QTLs* was mapped on chromosomes 6B (nine *QTLs*) and 5B (seven *QTLs*). Most *QTLs* (29/44) had a negative additive allele effect (namely, the increasing allele came from SQ1). Under optimal irrigation, 17 *QTLs* were mapped, with the highest number found on chromosome 6B (five *QTLs*), and 27 *QTLs* were mapped under drought stress across seven chromosomes of the A, B, and D genomes, with the highest number located on chromosome 5B (seven *QTLs*).

#### 2.6.7. *QTLs* for Flag Leaf Glucose Content

The mapping of *QTLs* for glucose content in the flag leaf resulted in eight *QTLs* across the three years, located on chromosomes 1B, 2A, 2D, 5B, 6B, and 7D, with LOD scores ranging from 2.8 to 5.5, and *R*^2^ values ranging between 10.29 and 17.42. The highest number of *QTLs* was mapped on chromosome 5B (two *QTLs*), with a predominance of *QTLs* detected under drought conditions (75%). Five *QTLs* exhibited a negative additive allele effect (increasing allele from SQ1). Two *QTLs* were mapped under optimal irrigation, and six under drought stress, with two *QTLs* located on chromosome 5B.

#### 2.6.8. *QTLs* for Flag Leaf Fructose Content

*QTL* mapping for fructose content in the flag leaf identified 16 *QTLs* across the three years, located on chromosomes 1B, 2B, 2D, 4A, 4B, 5A, 5B, 5D, 6B, 7A, and 7D, with LOD scores ranging from 3.2 to 6.2, and *R*^2^ values above 10% for all *QTLs* (10.52–21.04), with a predominance of *QTLs* detected under drought conditions (62.5%). The highest number of *QTLs* was mapped on chromosomes 6B (five *QTLs*), with three *QTLs* located on both 5B and 7D. Eleven *QTLs* exhibited a negative additive allele effect (increasing allele from SQ1). Six *QTLs* were mapped under optimal water conditions, with the highest number found on chromosome 6B. Ten *QTLs* were found under drought stress, with three on chromosome 5B.

#### 2.6.9. *QTLs* for Flag Leaf Sucrose Content

Mapping of *QTLs* for sucrose content in the flag leaf led to 11 *QTLs* being identified over the three trial years, located on chromosomes 1A, 2B, 3A, 4A, 5A, 5B, 6B, with LOD scores ranging from 3.42 to 7.52, and *R*^2^ values calculated to be between 9.75 and 26.98. The majority (6/11) of *QTLs* were detected under drought conditions, and five *QTLs* exhibited a positive additive allele effect (increasing allele from Chinese Spring). Two *QTLs* were present on chromosome 5A under optimal irrigation, and two *QTLs* were on 4A under drought stress.

#### 2.6.10. *QTLs* for Flag Leaf Maltose Content

Across the three years, nine *QTLs* for maltose content in the flag leaf were mapped to chromosomes 1D, 2B, 4A (two *QTLs*), 5B, 5D, 6B, 6D and 7A, with LOD scores ranging from 3.94 to 12.94, and *R*^2^ values between 13.07 and 39.96, with similar numbers of *QTLs* detected under both optimal irrigation conditions (four) and drought stress (five). Seven *QTLs* exhibited a negative additive allele effect (increasing allele from SQ1).

### 2.7. Coincident QTLs for WSC, Individual Carbohydrates, and Agronomic Traits

Composite interval mapping (CIM) analysis substantiated the existence of genomic regions that were associated with more than one trait, either agronomic or sugar-related (Table 5). Considering all eight traits across the 3-year trials and optimal watering and drought stress treatments, 21 regions of the genome had two or more trait *QTLs* no more than 5 cM apart. Six genomic regions were identified where *QTLs* for glucose and fructose concentrations were coincident: distal on chromosome 1B, marker *7491713* (*QGLU(C13).csdh-1B*, *QFRU(C13).csdh-1B*), on 2D at marker *978402* (*QGLU(D12).csdh-2D*, *QFRU(D12).csdh-2D*), on 4B at marker *Rht-B1* (*QGLU(D10).csdh-4B*, *QFRU(D10).csdh-4B*), on 5B at two locations, markers *3950923* (*QGLU(D13).csdh-5B.1*, *QFRU(D13).csdh-5B.1*) and *1125268* (*QGLU(D13.2).csdh-5B*, *QFRU(D13).csdh-5B.2*), and chromosome 6B at marker *wmc397* (*QGLU(D12).csdh-6B*, *QFRU(C12).csdh-6B*). Four of those locations were also coincident with *QTLs* for other traits: yield on 4B—*QYld(D12).csdh-4B* at *Rht-B1*, TGW on 5B—*QTGW(C10).csdh-5B* at markers *3950923* (*QTGW(D10).csdh-5B.1*) and *112526* (*QTGW(C10).csdh-5B.2*), and Dubois sugars on 6B at marker *wmc397* (*QDUB(C10).csdh-6B*).

*QTLs* for agronomic traits were also frequently coincident either with *QTLs* for other agronomic traits or with *QTLs* for leaf sugar contents. Thus, *QTLs* for yield and TGW were located at closely linked markers *psr332* and *wmc177* on chromosome 2B (*QYld(D13).csdh-2A* and *QTGW(C13).csdh-2A*). Biomass and fructose content *QTLs* (*QBio(D10).csdh-4A*, *QFRU(D13).csdh-4A*) were coincident on chromosome 4A at marker *dupw004a*. Chromosome 5A had two closely spaced clusters of *QTLs* for biomass and sucrose, the first around markers *995510* and *m43p78.9a_5A* (*QBio(C13).csdh-5A.1*, *QSUC(C10).csdh-5A.1),* and the second 10 cM distal at markers *2334260* and *1205781* (*QBio(C13).csdh-5A.2*, *QSUC(C10).csdh-5A.2*).

Two other coincident *QTL* clusters were present for yield on chromosome 5B, around markers *1028405* and *wPt*-9814: *QYld(D12).csdh-5B* and *QBio(C13).csdh-5B* for yield and biomass, respectively, and at marker *psp3037*: *QYld(C13).csdh-5B* and *QFRU(D13).csdh-5B* for yield and fructose content, respectively. *QTLs* for yield under both control and droughted conditions in 2012 were coincident around markers *cfd7* and *cfd3* on chromosome 5D (*QYld(C12).csdh-5D*, *QYld(D12).csdh-5D*). Yield *QTLs* were also associated with *QTLs* for both TGW and leaf maltose content on chromosome 7A in a 7 cM region from markers *gwm635b* to *2260931* (*QYld(C13).csdh-7A.1*, *QYld(C13).csdh-7A.2*, *QTGW(D13).csdh-7A* and *QMAL(C10).csdh-7A*). *QTLs* for biomass and leaf fructose content were located on chromosome 7D around markers *2249010* and *barc154 QBio(C13).csdh-7D*, *(QFRU(D13).csdh-7D).*

Five other regions of coincident *QTLs* were specific for leaf sugar contents; three were for sucrose and maltose contents: *QSUC(D10).csdh-4A.2* and *QMAL(D10).csdh-4A.2*, respectively, on 4A near marker *1210223*, *QSUC(D13).csdh-5B* and *QMAL(D13).csdh-5B* on 5B at marker *m72p78.3*, and *QSUC(D13).csdh-6B* and *QMAL(D13).csdh-6B* on 6B around markers *wPt-6247* and *6037846*. This 6B location was also coincident with the fructose content *QTL* *QFRU(D12).csdh-6B*. The other pairs of coincident sugar *QTLs* were both for fructose and sucrose, on chromosome 2B (*QSUC(C10).csdh-2B* and *QFRU(C10).csdh-2B* associated with markers *5411238* and *gwm429* and 6B (*QSUC(C10).csdh-6B* and *QFRU(C12).csdh-6B)* linked with markers *wPt-4164* and *4407762*.

## 3. Discussion

One of the principal abiotic constraints limiting ecosystem productivity is reduced water availability associated with low soil moisture potential [26]. Drought stress constitutes a multifaceted physiological phenomenon impacting plants at biochemical, physiological, anatomical, and morphological levels, thereby impairing both yield quantity and quality. Under water-deficit conditions, drought stress elicits alterations in the accumulation and metabolism of soluble carbohydrates, particularly fructans, which play a pivotal role in modulating plant productivity and stress tolerance mechanisms [3,4,18,27,28,29,30,31,32].

Under conditions of water deficit, plants exhibit significant shifts in the concentration of soluble carbohydrates, with fructans representing the predominant sugar species implicated in modulating yield performance [3,4,18,27,28,29,30,31,32]. Substantial genotypic variation in soluble sugar content has been documented across wheat (*Triticum aestivum* L.) genotypes, particularly in the stem [4,8,33] and, to a lesser extent, in the grain [34]. Notably, the majority of quantitative trait loci (*QTL*) mapping efforts related to sugar metabolism in wheat have focused predominantly on stem carbohydrate accumulation [3,18,35,36,37,38,39], with limited attention directed toward carbohydrate dynamics in flag leaves.

Findings from the present study, focusing on ethanol-soluble sugars in flag leaves in response to a short-term drought during the stem extension and early grain filling phases, showed a significant increase in fructose content of around 50% (Table 2). Other studies focusing on the peduncle have also found a notable increase in fructose concentrations. potentially reflecting enhanced mobilisation of stored carbohydrates mediated by upregulated fructan exohydrolase activity, facilitating the hydrolysis of fructans into sucrose for subsequent translocation to sink organs [40,41].

Drought stress also resulted in overall elevated concentrations of glucose in the flag leaves by 31% (Table 2). These drought-induced increases in fructose and glucose were mirrored by a 20% reduction overall in leaf sucrose concentrations. Nevertheless, both total soluble sugars according to Dubois (Table 2), and the sum of fructose, glucose, sucrose, and maltose contents were essentially identical in both control and drought treatments (95.7 and 95.9 µg mg^−1^ dry mass for control and drought, respectively, Table 2), implying little impact overall of the drought treatment on the photosynthetic apparatus. Therefore, over the three-year experiment, the lower flag leaf sucrose contents under drought conditions would imply a drought-induced inhibition of the flag leaf phloem transport mechanism in response to drought during the rapid grain filling phase. Even so, Tables S4–S8 indicate that genotype sugar contents responded differently to the drought treatment across the three years of trials.

The accumulation of hexoses such as glucose and fructose under drought stress has also been observed in rice leaves and wheat spikes [42]. This accumulation is likely attributable not only to the hydrolytic breakdown of sucrose or raffinose but may also involve altered metabolic fluxes and sugar transport dynamics. Nguyen et al. [43] proposed that the accumulation of hexoses under drought conditions may be attributed to the diminished activity of enzymes involved in glycolysis, the primary metabolic pathway responsible for the degradation of simple sugars. Another contributing factor to the enhanced sugar content in donor tissues during drought stress is the reduction in plant height and the concomitant decline in starch synthesis [44], and plant height was reduced consistently in droughted plants in our experiments (data not presented).

During the early stages of pollen development, the predominant soluble sugars in wheat spikes include sucrose, glucose, fructose, and various fructans [45,46]. Both glucose and fructose play essential roles in the grain-filling process. The initial step in the conversion of sucrose to starch in developing grains involves the hydrolysis of sucrose into hexoses [47]. Immature, green wheat spikes are characterised by elevated levels of maltose and maltotriose, with a sharp decline in their concentrations as the spikes approach maturity [48]. Notably, even in mature, ungerminated grains, the presence of maltose can still be detected [49]. This may be due to its role as a transient product of enzymatic starch hydrolysis or its formation through direct conversion of monosaccharides, possibly involving precursor molecules.

In our research, we found no significant effect of the drought treatment on flag leaf maltose concentrations either overall (Table 2) or in any of the three years (Table S8). Although drought has been shown to increase leaf maltose contents on some occasions [50], a study by Živanović et al. [51] on tomato leaves found no significant effect of drought on leaf maltose concentrations, and although Vergara-Diaz et al. [52], in a metabolome profiling study of wheat, found an increase in leaf maltose contents in response to drought at anthesis, no significant effects of drought stress on leaf maltose contents were found during grain filling (same heat map colours).

The agronomic traits yield, biomass and TGW in our experiments were significantly correlated with flag leaf sugar traits, though the significance of correlations varied with the treatment (Table 3). Yield and biomass were more strongly associated with flag leaf sugar concentrations, especially glucose and fructose, under drought conditions than under well-watered conditions. This implies a strong dependence of grain filling on current assimilate under drought conditions. Also note that these highly significant correlations between glucose and fructose concentrations in the main stem flag leaves and plant yields were despite flag leaf sugar concentrations being measured in only the main stem flag leaf and yield being determined for each plant without its main stem ear. This implies close coordination of flag leaf glucose and fructose concentrations in all productive tillers.

Nevertheless, both phenotypic correlation analysis and *QTL* analysis showed that yield and TGW were, surprisingly, determined independently of leaf concentrations of sucrose, the sugar transported through the phloem to the developing ear. Although leaves were sampled within only a 2 h period each year, leaf sugar concentrations in wheat flag leaves show diurnal fluctuations [53] that vary according to the particular sugar, phase of plant development, and variety. Thus, both glucose and fructose in flag leaves sampled 14 d after anthesis increased around only 24% from 12:30 to 16:30 (mean of two varieties), whereas leaf sucrose concentrations increased over four-fold during the same period [53]. Thus, sucrose concentrations measured in our experiments may have been affected by significant diurnal variation during the 2 h sampling period.

Yield was associated with TGW only weakly under control conditions and not at all under drought conditions (Table 3), consistent with our drought treatments starting during the early phase of floret development. Thus, yield variation amongst DHLs was determined largely by variations in grain number per plant rather than grain filling, which was evidently compensated for once droughted plants had been rewatered.

Although some researchers have found no direct relationship between sugar contents and yield components [54,55,56], research on abiotic stress tolerance in *Dactylis glomerata* (orchardgrass) has shown a significant correlation between the accumulation of water-soluble carbohydrates (WSC) and biomass production [57]. Additionally, Foulkes et al. [29] demonstrated a positive correlation between soluble sugar concentrations in the wheat stem and grain yield, both under drought stress and irrigated conditions. In our research, we also identified highly significant positive correlations between yield and both glucose and fructose concentrations in flag leaves during grain filling, especially under droughted conditions (Table 3), which complements the strong association between water-soluble carbohydrates (WSCs) in stems and yield in wheat reported by others.

This population has been extensively characterised at both the genotypic and phenotypic levels, as evidenced by prior studies [24,25,58,59,60] on diverse agronomic and physiological traits, such as yield components, plant height, chlorophyll content, and fluorescence parameters, and we have demonstrated in Tables S10–S12 that a population of only 90 DHLs can be used to identify *QTLs* reliably.

Our comprehensive analysis of carbohydrate profiles and yield-related traits under different water availabilities presented here enabled us to identify several stable *QTLs* (*QTL*s present across different years, treatments, and traits). The very high correlations between flag leaf glucose and fructose concentrations (Table 3) were reflected in coincident *QTLs* for glucose and fructose concentrations at six locations: 1B, 2D, 4B, 6B, and two locations on 5B, where monosaccharide *QTLs* were also coincident with *QTLs* for TGW. In addition, on chromosome 7D, QTls for glucose and fructose contents were separated by only 6 cM, *QGLU(D13).csdh-7D* and *QFRU(C13).csdh-7D*, respectively. These were located in the *QTL* region for accumulation efficiency of stem water soluble carbohydrates *QAeswc.cgb-7D.1* reported by [18].

These *QTL* clusters for flag leaf glucose and fructose contents indicate tight coordination of the production and metabolism of these monosaccharides and their role in supporting grain filling. Despite sugar concentrations being measured in flag leaves on different stems from those sampled for yield, some *QTLs* for yield (and in a few cases also for biomass) were coincident with *QTLs* for flag leaf sugar contents: the *Rht-B1* locus on 4B and *psp3037* on 5B (Table 5). This further supports the key role of flag leaf monosaccharide contents in grain filling and explains the highly significant correlations in Table 3 between yield and both glucose and fructose, especially under drought conditions. Similar coordination of the flag leaf concentrations of the disaccharides sucrose and maltose was also evident at the genotypic level, with coincident *QTLs* for the two traits at three locations (chromosomes 4A, 5B, and 6B, Table 5), though none of these *QTL* regions was coincident with any agronomic trait *QTLs*.

Mapping genomic regions for yield and agronomic traits under drought stress has been previously conducted in various cereal species, including barley [61], sorghum [62], rice [63], maize [64], and many studies in diverse wheat populations under varying soil water regimes [3,24,25,58,59,65,66,67]. The following discussion on *QTLs* that exhibit a stable effect on the regulation of yield, its associated agronomic traits and sugar contents focuses on chromosomes where we found coincident *QTLs* for several agronomic traits (4B, 5B, 5D, and 7A), as well as a major cluster of *QTLs* for flag leaf sugar contents on 6B, in relation to other genetic studies reported for wheat and associated candidate genes.

The major reduced-height marker *Rht-B1* on chromosome 4B was directly connected with yield, glucose, and fructose contents under drought conditions (Table 5). Previous studies have also shown regions on chromosome 4B, including the gene *Rht-B1*, associated with biomass in bread wheat, both in well-hydrated and drought conditions and fructose content under drought conditions [3,24,58,59,68]. The largest *QTL* for height was located at the major dwarfing gene *Rht-B1* on 4B chromosome [58], and it is well established that these *Rht* dwarfing alleles cause a reduced response to the gibberellic acid class of plant hormones, which influence stem elongation. The shortened stature may contribute to improved water-use efficiency, while simultaneously facilitating the remobilisation of water-soluble carbohydrates (WSCs), including fructose, from vegetative tissues to the developing grain. These findings align with previous studies reporting the dual role of *Rht* genes in structural and metabolic drought responses.

On chromosome 5B in our study, marker *3950923* was associated with the yield component TGW as well as glucose and fructose contents (Table 5). Our search for candidate genes at this location on 5B (Table S13) identified 91 genes in a 9.19 Mb fragment (Chr5B: 516,041,582–525,230,556 bp), including a gene (*TraesCS5B03G0831200*) for a Myb-related protein Hv1, that could be responsible for kernel size [69]. Another 5B locus around marker *wPt-9814* with *QTLs* for both yield and biomass (Table 5) was located close to a candidate gene for TGW (*TraesCS5B02G044800*) identified by Zhao et al. [70]. The *QTL* cluster for Yld and fructose content on 5B at marker *psp3037* was only 2 cM from a *QTL* peak maximum for grain length identified by Cerit et al. [71], though the homologous location for an effect on grain length was proximal to our 5D *QTL* cluster for yield (Table 5). A candidate gene search around marker *psp3037* between 514 and 517 Mbp on 5B (Table S13) identified 26 annotated genes, including two 1-phosphatidylinositol phosphodiesterase genes (*TraesCS5B03G0830200* and *TraesCS5B03G0829800*) and water stress-related BTB/POZ (*TraesCS5B03G0825500*) corresponding with MATH domain-containing protein 2 (*TraesCS5B03G0825500*). In particular, 1-phosphatidylinositol phosphodiesterases play key roles in plant growth, development, and stress responses (see Ali et al.) [72]. Elsewhere on chromosome 5B, the *QTL* cluster for TGW, glucose, and fructose under droughted conditions, around markers 1144428 to 1125268 (spanning 529 to 539 Mbp), was found to be associated with 155 genes, including two coding for *NAC* genes (*TraesCS5B03G0893300* and *TraesCS5B03G0893200*) for NAC domain-containing protein 86 and NAC domain-containing protein 45. NAC domain-containing protein 86-like could be involved in regulating carbohydrate metabolism [73], and NAC domain-containing protein 45 has an important role in modulating plant stress responses [74].

A region on 5D at markers *cfd7* and *cfd3*, where yield *QTLs* *QYld(C12).csdh-5D and QYld(D12).csdh-5D* were present, was found to be homologous with the *QTL* cluster for TGW, glucose and fructose contents on 5B around markers 1144428 to 1125268, where two NAC domain-containing candidate genes were identified (Table S13). Thus, amongst 80 genes identified at that 5D locus, a gene for an NAC domain-containing protein 86 (*TraesCS5D03G0812500*, the 5D homologue of *TraesCS5B03G0893300*) gave the highest number of connected genes (Table S13). The biological process regulated by this gene is sieve element enucleation and differentiation (http://wheat.cau.edu.cn/TGT/ann_db/?geneID=TraesCS5D01G360800, accessed on 17 July 2025). A genetic analysis of wheat stem anatomy using the CSDH population (Rancic et al., manuscript in preparation) identified a *QTL* (CIM with 25 background markers) of maximum LOD score 5.3 for phloem area of large sieve vessels in wheat stems (mean of upper and lower stem sections sampled over two years) exactly at marker *cfd7* on 5D.

A candidate gene search for the sucrose-maltose *QTL* cluster on chromosome 6B at markers *wPt-6247* and *6037846* (120–123 Mbp, 2.76 Mbp) identified 21 genes, with the highest scoring gene, connected to 152 other genes, being *TraesCS6B03G0302700* encoding a sucrose transport protein SUC1 (Table S13). Candidate gene searches for other *QTL* clusters on 6B were not realistic because of large bp distances in the *QTL* regions. Nevertheless, *QTLs* for flag leaf fructose content, *QFRU(D10).csdh-6B* and *QFRU(C12).csdh-6B*, around markers *4910887* and *3064436* corresponded to *QTLs* for stem water-soluble carbohydrate at maturity stage (SWSC) *QSwscm.cgb-6B.1* found by Yang et al. [18].

Chromosome 7A in our trials included a *QTL* for yield and another for maltose content under well-watered conditions associated with marker 2260931. Using the same population of DHLs, Quarrie et al. [24] reported the involvement of this region on 7A with *QTLs* for spike dry weight, grain number, and biomass, and with yield under both well-watered and various stress conditions, including nutrient deficiency, drought, and salinity. Furthermore, in the present study, a *QTL* for maltose content under well-watered conditions was identified within this region. Although we found no reports of *QTLs* for leaf maltose content on chromosome 7A, Tura et al. [75] reported a *QTL* for maltose coincident with a *QTL* for yield on chromosome 1B and proposed that the 1B effects on yield might be due to the accumulation of maltose and fructose in leaves that could be translocated to grain during grain filling.

With regard to chromosomal location of some of the key genes involved in fructan metabolism, genes for fructan 1-exohydrolase (*1-FEH*) are located on the group 6 chromosomes, genes for fructan 6-exohydrolase (*6-FEH*) are located on group 2 chromosomes, and genes for sucrose:sucrose 1-fructosyltransferase (*1-SST*), sucrose:sucrose 6-fructosyltransferase (*6-SFT*), and fructan:fructan 1-fructosyltransferase (*1-FFT*) are all located on the group 7/4A chromosomes. Collectively, these genes regulate the synthesis and degradation of fructans—the major class of water-soluble carbohydrates (WSCs) in wheat stems [76,77]. However, none of these genes for fructan metabolism enzymes was located near any *QTL* regions we identified for flag leaf sugar concentrations. In addition to enzymes involved in fructan metabolism, sugar transporter genes such as *Triticum aestivum* sucrose transporter (*TaSUT1*, group 4 chromosomes, and *TaSUT2*, group 2 chromosomes) and their transcriptional regulators (e.g., *TaMYB*) have been identified as key components in carbohydrate partitioning and long-distance movement of WSCs, especially during stress conditions. Previous studies have shown that expression of *TaSUT1* genes is upregulated under water deficit, enhancing sink strength and improving yield stability [9,78]. However, none of these sugar transporter genes was located near a sugar content *QTL* in our study.

The ANOVA results in this study demonstrated that genotype, environment, and their interaction (GEI) significantly influenced all measured traits. The environment accounted for the largest proportion of variation in most traits—up to 74.4% in biomass—whereas GEI effects were particularly prominent in sugar-related traits, especially glucose and fructose. These findings align with previous reports indicating that environmental factors have a dominant influence on yield components in wheat. For example, Jędzura et al. [79] reported that environmental effects explained 89.0% of grain yield variation, while genotype and GEI accounted for 4.3% and 5.2%, respectively [79]. Moreover, the significant GEI effects observed in our study underscore the complex interplay between genotype performance and environmental variability. Pluta et al. [80] similarly highlighted that GEI significantly influenced key quality traits such as plant height, number of stems, average number of spikes, average spike length, average number of spikelets per spike, average number of seeds per spike, average number of seeds per plant, seed weight per plant, thousand kernel weight, and average stem filling with pith in wheat, suggesting that certain genotypes are more stable across variable environments [80]. Principal component analysis (PCA) further revealed that the first two interaction principal components (IPCA1 and IPCA2) captured the majority of the interaction variance, consistent with findings from other AMMI-based studies. For example, Bayisa et al. [81] found that IPCA1 and IPCA2 explained 60.11% and 28.47% of the GEI variance, respectively. In contrast, Bocianowski et al. [82] found that the first interactive principal component explained from 59.02% to 99.51% of the total variability.

These results demonstrate the importance of AMMI analysis and support the idea that selecting genotypes based on both performance and stability indices is essential for improving drought resilience and carbohydrate-related traits in wheat. The presence of transgressive segregation and large phenotypic variation among DHLs indicates a high potential for selecting genotypes better adapted to water-limited conditions. We have also demonstrated the tight association between yield and several sugars analysed in flag leaves during the phase of rapid grain filling, especially under droughted conditions.

The detection of *QTLs* within the same chromosomal region across different environments provides strong evidence for their practical application, particularly in marker-assisted selection (MAS). Investigating the phenotypic variability of yield components and soluble sugar contents under contrasting soil moisture conditions, in combination with *QTL* analysis, enabled us to identify genomic regions that independently influence the traits of interest and, for cases of consistent additive effects, these are likely to exhibit pleiotropic action. Regions that account for a greater proportion of phenotypic variance are especially valuable, as they may be utilised in the selection of drought-tolerant cultivars and in identifying candidate genes.

To exploit our findings from these glasshouse pot experiments for MAS would require demonstration that our stable *QTLs* for yield would also be expressed under field conditions. Table 4 demonstrated that the majority of yield *QTLs* were also present on at least one occasion in field trials, and that *QTLs* for yield in field trials were found frequently (≥3 occasions) on chromosomes 1D (two loci), 2A, 4B, 5D, and 7A (two loci). The 4B locus at *Rht-B1* has been the focus of breeding programmes for many years, though the 5D locus for yield around *cfd7*, which was associated with a gene affecting sieve element differentiation, could be a useful target for further study.

## 4. Materials and Methods

### 4.1. Plant Material

The study material consisted of a population composed of 90 doubled haploid (DH) lines (CSDH), obtained from the cross between Chinese Spring (CS) and SQ1 (a genotype with an increased content of abscisic acid (ABA) under drought conditions) [24], the two parents differing in both morphology and physiology [24,25,58,60] as well as in sucrose content under the conditions of soil water deficit [83].

### 4.2. Experimental Design and Plant Growth

Ninety DHLs of the CSDH mapping population and their parental lines, CS and SQ1, were studied for three years (2010, 2012, and 2013). The seedlings of 90 CSDH lines and the two parents, Chinese Spring (CS) and SQ1, after 6 weeks of vernalisation, were placed in pots (Ø15 cm, 20 cm high, one seedling per pot) filled with a mixture of soil and sand in equal proportions by volume. At the beginning of the experiment, pots were filled with the same mass of soil (1.7 kg) and water content. A few pots were chosen to determine soil FWC (field water capacity). Pots were watered with the same amount of water and weighed. On the basis of plant vigour and appearance of the soil, plants were watered with the appropriate volume of water, approximately, for drought and control treatments. In our study, 20–25% FWC was adopted to give a severe drought and well-watered controls were maintained at 65–70% FWC. Every few days, the weights of some pots were controlled to determine the mass of water needed for watering, depending on whether pots were watered more frequently or rarely during the experiment. Three plants as replicates per line and treatment were grown, to give six plants per CSDH line and the parents. The plants were grown in an open-sided vegetation tunnel, partially covered with transparent foil to exclude rainfall. This setup allowed exposure to natural light and ambient temperature conditions (May–September), closely mimicking field atmospheric conditions while enabling controlled drought stress application. At the rapid tillering (2010 and 2012) or rapid stem elongation (2013) stage, a strict limitation of watering was applied to the plants destined to be subject to severe drought stress. Drought stress was continued for four weeks. On the last day of the drought-stress treatment, the main stem of each plant was removed at the flag leaf node, and the flag leaf blade was collected for biochemical measurements. The peduncle and developing ear were also sampled for biochemical analysis, to be reported in a separate publication. Sampling for biochemical parameters started around 12:00–13:00 each year and took *ca* 2 h in total.

### 4.3. Biochemical Measurements

The frozen flag leaves of main shoots of control plants and of those subject to drought conditions were lyophilised for 72 h, then powdered in a ball grinder MM 400 mixing mill (Retsch, Kroll, Germany). Samples with masses suitable for a particular biochemical analysis were weighed on a micro-analytical balance.

### 4.4. Measurement of Soluble Sugar Content (Dubois) Using a Spectrophotometric Method

Soluble sugars were extracted from *ca* 6 mg powdered samples by shaking in 95% ethyl alcohol in a MM 400 mixing mill (15 min, 30 Hz), then the extract was centrifuged (2100× *g*, 10 °C, 15 min). The total carbohydrate content was determined using the phenol-sulphuric acid method [84] using a Synergy II microplate reader (BioTek, Winooski, VT, USA). Measurements of absorbance were made at *λ* = 490 nm.

### 4.5. Measurement of Selected Water-Soluble Carbohydrates Content (WSC) Using Liquid Chromatography

Concentrations of selected water-soluble sugars (glucose, fructose, sucrose and maltose) were measured according to the modified method of Janeczko et al. [85] using a liquid chromatograph (Agilent 1200 with Coulochem II amperometrical detector, Agilent Technologies, Santa Clara, CA, USA). Sugars were extracted from 10 mg of powdered sample in 1 mL of deionised water for 1 h in a rotatory shaker at 50 rpm. After that, samples were centrifuged, supernatant collected, and diluted with acetonitrile (1/1 *v*/*v*), again centrifuged and after filtration (0.22 μm nylon membrane), used for HPLC analysis. The separation of sugars was performed on a Hamilton RCX-10 250 × 4.1 mm column with 80 mM NaOH (Hamilton, Reno, NV, USA). Sugars were detected amperometrically, with the same waveform as used by Janeczko et al. [85].

### 4.6. Agronomic Traits

Yield parameters were evaluated when the plants had reached full maturity. For this purpose, grain yield per plant (YP), dry weight per plant at harvest (Biomass—B) and thousand-grain weight (TGW) were measured.

### 4.7. Genotypic Measurements

The genetic map previously published by Czyczylo-Mysza et al. [25] was augmented with DArT markers. For these, DNA was isolated from 2-week-old seedlings using the method of Milligan [86]. Concentrations were determined on a Qubit 2.0 fluorimeter (Life Technologies Co., Carlsbad, CA, USA). Wheat DArTseq 1.0 analyses (0.8 million reads/sample) were performed as a service (Diversity Arrays Technology Pty Ltd., Bruce, Australia) for 90 DHLs and Chinese Spring (CS) and SQ1 parents. Data obtained with DArTseq technology yielded two types of markers, i.e., silicoDArT and SNPs. Illumina-sequenced DNA fragments from each genotype obtained after restriction enzyme digestion were analysed bioinformatically. A matrix of silicoDArT markers was created based on the presence or absence of sequences, while a pool of SNP markers was created based on the presence of mutations in fragments between genotypes.

Markers detecting heterozygosity above 5%, with missing data above 30% and with minor allele frequency below 20% were excluded from the analyses. Finally, a total of 3586 SNPs and 10288 silicoDArT markers were mapped (BLASTN, https://galaxy-web.ipk-gatersleben.de/, accessed on 31 July 2025) at e-value < 3.38 × 10^−6^ to the Chinese Spring reference genome (IWGSC CS RefSeq v2.1, GCF_018294505.1). Markers meeting the quality criteria were clustered and assigned to chromosomes based on physical location. Within the linkage groups, redundant markers were sorted out, and singletons were corrected. Further, markers were sorted using the maximum likelihood method in Join Map (Van Ooijen, 2006) [87] using markers with a known physical location as the “fixed order”. Once the linkage group was established, physical locations of misassigned markers were corrected (BLASTN) based on searches on the URGI platform [88]. The resulting DArTseq marker segregations for individual chromosomes were integrated with the data for the previous version of the map [25], and missing marker data were filled in based on the genotype of neighbouring loci. The resulting genetic map is shown in Figure S9 with marker bp locations in Chinese Spring RefSeq v2.1 included.

### 4.8. Statistical and QTL Analyses

Statistical analyses for standard deviation, skewness, and kurtosis were performed using Genstat 23. The data were analysed using the additive main effects and multiplicative interaction (AMMI) model [89] for each trait independently. The AMMI model first fits the additive effects for the main effects of genotypes (G) and environments (E), followed by multiplicative effects for genotype-by-environment interaction (GEI) using PCA. The results of the AMMI analysis are presented as biplot graphs. The AMMI model [90] is expressed using the following formula:

$$y_{ge}=\mu +\alpha_{g}+\beta_{e}+\sum_{n=1}^{N}\lambda_{n}\gamma_{gn}\delta_{en}+Q_{ge}$$

where $y_{ge}$ is the trait mean of genotype *g* in environment *e*, μ the grand mean, $\alpha_{g}$ the mean genotype deviation, $\beta_{e}$ the mean year deviation, *N* the number of PCA axes retained in the adjusted model, $\lambda_{n}$ the eigenvalue of the PCA axis *n*, $\gamma_{gn}$ the genotype score for the PCA axis *n*, $\delta_{en}$ the score eigenvector for the PCA axis *n*, and $Q_{ge}$ is the residual, including the AMMI noise and pooled experimental error. The AMMI stability value (ASV) was used to compare the stability of genotypes as described by Purchase et al. [91]:

$$ASV=\sqrt{{\left[\frac{{SS}_{IPCA1}}{{SS}_{IPCA2}}\left({IPCA}_{1}\right)\right]}^{2}+{\left({IPCA}_{2}\right)}^{2},}$$

where ${SS}_{IPCA1}$ is the sum of squares for IPCA1, ${SS}_{IPCA2}$ the sum of squares for IPCA2, and the IPCA_1_ and IPCA_2_ scores are the genotype scores in the AMMI model. A lower ASV score indicates a more stable genotype across environments [92]. The genotype selection index (GSI), calculated for each genotype, incorporates both the trait mean and the ASV index in a single criterion (GSIi), as follows [93,94]:

$${GSI}_{i}={RM}_{i}+{RA}_{i},$$

where ${RM}_{i}$ is the rank of the trait mean (from maximum to minimum) for the *i*-th genotype, and ${RA}_{i}$ is the rank of the *ASV* for the *i*-th genotype. Finally, the total genotype selection index (TGSI) was calculated for each genotype as the sum of the ${GSI}_{s}$ for all eight traits [90]. All analyses were conducted using the GenStat v. 23 statistics software [95].

Linear correlation coefficients between individual traits were calculated based on the mean values for each DHL across the three years, separately, for the control and drought conditions.

Phenotypic data for productivity traits and biochemical analyses were used to locate *QTLs* in 90 lines of the CSDH population by composite interval mapping (CIM), performed with Windows *QTL* Cartographer v.2.5 software [96]. A *QTL* locus was identified in a region designated by its maximum LOD score, and declared significant if it exceeded a critical value, determined by 1000 permutations (typically LOD > 3.3). Windows *QTL* Cartographer graphics options were used to generate chromosome linkage group genetic maps which were combined with graphics of trait LOD score traces and additive effects along each chromosome to generate Figure S9.

### 4.9. QTL Frequencies for Grain Yield, TGW, and Aboveground Biomass from Other CSDH Population Trials

To determine the co-location of *QTLs* for yield, TGW and biomass in these experiments with *QTLs* for these agronomic traits under a wider range of growing conditions in both field and pot trials, phenotypic data from other trials with the CSDH population in which Yld, TGW, and aboveground biomass at maturity (Bio) were recorded (Table S9 and references cited therein) were used for *QTL* analysis. Yld, TGW, and Bio were recorded in 56, 53, and 31 trials, respectively, with 38 in the field, 26 in pots, and one in a soil glasshouse, in several countries. *QTL* analysis of Yld, TGW and Bio using CIM was carried out with the extended genetic map as described above using mean CSDH line data for each trial/treatment. At each marker and 2 cM interval between markers along the chromosomes, LOD score maxima of ≥2.0 were identified and totalled separately for both field and pot trials across all trials/treatments. In this way, frequencies of occurrence of *QTLs* for Yld, TGW, and Bio for plants growing under a range of field and pot conditions could be tested for coincidence with *QTLs* for Yld, TGW, and Bio in our experiments reported here.

### 4.10. Candidate Gene Searches for Key QTL Regions

For selected *QTLs* from 5B, 5D, and 6B, corresponding physical regions on chromosomes of IWGSC wheat v.2.1 were retrieved. Genes within these *QTLs* were identified at wGRN (http://wheat.cau.edu.cn/wGRN/, accessed on 17 July 2025) [97] (Table S13).

### 4.11. Impact of Population Size on QTL Detection

The genetic map of the CS×SQ1 DH population was generated using only 95 lines, and in this study, only 90 of the DHLs were used for *QTL* analysis. Therefore, because of the risk of identifying false *QTLs* with only 90 DHLs, and due to the Beavis effect (98), which results in greatly overestimated *QTL* phenotypic variances when only a few progeny are evaluated, we carried out three independent methods to assess the validity and reliability of our *QTL* analyses.

For two methods, we used phenotypic data and *QTL* analysis using *QTL* Cartographer from another trial with the same population of DHLs and a genetic map of only 702 informative markers, where only 71–80 DHLs were available for traits sampled at the dough stage. For this trial, 91 DHLs with two replicate plants per DHL were vernalised and grown at one plant per 2 L pot under glasshouse conditions using the design and growth conditions described in Habash et al., 2007 [58].

The following organs were sampled at both anthesis (main shoot) and soft dough stage of grain filling (primary tiller): flag leaf lamina (FLL), flag leaf sheath (FLS), penultimate leaf lamina (PLL), whole ear (ear), and the whole peduncle (ped). Leaf refers to the sum of leaf organs FLL, FLS, and PLL. For each organ, after drying at 80 °C, the following traits were recorded: dry weight (dwt), organ %C, organ %N and from these, total organ elemental C, and elemental N contents were calculated (dwt x %C, and dwt x %N).

#### 4.11.1. Comparison of Phenotypic and Genotypic Trait Correlations

As phenotypic correlations among traits would reflect gene effects on more than one trait that are pleiotropically linked, the first method examined the strength of correlations between trait phenotypic means and trait LOD score maxima from interval mapping (IM), on the basis that two traits significantly correlated phenotypically should also have coincident *QTLs*. For this, a representative dataset of LOD scores was prepared, using organ traits (dwt, C, N, %C, %N) at anthesis, having significant correlations with at least one organ trait (dwt, C, N, %C, %N) at the dough stage as the criterion for traits to select (see Table S10, “Phenotypic correlation matrix”, correlation coefficients in coloured cells). This gave 18 traits at anthesis and 16 at dough (see Table S10, traits in red text in “Phenotypic correlation matrix”). Three sets of phenotypic and genotypic correlations were compared: 18 traits at anthesis and 16 traits at dough for pairwise correlations within the sampling occasion, and the complete set of 34 traits, looking only at correlations between a trait at anthesis and a trait at dough (see Table S10, spreadsheets “Phenotyp + genetic anth correlat”, “Phenotyp + genetic dough correlat”, “Phenotyp + genetic an vs. dou corr”).

#### 4.11.2. Comparison of *QTL* Detection with Full and Reduced DHL Datasets

The second method to test the effect of reduced population size on reliability of *QTL* detection used phenotypic data for 12 traits used in our previous study [58] to compare the impact on *QTL* locations and maximum LOD scores (using the genetic map of 702 informative markers) of reducing the full dataset of 91 DHLs to 75–78 DHLs, deleting as far as possible DHLs missing from the dough trait samples described in “Impact of population size on *QTL* detection”, above. The 12 traits are described in Table S11.

Peak LOD scores of ≥1.9, ≥1.5, and ≥1.2 for the 12 traits using the full and reduced DHL phenotypic datasets were collated and compared to assess the impact of the smaller population size dataset on the likelihood of missing *QTLs* from the full DHL dataset and getting false *QTLs* from the reduced dataset (Table S11).

#### 4.11.3. Candidate Genes Identified Using *QTL* Analysis with CSDH Population Trials

Since the CSDH population was developed, many trials in field and pot environments have been carried out, and a wide range of agronomic, developmental, physiological, and biochemical traits have been recorded (Table S9 and references cited therein).

Despite the relatively small DH population size, it has been possible to locate candidate genes for several traits by collating *QTL* analysis results from these various field and pot trials. For a particular trait, the frequency of finding *QTL* LOD score maxima of ≥2.0 at a specific genetic map location (in cM) amongst all trials for the trait was determined using 25 background markers to control for the genetic background with CIM. Using the genetic map and markers reported here, redundant markers (markers having identical allele scores in the DH population) were clustered into marker bins, and bin bp boundaries were defined according to bin markers with the lowest and highest bp locations in Chinese Spring RefSeq v2.1. This allowed genes of known bp location to be tested as candidates for phenotypic effects recorded in the CSDH population.

## Figures and Tables

**Table 1:** Analysis of variance of main effects and interactions for eight observed traits in 90 lines of doubled haploids, their parental forms (CS and SQ1) and variability explained (ve, in %).

Trait			Yield		Biomass		TGW		DUBOIS		Glucose		Fructose		Sucrose		**Maltose**	
Source of Variation		d.f.	Mean Square	ve	Mean Square	ve	Mean Square	ve	Mean Square	ve	Mean Square	ve	Mean Square	ve	Mean Square	**ve**	**Mean Square**	**ve**
Treatments		551	3.11 ***	91.30	9.96 ***	93.62	112.8 ***	75.27	2443 ***	85.02	231.3 ***	91.51	273.7 ***	91.46	2200 ***	93.07	220 ***	91.16
Genotypes, G		91	1.16 ***	5.63	6.11 ***	9.49	168.2 ***	18.54	2217 ***	12.74	297.6 ***	19.45	384 ***	21.19	1046 ***	7.31	205 ***	14.00
Environments, E		5	275.14 ***	73.23	871.8 ***	74.40	5134 ***	31.09	135,620 ***	42.84	9558.4 ***	34.32	9279 ***	28.13	172,584 ***	66.25	10,748 ***	40.37
Block		12	0.24	0.15	0.98 ***	0.20	45 **	0.65	672 ***	0.51	23.5 **	0.20	16.2	0.12	390 ***	0.36	62 ***	0.56
GE Interactions		455	0.51 ***	12.44	1.25 ***	9.75	46.5 ***	25.64	1024 ***	29.44	115.5 ***	37.75	152.7 ***	42.14	558 ***	19.51	108 ***	36.80
Interaction	IPCA 1	95	0.92 ***	37.40	1.95 ***	32.40	71 ***	31.84	2129 ***	43.39	251.9 ***	45.51	393 ***	53.72	1370 ***	51.22	246 ***	47.73
	IPCA 2	93	0.67 ***	26.79	1.62 ***	26.27	61.2 ***	26.87	1034 ***	20.62	142.2 ***	25.15	165.1 ***	22.10	666 ***	24.38	130 ***	24.60
	IPCA 3	91	0.5 ***	19.55	1.36 ***	21.72	48.6 ***	20.90	766 ***	14.95	96.5 ***	16.70	106.1 ***	13.89	288 ***	10.33	68 ***	12.71
	Residuals	176	0.22 ***	16.26	0.63 ***	19.44	24.5 **	20.39	557 ***	21.03	37.7 ***	12.64	40.6 ***	10.28	203 ***	14.08	42 ***	14.96
Error		1091	0.15		0.33		18.2		210		10.6		12.7		78		10	

** *p* < 0.01; *** *p* < 0.001; d.f.—the number of degrees of freedom; ve—variability explained (in %).

**Table 2:** Phenotypic performance for traits related to grain yield per plant (Yield), biomass, thousand-grain weight (TGW), ethanol (Dubois), and water soluble sugars of doubled haploid lines and their parents. Max and min are the maximum and minimum DH line mean data for each trait (mean from 3 years).

Traits		Treatments	Parents (Mean ± SD)		DH Lines				
			CS	SQ1	Mean	Min.	Max.	Skew.	Kurt.
Yield [g]		C	2.198 ± 1.15	2.343 ± 0.62	2.457	0.396	4.829	0.1767	−0.8287
		D	0.868 ± 0.74	0.879 ± 0.63	0.994	0.023	4.021	1.0566	1.6539
Biomass [g]		C	4.624 ± 2.15	4.098 ± 1.07	4.774	1.375	9.231	0.0522	−0.877
		D	2.529 ± 1.52	2.097 ± 0.82	2.321	0.181	7.692	0.9359	1.431
TGW [g]		C	25.92 ± 3.28	36.74 ± 6.17	30.97	11.31	84.00	0.6683	6.746
		D	19.65 ± 4.52	24.91 ± 6.82	24.75	10.82	47.00	0.3946	−0.211
Content [μg/mg dry mass]	Dubois	C	70.71 ± 33.6	76.42 ± 28.75	69.57	17.62	166.1	0.2663	−0.501
		D	78.38 ± 47.72	59.21 ± 40.43	66.98	14.24	201.0	0.9965	0.6114
	Glucose	C	13.46 ± 9.81	12.75 ± 6.72	13.59	1.876	41.73	0.6982	0.267
		D	11.02 ± 3.33	11.01 ± 3.97	17.80	2.062	58.50	1.0656	0.895
	Fructose	C	13.42 ± 10.17	12.78 ± 8.09	12.29	1.099	44.97	0.9562	1.513
		D	12.25 ± 4.03	11.83 ± 4.03	18.81	1.985	92.00	1.6023	3.872
	Sucrose	C	72.69 ± 34.67	64.18 ± 26.45	55.56	10.57	123.5	0.1962	−0.548
		D	62.29 ± 35.73	34.16 ± 33.65	44.22	5.19	174.7	0.9864	0.654
	Maltose	C	14.46 ± 9.84	15.27 ± 6.37	14.25	0.324	41.52	0.3251	−0.5207
		D	15.58 ± 3.94	15.27 ± 6.4	15.08	0.251	50.16	0.5101	0.3808

**Table 3:** Linear correlation coefficients between individual traits were calculated based on the mean values for the DH lines separately for the control (above the diagonal) and drought conditions (below the diagonal).

Trait	Yield	Biomass	TGW	DUBOIS	Glucose	Fructose	Sucrose	Maltose
Yield	1	0.824 ***	0.233 *	0.195	0.167	0.256 *	0.122	0.146
Biomass	0.857 ***	1	0.203	0.178	0.253 *	0.349 ***	0.019	0.127
TGW	0.018	0.032	1	0.318 **	0.382 ***	0.382 ***	0.017	0.226 *
DUBOIS	0.187	0.284 **	0.104	1	0.471 ***	0.499 ***	0.669 ***	0.456 ***
glucose	0.432 ***	0.493 ***	0.111	0.633 ***	1	0.948 ***	0.041	0.290 **
fructose	0.374 ***	0.455 ***	0.153	0.638 ***	0.962 ***	1	0.123	0.370 ***
sucrose	−0.118	0.007	0.185	0.596 ***	0.02	0.069	1	0.252 *
maltose	0.164	0.242 *	−0.04	0.230 *	0.248 *	0.249 *	0.097	1

Correlations are based on three-year means for each of 90 DH lines. Significance levels: * *p* < 0.05; ** *p* < 0.01; *** *p* < 0.001.

**Table 4:** Main characteristics of *QTLs* controlling yield per plant (YIELD), BIOMASS, TGW, ethanol and water soluble sugars, as well as individual sugars detected in 2010, 2012, and 2013 in the doubled haploid lines (CSDH) identified using CIM.

Traits	Treatment/Year	Chrom Group	Genome	*QTL*	Marker	Field *QTL* Peak No. **	Pot *QTL* Peak No.	*QTL* Position (cM) *	LOD Max.	R2 (%)	Additive
**YIELD**	**C/2010**	19	7A	*QYld(C_10_).csdh-7A*	*barc108LjK*	0	−2	151	4.00	14.50	−0.32
	**C/2012**	3	1D	*QYld(C_12_).csdh-1D.1*	*3952240*	7	3	61	4.01	12.55	0.24
		3	1D	*QYld(C_12_).csdh-1D.2*	*978479*	3	1	68	4.16	14.07	0.26
		15	5D	*QYld(C_12_).csdh-5D*	*cfd7*	6	2	107	4.39	14.03	0.25
	**C/2013**	14	5B	*QYld(C_13_).csdh-5B.1*	*psp3037*	−2	−1	75	5.36	17.94	−0.21
		14	5B	*QYld(C_13_).csdh-5B.2*	*1091024*	0	1	98	3.22	10.83	0.17
		19	7A	*QYld(C_13_).csdh-7A.1*	*gwm635b*	3	1	40	3.13	10.43	0.13
		19	7A	*QYld(C_13_).csdh-7A.2*	*2260931*	1	2	47	3.84	12.57	0.15
		19	7A	*QYld(C_13_).csdh-7A.3*	*5050390*	−5	−3	106	3.19	10.26	−0.13
	**D/2012**	11	4B	*QYld(D_12_).csdh-4B*	*Rht-B1*	4	10	56	3.17	9.25	0.17
		14	5B	*QYld(D_12_).csdh-5B*	*wPt-9814*	−1	−1	47	4.19	12.24	−0.20
		15	5D	*QYld(D_12_).csdh-5D*	*cfd3*	−1	−1	112	3.24	9.23	−0.18
	**D/2013**	4	2A	*QYld(D_13_).csdh-2A*	*wmc177*	−3	−5	62	3.24	10.20	−0.11
**BIOMASS**	**C/2010**	19	7A	*QBio(C_10_).csdh-7A*	*2255021*	0	−3	129	3.47	11.37	−0.50
	**C/2013**	5	2B	*QBio(C_13_).csdh-2B*	*5411238*	−2	−2	64	4.59	14.42	−0.30
		13	5A	*QBio(C_13_).csdh-5A.1*	*995510*	0	1	109	3.52	10.00	0.25
		13	5A	*QBio(C_13_).csdh-5A.2*	*2334260*	1	1	119	4.29	11.80	0.28
		14	5B	*QBio(C_13_).csdh-5B*	*1028405*	0	−2	45	4.94	13.80	−0.30
		21	7D	*QBio(C_13_).csdh-7D*	*barc154*	0	−2	57	6.07	17.70	−0.33
	**D/2010**	10	4A	*QBio(D_10_).csdh-4A*	*dupw004a*	−1	−1	60	4.20	14.49	−0.37
		16	6A	*QBio(D_10_).csdh-6A*	*5411292*	−1	−1	39	3.30	11.37	−0.32
	**D/2013**	1	1A	*QBio(D_13_).csdh-1A*	*5323867*	0	1	62	3.55	8.74	0.17
		11	4B	*QBio(D_13_).csdh-4B.1*	*wmc47*	0	1	101	4.05	13.72	0.35
		11	4B	*QBio(D_13_).csdh-4B.2*	*1020423*	0	−1	120	3.74	12.95	−0.27
		15	5D	*QBio(D_13_).csdh-5D.1*	*1091493*	0	1	207	4.47	16.46	0.33
		15	5D	*QBio(D_13_).csdh-5D.2*	*rPt-3825*	−1	−1	220	6.56	23.21	−0.46
**TGW**	**C/2010**	12	4D	*QTGW(C_10_).csdh-4D.1*	*1089425*	0	1	35	4.58	14.81	2.16
		12	4D	*QTGW(C_10_).csdh-4D.2*	*5328849*	1	1	41	3.80	12.65	1.94
		14	5B	*QTGW(C_10_).csdh-5B.1*	*3950923*	−1	−1	81	4.23	14.32	−2.06
		14	5B	*QTGW(C_10_).csdh-5B.2*	*4988916*	−2	−3	88	5.12	16.86	−2.22
	**C/2013**	4	2A	*QTGW(C_13_).csdh-2A.1*	*psr332*	0	1	60	4.40	15.56	3.19
		4	2A	*QTGW(C_13_).csdh-2A.2*	*991804*	0	−2	73	3.26	10.21	−2.61
		7	3A	*QTGW(C_13_).csdh-3A*	*4909617*	−1	−1	88	4.00	12.68	−1.93
		16	6A	*QTGW(C_13_).csdh-6A*	*1006957*	0	−3	62	4.20	13.53	−2.04
	**D/2010**	5	2B	*QTGW(D_10_).csdh-2B*	*m86p65.2_2B*	−1	−1	130	4.30	14.10	−3.32
		14	5B	*QTGW(D_10_).csdh-5B*	*1144428*	−2	−1	87	4.20	12.87	−1.97
		16	6A	*QTGW(D_10_).csdh-6A*	*3533299*	0	−2	67	3.90	11.89	−1.90
	**D/2012**	18	6D	*QTGW(D_12_).csdh-6D*	*1108725*	0	−2	165	4.40	15.53	−2.95
	**D/2013**	19	7A	*QTGW(D_13_).csdh-7A.1*	*4010137*	0	1	26	5.94	21.98	5.11
		19	7A	*QTGW(D_13_).csdh-7A.2*	*1097869*	−1	−1	43	7.77	31.26	−5.80
**DUBOIS**	**C/2012**	13	5A	*QDUB(C_10_).csdh-5A*	*979392*	n/a	n/a	39	3.67	11.68	6.17
		17	6B	*QDUB(C_10_).csdh-6B*	*wmc397*	n/a	n/a	71	4.30	13.31	−6.37
	**C/2013**	19	7A	*QDUB(C_13_).csdh-7A.1*	*3935390*	n/a	n/a	64	3.56	11.97	−8.63
		19	7A	*QDUB(C_13_).csdh-7A.2*	*1128327*	n/a	n/a	75	7.08	24.44	12.79
		20	7B	*QDUB(C_13_).csdh-7B*	*wPt-4309*	n/a	n/a	0	4.13	12.89	−5.42
	**D/2010**	3	1D	*QDUB(D_10_).csdh-1D*	*wPt-729826*	n/a	n/a	121	3.78	8.37	−6.72
		7	3A	*QDUB(D_10_).csdh-3A*	*995376*	n/a	n/a	107	5.24	14.62	8.75
		15	5D	*QDUB(D_10_).csdh-5D*	*2264251*	n/a	n/a	167	8.86	26.30	11.58
	**D/2012**	5	2B	*QDUB(D_10_).csdh-2B*	*3956411*	n/a	n/a	0.00	5.01	18.76	−7.06
**GLUCOSE**	**C/2012**	17	6B	*QGLU(D_12_).csdh-6B*	*wmc397*	n/a	n/a	71	5.14	15.19	−1.97
	**C/2013**	2	1B	*QGLU(C_13_).csdh-1B*	*7491713*	n/a	n/a	3	2.78	10.32	1.77
	**D/2010**	11	4B	*QGLU(D_10_).csdh-4B*	*Rht-B1*	n/a	n/a	51	4.99	17.42	3.59
	**D/2012**	6	2D	*QGLU(D_12_).csdh-2D*	*978402*	n/a	n/a	92	5.13	16.55	1.88
	**D/2013**	4	2A	*QGLU(D_13_).csdh-2A*	*GS2_463fxrt*	n/a	n/a	145	3.81	10.75	−3.60
		14	5B	*QGLU(D_13_).csdh-5B.1*	*3950923*	n/a	n/a	81	5.52	16.55	−4.31
		14	5B	*QGLU(D_13_).csdh-5B.2*	*1125268*	n/a	n/a	90	4.94	15.03	−4.11
		21	7D	*QGLU(D_13_).csdh-7D*	*3534296*	n/a	n/a	65	3.49	10.29	−3.32
**FRUCTOSE**	**C/2010**	5	2B	*QFRU(C_10_).csdh-2B*	*gwm429/3028596*	n/a	n/a	80	4.96	15.80	2.56
		17	6B	*QFRU(C_12_).csdh-6B*	*wPt-4164/4407762*	n/a	n/a	135	3.79	11.09	−2.13
	**C/2012**	17	6B	*QFRU(C_12_).csdh-6B.1*	*wmc397*	n/a	n/a	74	4.81	14.29	−1.55
		17	6B	*QFRU(C_12_).csdh-6B.2*	*3064436*	n/a	n/a	83	3.54	10.72	−1.37
	**C/2013**	2	1B	*QFRU(C13).csdh-1B*	*7491713*	n/a	n/a	3	3.51	12.44	2.71
		21	7D	*QFRU(C_13_).csdh-7D*	*wPt-0789/1276810*	n/a	n/a	71	3.19	11.62	−2.54
	**D/2010**	11	4B	*QFRU(D_10_).csdh-4B*	*Rht-B1*	n/a	n/a	55	3.43	10.52	3.04
		17	6B	*QFRU(D_10_).csdh-6B*	*wPt-4910887*	n/a	n/a	78	4.37	13.02	−3.39
	**D/2012**	6	2D	*QFRU(D_12_).csdh-2D*	*978402*	n/a	n/a	92	5.27	16.66	1.60
		17	6B	*QFRU(D_12_).csdh-6B*	*1250557*	n/a	n/a	66	4.77	13.64	−1.45
	**D/2013**	10	4A	*QFRU(D_13_).csdh-4A*	*dupw004a*	n/a	n/a	60	3.65	11.40	−4.78
		14	5B	*QFRU(D_13_).csdh-5B.1*	*psp3037*	n/a	n/a	75	4.59	16.11	−5.87
		14	5B	*QFRU(D_13_).csdh-5B.2*	*3950923*	n/a	n/a	81	6.24	21.04	−6.50
		14	5B	*QFRU(D_13_).csdh-5B.3*	*1125268*	n/a	n/a	90	4.40	15.55	−5.48
		21	7D	*QFRU(D_13_).csdh-7D.1*	*2249010*	n/a	n/a	52	4.17	14.00	−5.16
		21	7D	*QFRU(D_13_).csdh-7D.2*	*m71p77.7_7D/1079529*	n/a	n/a	176	3.61	11.09	5.84
**SUCROSE**	**C/2010**	5	2B	*QSUC(C_10_).csdh-2B*	*5411238*	n/a	n/a	76	4.67	15.87	2.38
		13	5A	*QSUC(C_10_).csdh-5A.1*	*m43p78.9a_5A*	n/a	n/a	113	6.74	20.82	2.77
		13	5A	*QSUC(C_10_).csdh-5A.2*	*1205781*	n/a	n/a	123	4.76	15.41	2.42
		17	6B	*QSUC(C_10_).csdh-6B*	*wPt-4164/4407762*	n/a	n/a	134	3.93	12.10	−2.05
	**C/2012**	7	3A	*QSUC(C_12_).csdh-3A*	*1255724*	n/a	n/a	63	7.52	26.98	−5.90
	**D/2010**	5	2B	*QSUC(D_10_).csdh-2B*	*4911226*	n/a	n/a	14	5.97	18.30	−16.69
		10	4A	*QSUC(D_10_).csdh-4A.1*	*m68p78.y*	n/a	n/a	120	4.21	11.77	8.95
		10	4A	*QSUC(D_10_).csdh-4A.2*	*1210223*	n/a	n/a	130	3.42	9.75	8.16
	**D/2012**	1	1A	*QSUC(D_12_).csdh-1A*	*4991333*	n/a	n/a	78	3.63	11.66	5.07
	**D/2013**	14	5B	*QSUC(D_13_).csdh-5B*	*m72p78.3*	n/a	n/a	6	4.42	15.58	−1.93
		17	6B	*QSUC(D_13_).csdh-6B*	*wPt-6247/6037846*	n/a	n/a	60	3.94	13.07	−1.70
**MALTOSE**	**C/2010**	5	2B	*QMAL(C_10_).csdh-2B*	*wmc257*	n/a	n/a	31	4.79	16.26	6.68
		19	7A	*QMAL(C_10_).csdh-7A*	*2260931*	n/a	n/a	47	4.82	16.38	5.63
	**C/2012**	3	1D	*QMAL(C_12_).csdh-1D*	*wPt-6316*	n/a	n/a	77	5.07	19.21	−1.69
	**C/2013**	18	6D	*QMAL(C_13_).csdh-6D*	*cfd49*	n/a	n/a	1	5.28	17.47	−1.61
	**D/2010**	10	4A	*QMAL(D_10_).csdh-4A.1*	*wPt-8275*	n/a	n/a	115	5.78	21.12	−4.97
		10	4A	*QMAL(D_10_).csdh-4A.2*	*1210223*	n/a	n/a	130	12.94	39.96	−6.92
	**D/2012**	15	5D	*QMAL(D_12_).csdh-5D*	*1863032*	n/a	n/a	55	4.29	13.11	−2.24
	**D/2013**	14	5B	*QMAL(D_13_).csdh-5B*	*m72p78.3*	n/a	n/a	6	4.42	15.58	−1.93
		17	6B	*QMAL(D_13_).csdh-6B*	*wPt-6247/6037846*	n/a	n/a	60	3.94	13.07	−1.70

* Position from the first marker on the chromosome short arm in cM. ** Field and Pot *QTL* Peak Number cells are shaded in red for increasing allele effects from SQ1 (negative LOD scores) and in green for increasing allele effects from CS (positive LOD scores). Intensity of shading is proportional to the *QTL* frequency of occurrence.

**Table 5:** Markers with coincident *QTLs* and associated phenotypic traits and trials.

Chromosome	Marker	Phenotypic Traits	Environment (Well-Watered—C, Drought—D)
1B	*7491713*	Glucose, fructose	C
2A	*psr322—wmc177*	Yield, TGW	C,D
2B	*5411238—gwm429*	Fructose, sucrose	C
2D	*978402*	Glucose, fructose	D
4A	*dupw004a*	Biomass, fructose	D
4A	*1210223*	Sucrose, maltose	D
4B	*Rht-B1*	Yield, glucose, fructose	D
5A	*995510—m43p78.9a_5A*	Biomass, sucrose	C
5A	*2334260—1205781*	Biomass, sucrose	C
5B	*m72p78.3*	Sucrose, maltose	D
5B	*1028405—wPt-9814*	Yield, biomass	C,D
5B	*psp3037*	Yield, fructose	C,D
5B	*3950923*	TGW, glucose, fructose	C,D
5B	*1144428—1125268*	TGW, glucose, fructose	C,D
5D	*cfd7-cfd3*	Yield	C,D
6B	*wPt-6247—1250557*	Fructose, sucrose, maltose	D
6B	*wmc397*	Dubois, glucose, fructose	C,D
6B	*wPt-4164—4407762*	Fructose, sucrose	C
7A	*gwm635b—1097869*	Yield, TGW	C,D
7A	*2260931*	Yield, maltose	C
7D	*2249010—barc154*	Biomass, fructose	C,D

## Data Availability

All data are contained within the article. The datasets used and analysed during the current study are available from the corresponding author on reasonable request.

## Supplementary Materials

The following supporting information can be downloaded at: https://www.mdpi.com/article/10.3390/ijms26167833/s1. References [98,99,100,101,102,103,104,105,106,107,108,109,110,111,112,113,114,115,116,117,118,119,120,121,122,123,124] are cited in the Supplementary Materials.
