# Supplementary material for: Impact of Soil Drought on Yield and Leaf Sugar Content in Wheat: Genotypic and Phenotypic Relationships Compared Using a Doubled Haploid Population
Source: Int J Mol Sci. 2025 Aug 13;26(16):7833. doi: 10.3390/ijms26167833 (PMC12386748; doi:10.3390/ijms26167833)
Supplement: Supplementary file 1 [file ijms-26-07833-s001.zip › Fig S9_QTLAB SQ 14 Aug 1.pdf]

Figure S9: The location of markers in both the genetic map (cM) and physical map (bp), together with LOD score traces and additive effects along each chromosome for all QTLs detailed in Table 4 (together with LOD traces for chromosomes 3B and 3D which had no trait LOD scores reaching significance). Markers with bp locations in red text are estimated by linear interpolation from neighbouring markers. Positive additive effects indicate increasing alleles from Chinese Spring and negative additive effects indicate increasing alleles from SQ1.

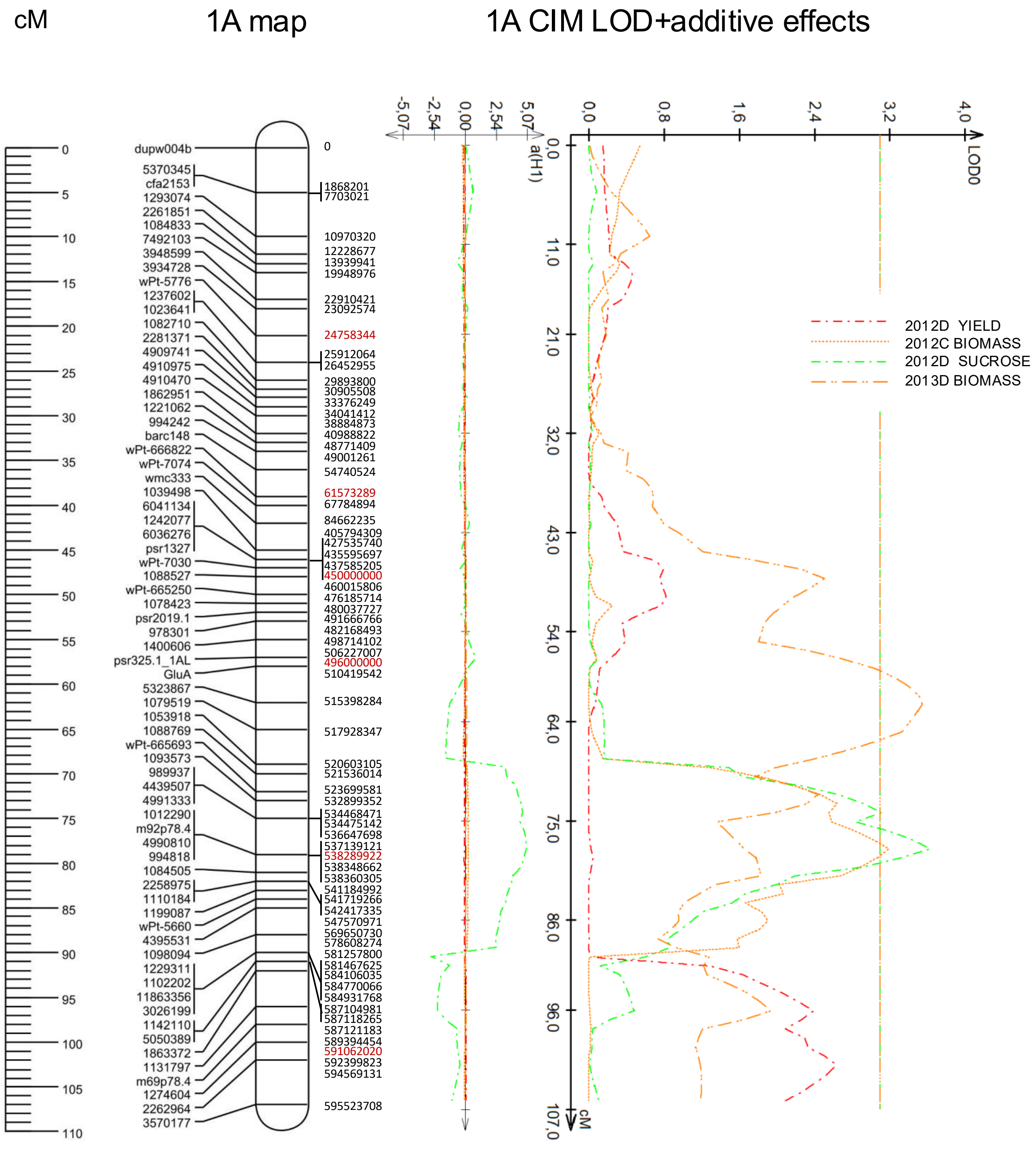

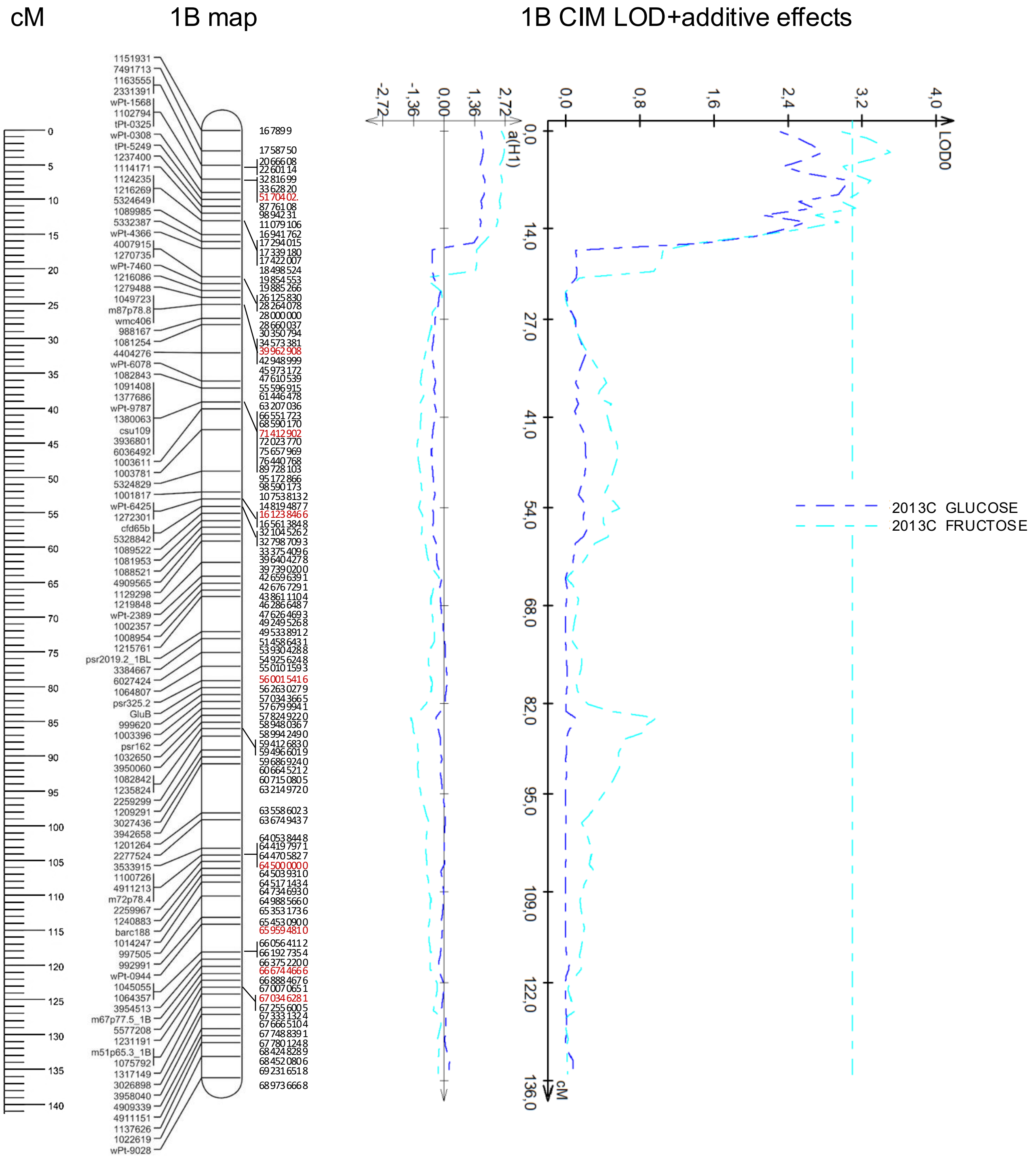
cM
1B map
1B CIM LOD+additive effects
a(H1)
LOD0
cM
2013C GLUCOSE
2013C FRUCTOSE

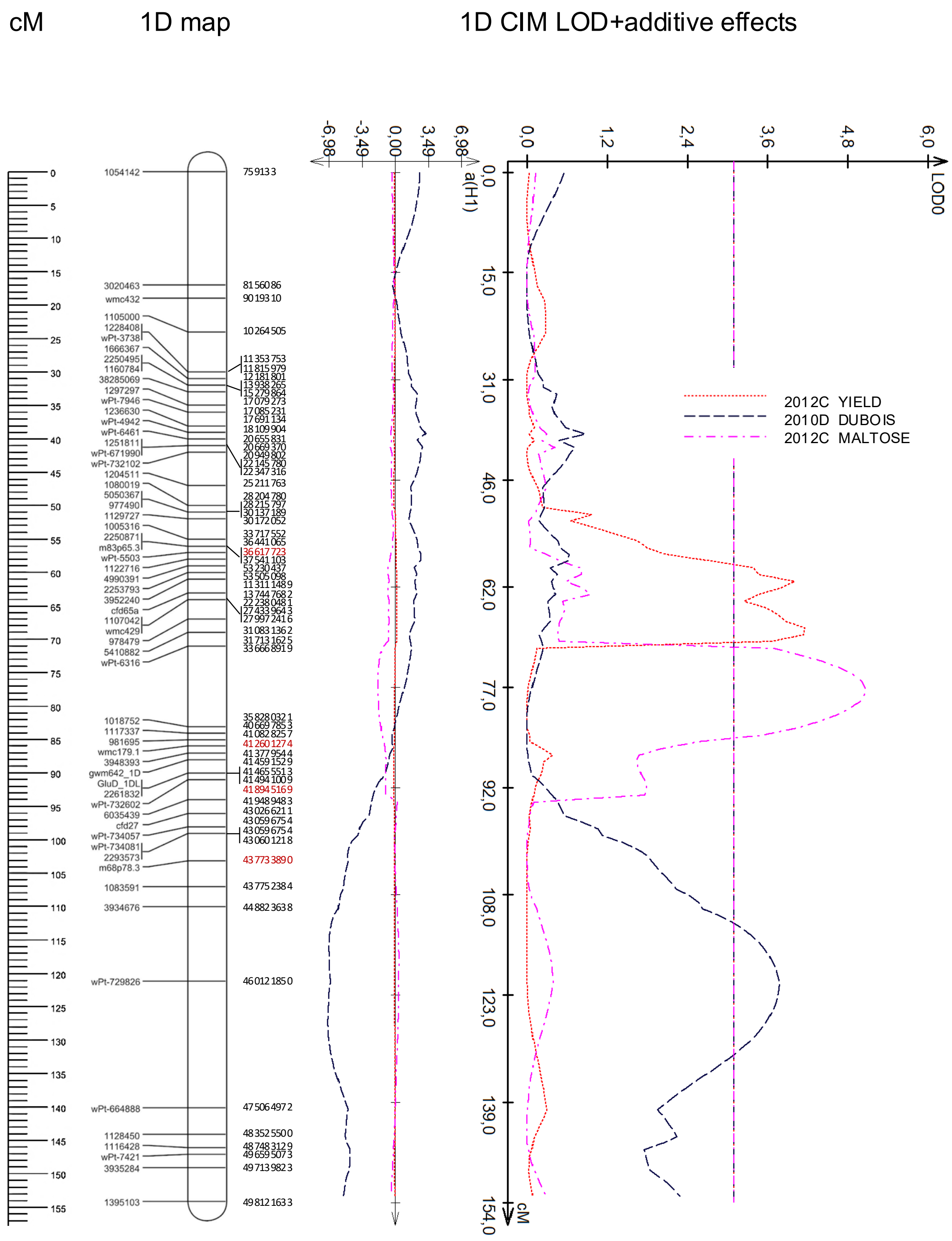
cM
1D map
1D CIM LOD+additive effects
2012C YIELD
2010D DUBOIS
2012C MALTOSE
LOD0
a(H1)
cM

cM 2A map 2A CIM LOD+additive effects

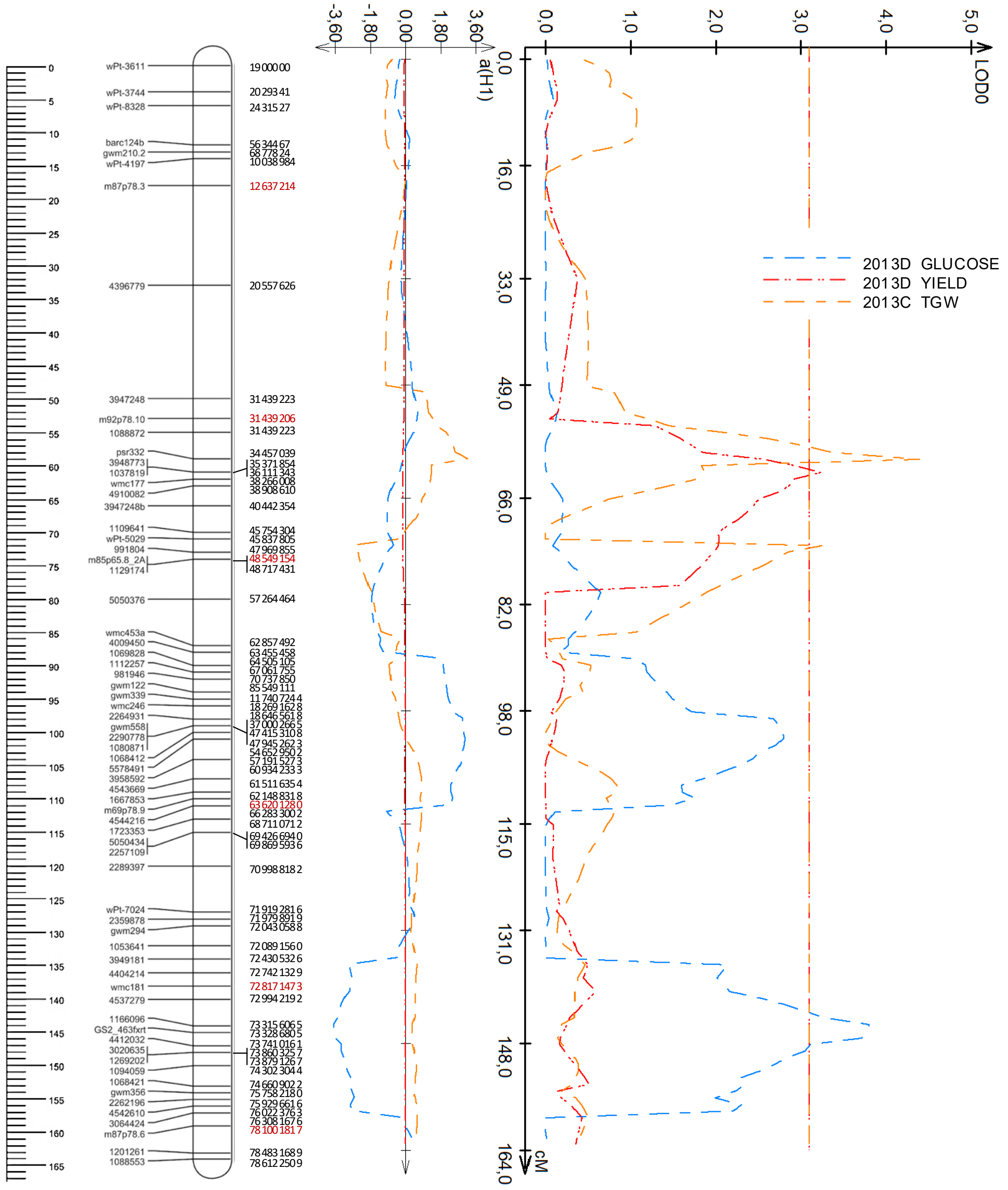

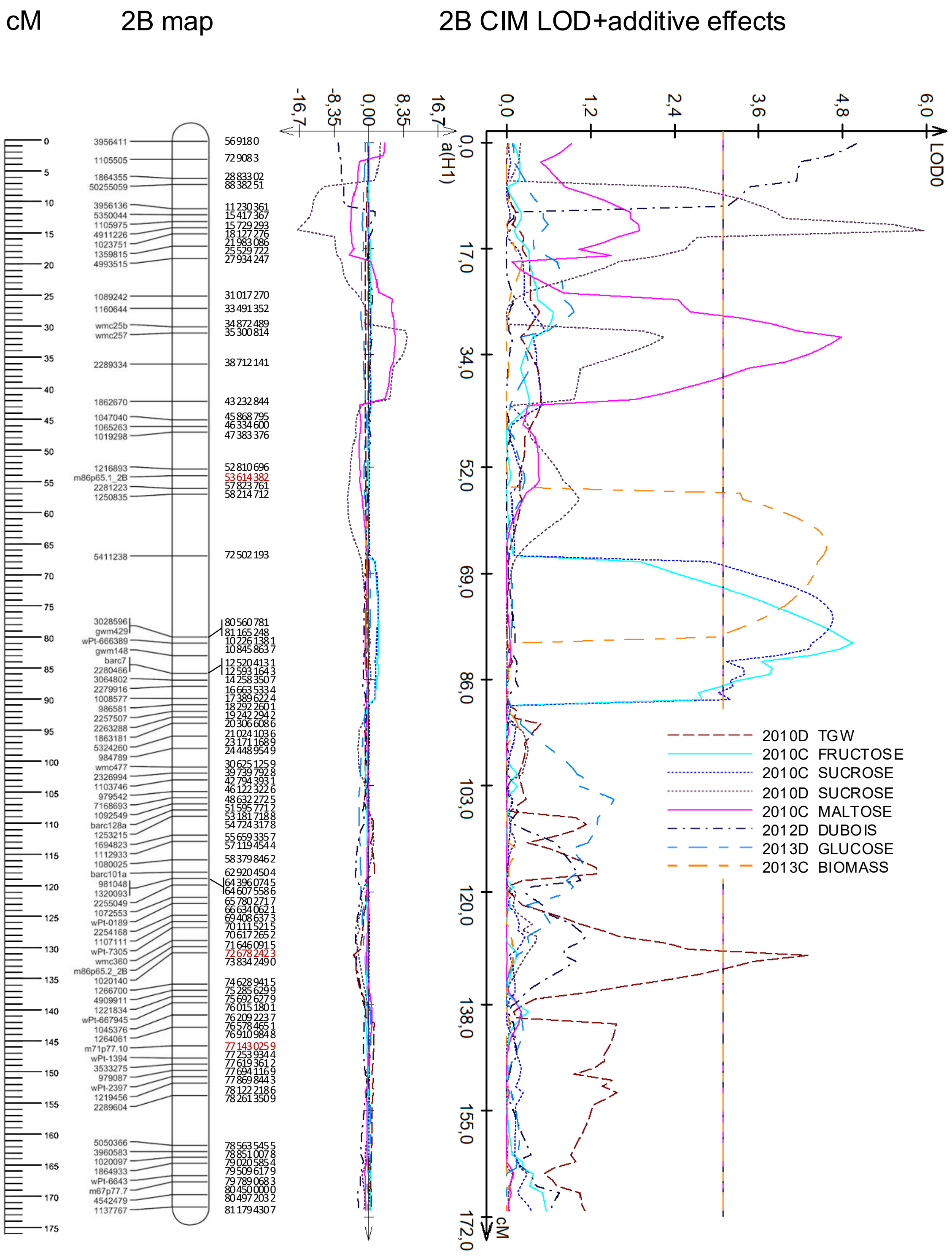
cM
2B map
2B CIM LOD+additive effects
a(H1)
LOD0
cM
2010D TGW
2010C FRUCTOSE
2010C SUCROSE
2010D SUCROSE
2010C MALTOSE
2012D DUBOIS
2013D GLUCOSE
2013C BIOMASS

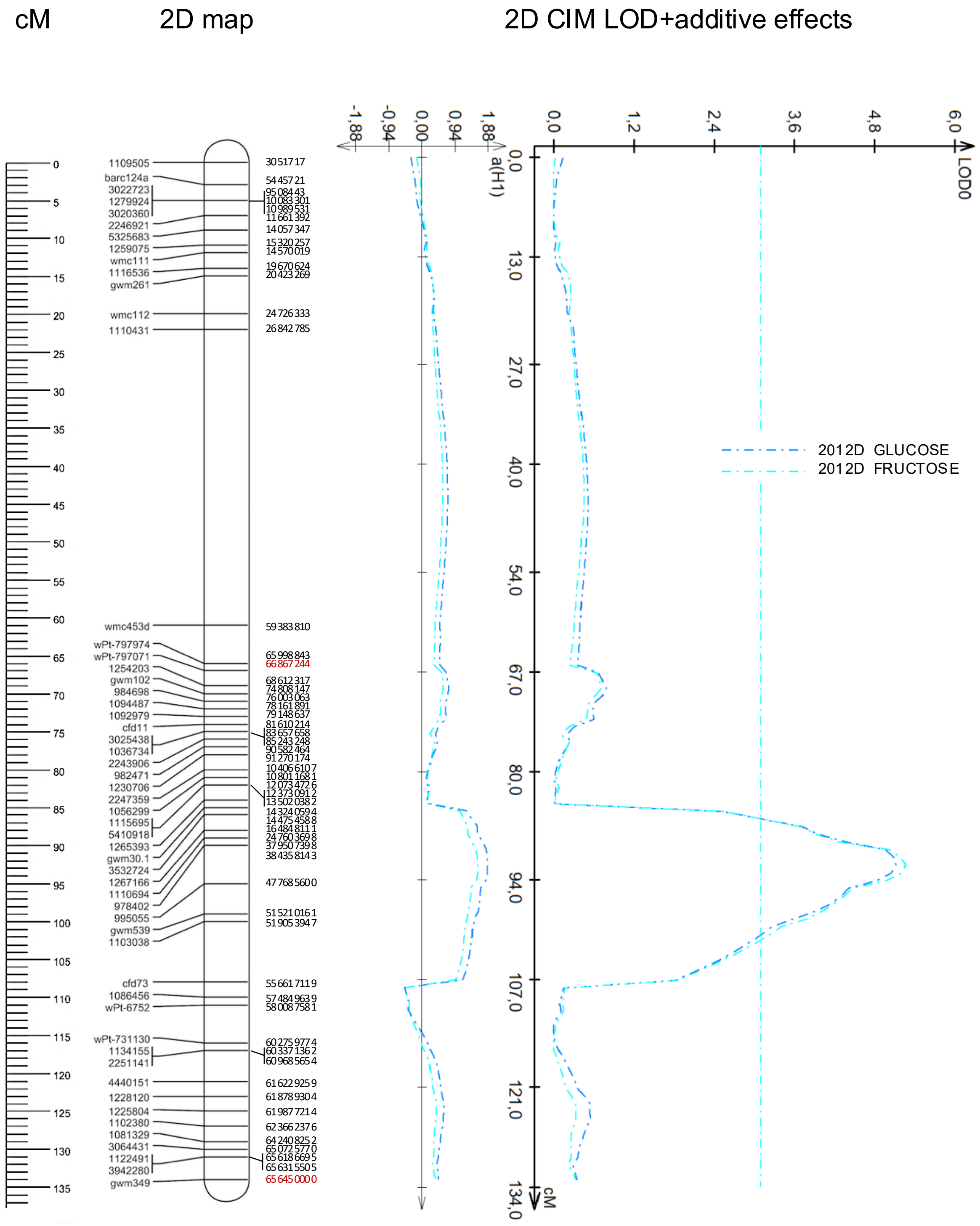
cM
2D map
2D CIM LOD+additive effects
a(H1)
LOD0
cM
2012D GLUCOSE
2012D FRUCTOSE

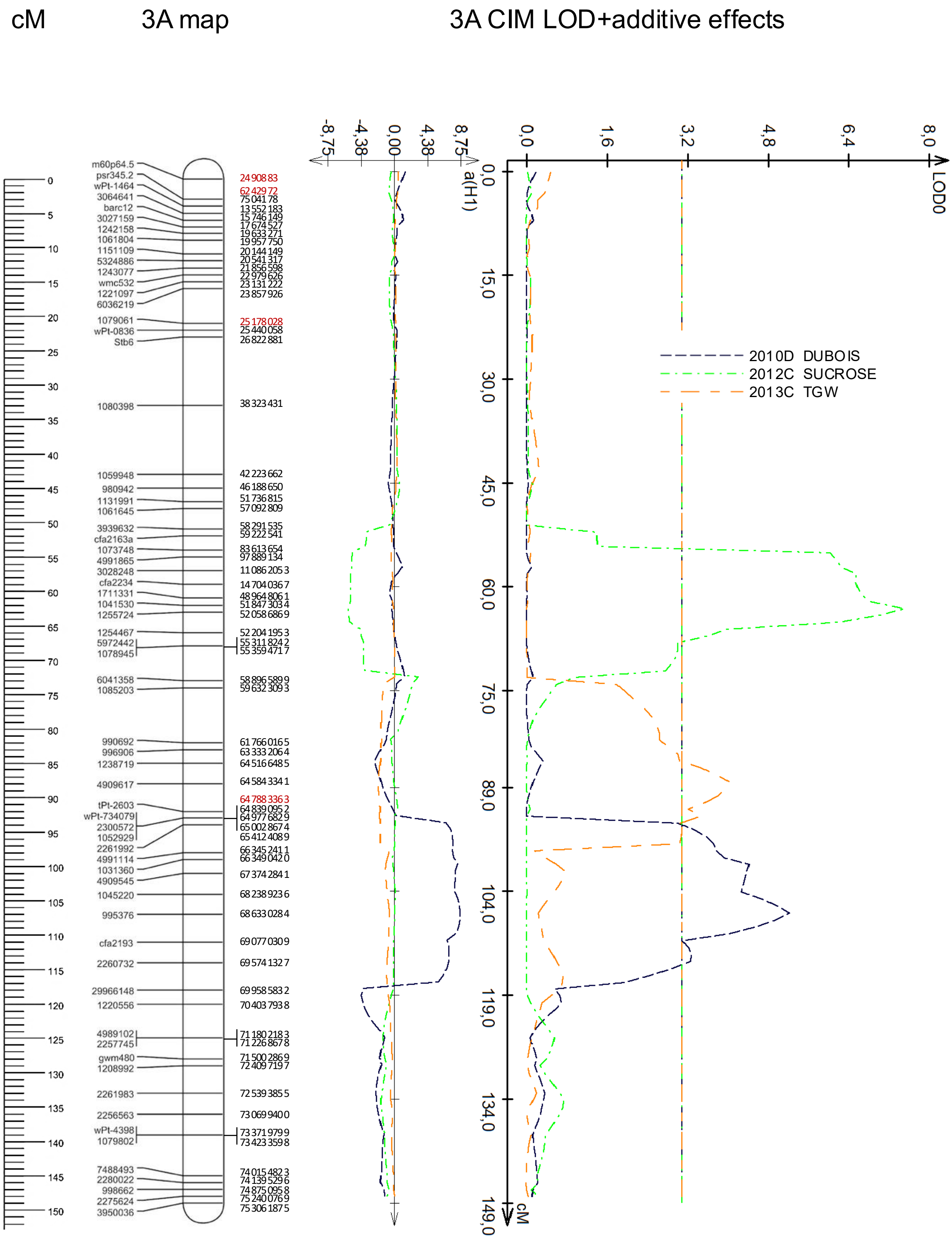
cM
3A map
3A CIM LOD+additive effects
-8,75
-4,38
0,00
4,38
8,75
a(H1)
0,0
1,6
3,2
4,8
6,4
8,0
LOD0
0,0
15,0
30,0
45,0
60,0
75,0
89,0
104,0
119,0
134,0
149,0
cM
2010D DUBOIS
2012C SUCROSE
2013C TGW

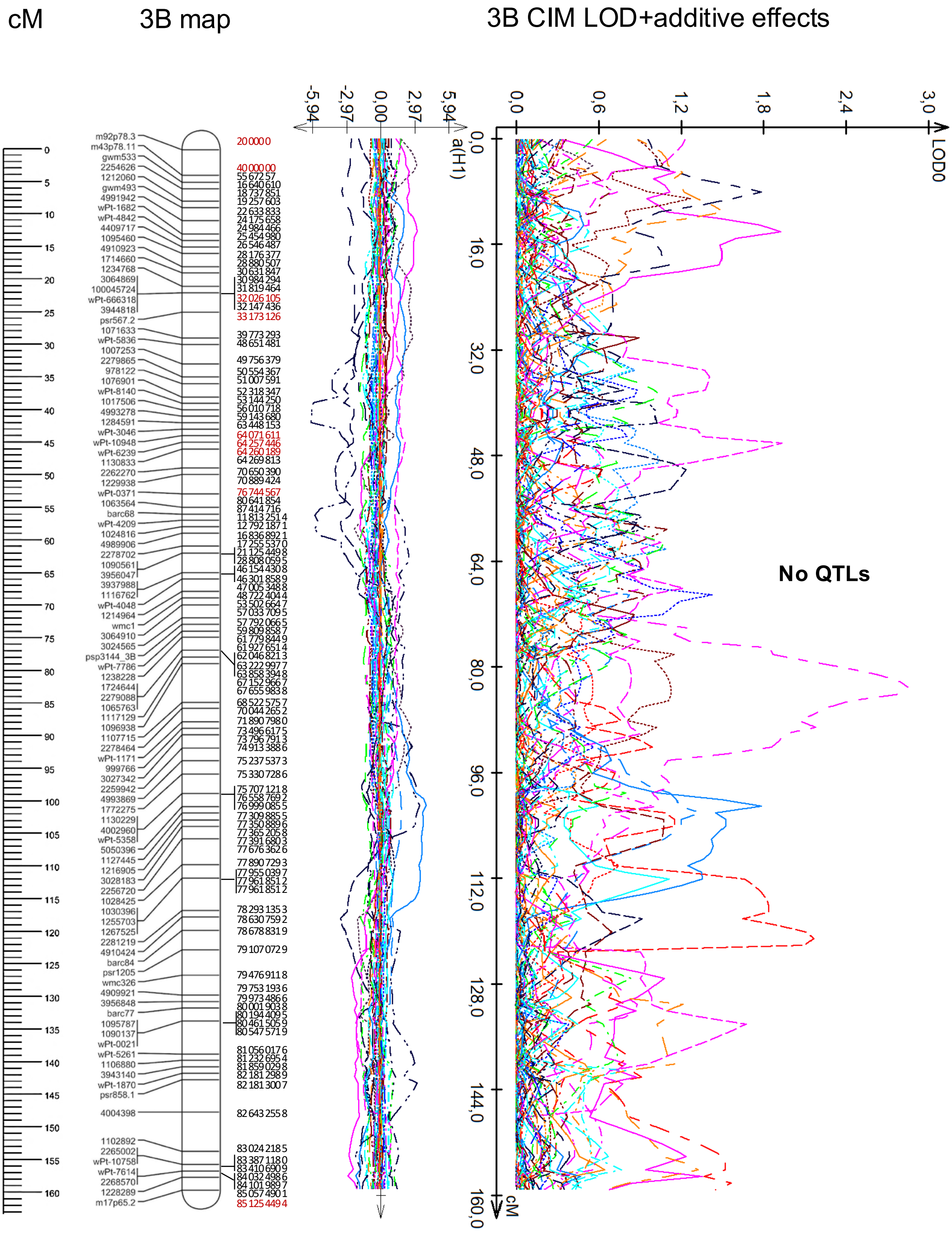
cM
3B map
3B CIM LOD+additive effects
a(H1)
LOD0
cM
No QTLs

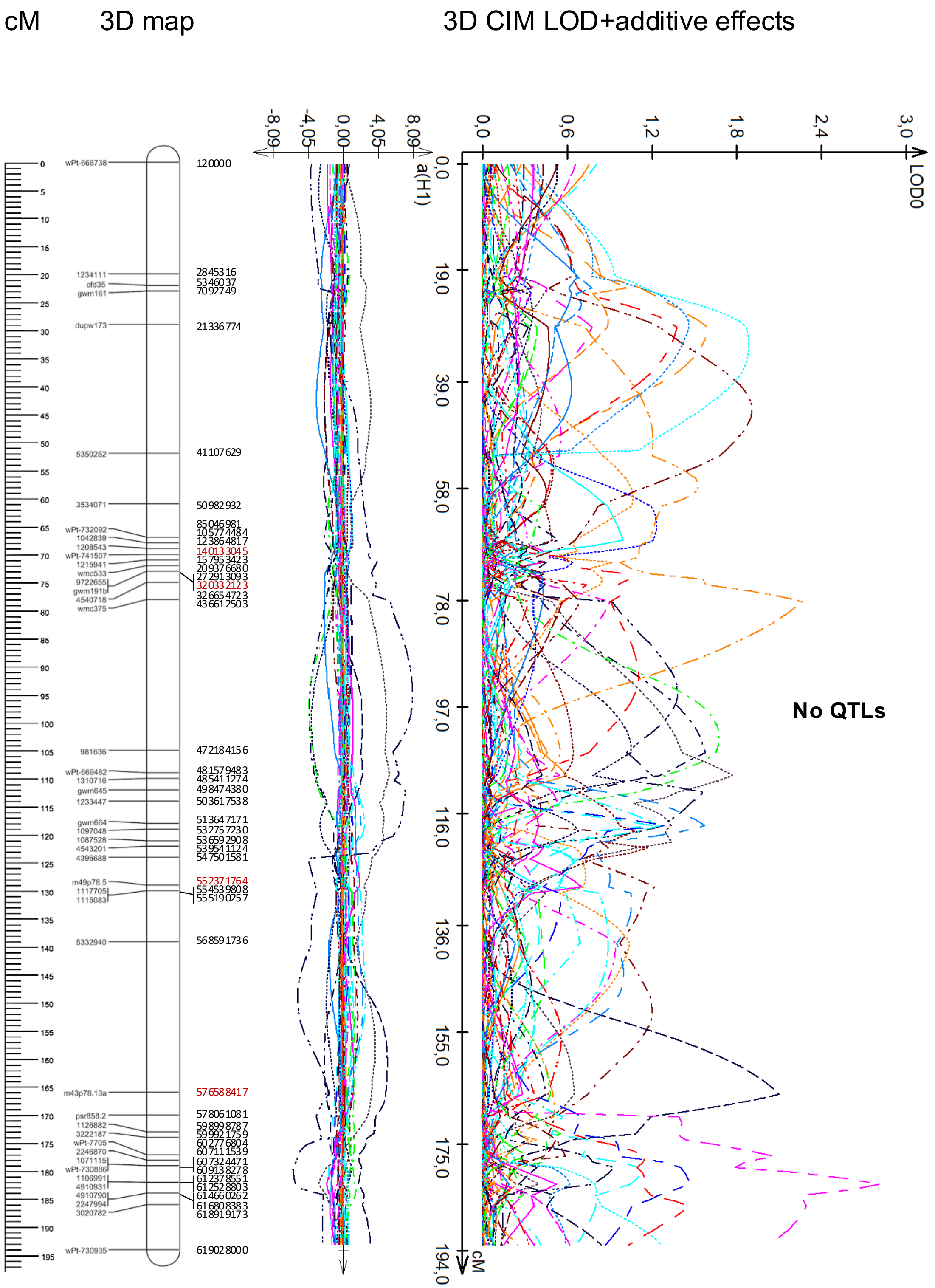
cM
3D map
3D CIM LOD+additive effects
a(H1)
LOD0
cM
No QTLs

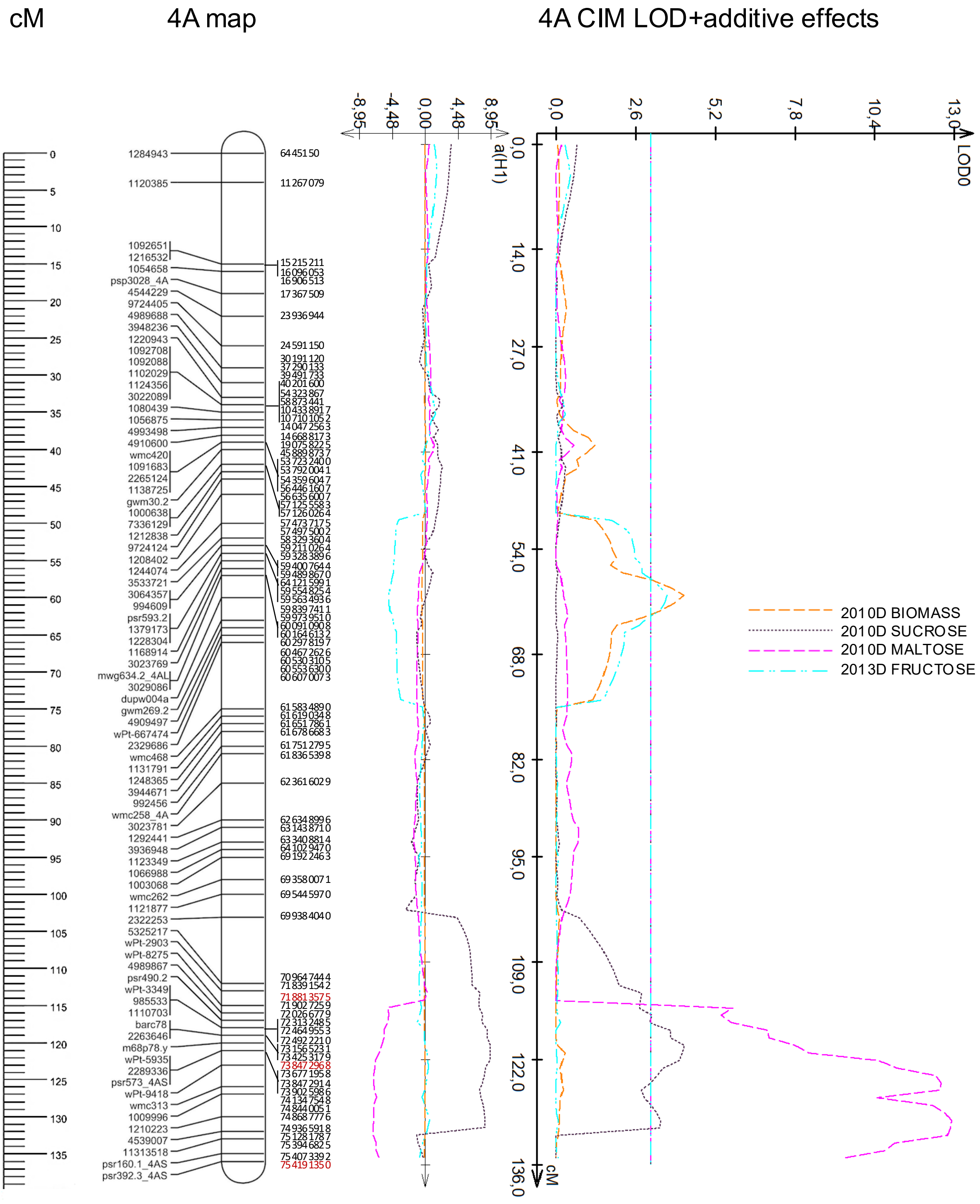
cM
4A map
4A CIM LOD+additive effects
-8,95 -4,48 0,00 4,48 8,95
a(H1)
0,0 2,6 5,2 7,8 10,4 13,0
LOD0
0,0 14,0 27,0 41,0 54,0 68,0 82,0 95,0 109,0 122,0 136,0
cM
2010D BIOMASS
2010D SUCROSE
2010D MALTOSE
2013D FRUCTOSE

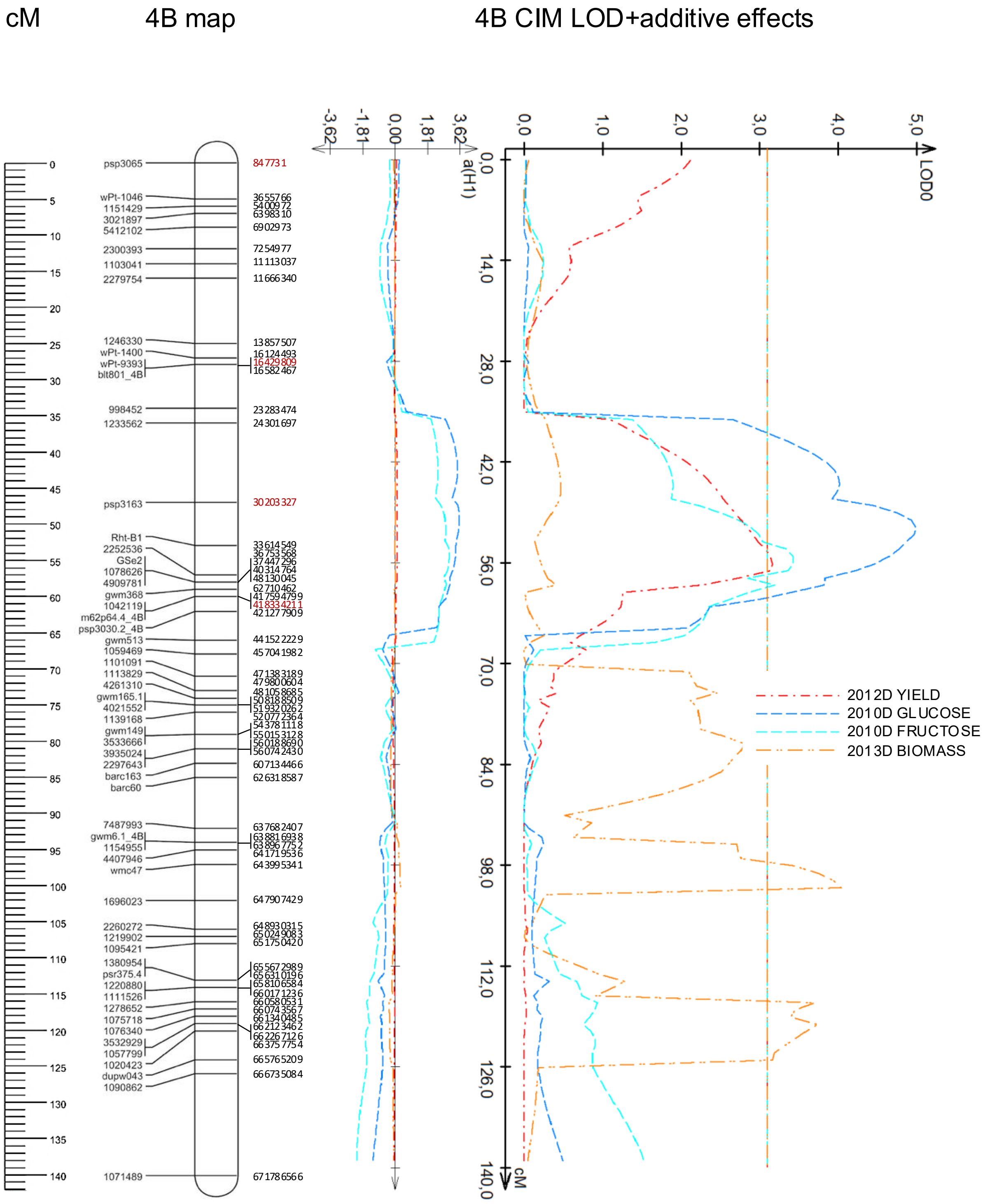

cM
4B map
4B CIM LOD+additive effects
psp3065
wPt-1046
1151429
3021897
5412102
2300393
1103041
2279754
1246330
wPt-1400
wPt-9393
blt801_4B
998452
1233562
psp3163
Rht-B1
2252536
GSe2
1078626
4909781
gwm368
1042119
m62p64.4_4B
psp3030.2_4B
gwm513
1059469
1101091
1113829
4261310
gwm165.1
4021552
1139168
gwm149
3533666
3935024
2297643
barc163
barc60
7487993
gwm6.1_4B
1154955
4407946
wmc47
1696023
2260272
1219902
1095421
1380954
psr375.4
1220880
1111526
1278652
1075718
1076340
3532929
1057799
1020423
dupw043
1090862
1071489
a(H1)
LOD0
2012D YIELD
2010D GLUCOSE
2010D FRUCTOSE
2013D BIOMASS

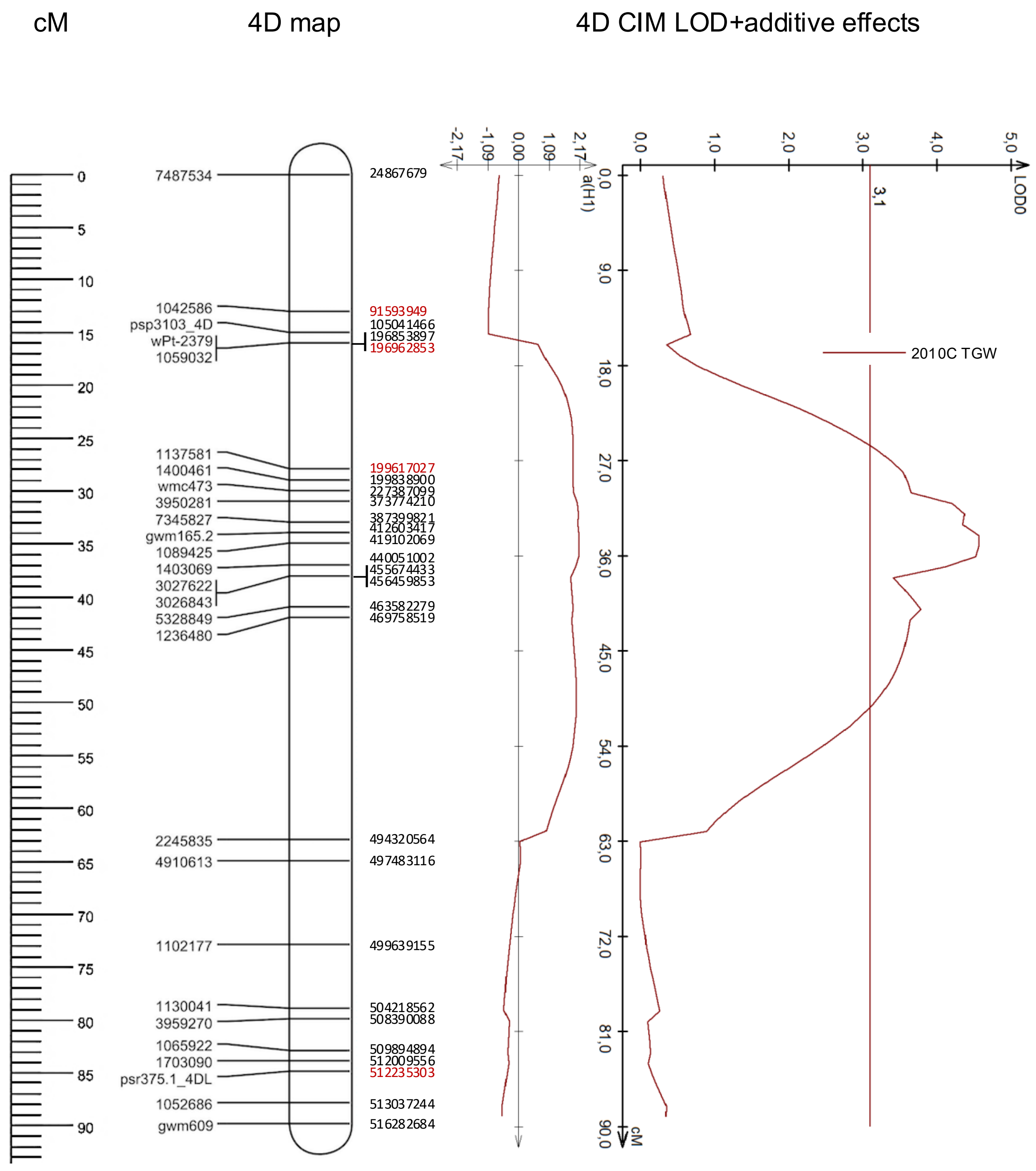
cM
4D map
4D CIM LOD+additive effects
0
5
10
15
20
25
30
35
40
45
50
55
60
65
70
75
80
85
90
7487534
1042586
psp3103_4D
wPt-2379
1059032
1137581
1400461
wmc473
3950281
7345827
gwm165.2
1089425
1403069
3027622
3026843
5328849
1236480
2245835
4910613
1102177
1130041
3959270
1065922
1703090
psr375.1_4DL
1052686
gwm609
24867679
91593949
105041466
196853897
196962853
199617027
199838900
227387099
373774210
387399821
412603417
419102069
440051002
455674433
456459853
463582279
469758519
494320564
497483116
499639155
504218562
508390088
509894894
512009556
512235303
513037244
516282684
-2,17
-1,09
0,00
1,09
2,17
a(H1)
0,0
9,0
18,0
27,0
36,0
45,0
54,0
63,0
72,0
81,0
90,0
cM
1,0
2,0
3,0
4,0
5,0
LOD0
3,1
2010C TGW

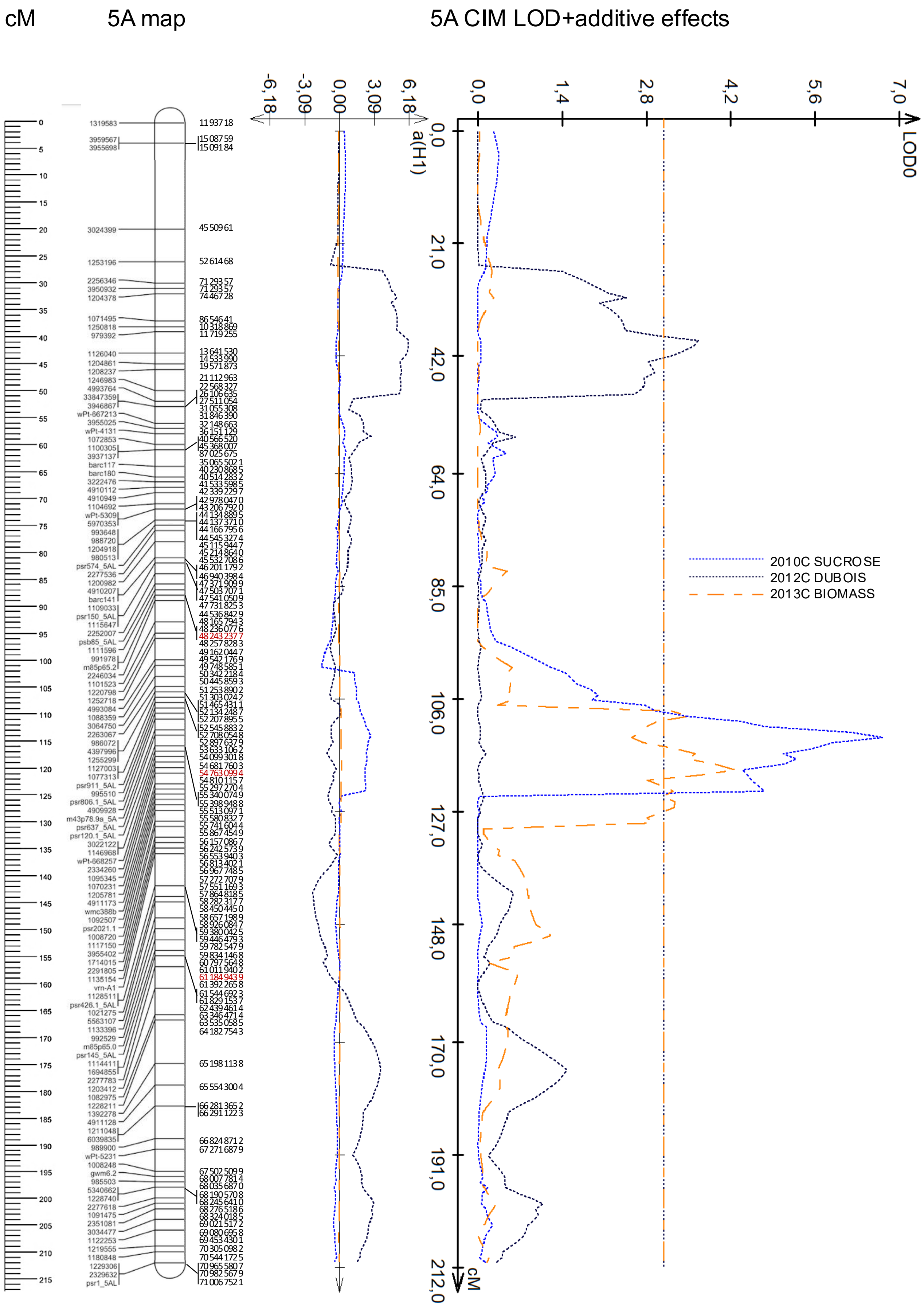

cM
5A map
5A CIM LOD+additive effects
a(H1)
-6,18
-3,09
0,00
3,09
6,18
LOD0
0,0
1,4
2,8
4,2
5,6
7,0
21,0
42,0
64,0
85,0
106,0
127,0
148,0
170,0
191,0
212,0
cM
2010C SUCROSE
2012C DUBOIS
2013C BIOMASS

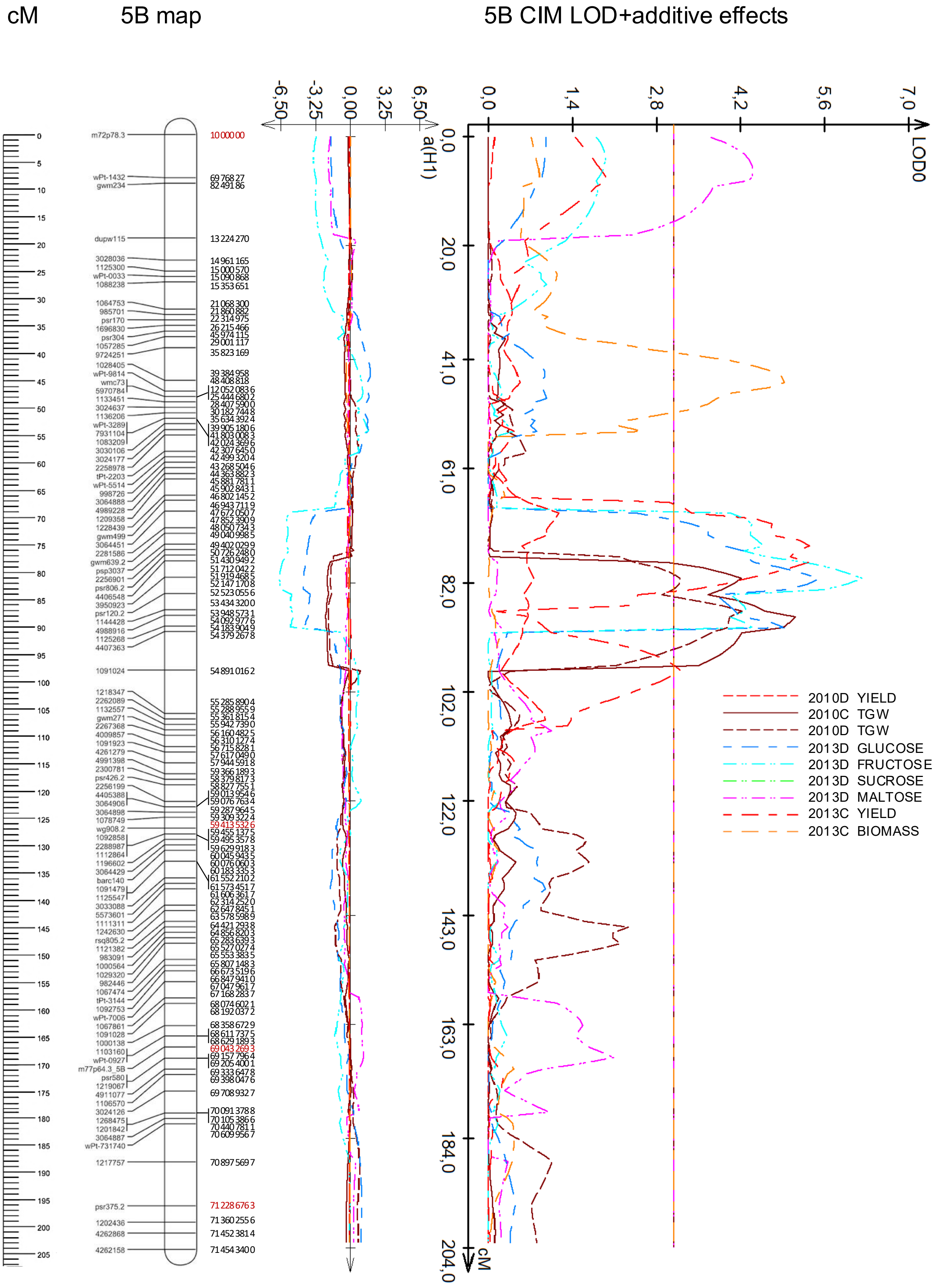

cM
5B map
5B CIM LOD+additive effects
a(H1)
LOD0
cM
2010D YIELD
2010C TGW
2010D TGW
2013D GLUCOSE
2013D FRUCTOSE
2013D SUCROSE
2013D MALTOSE
2013C YIELD
2013C BIOMASS

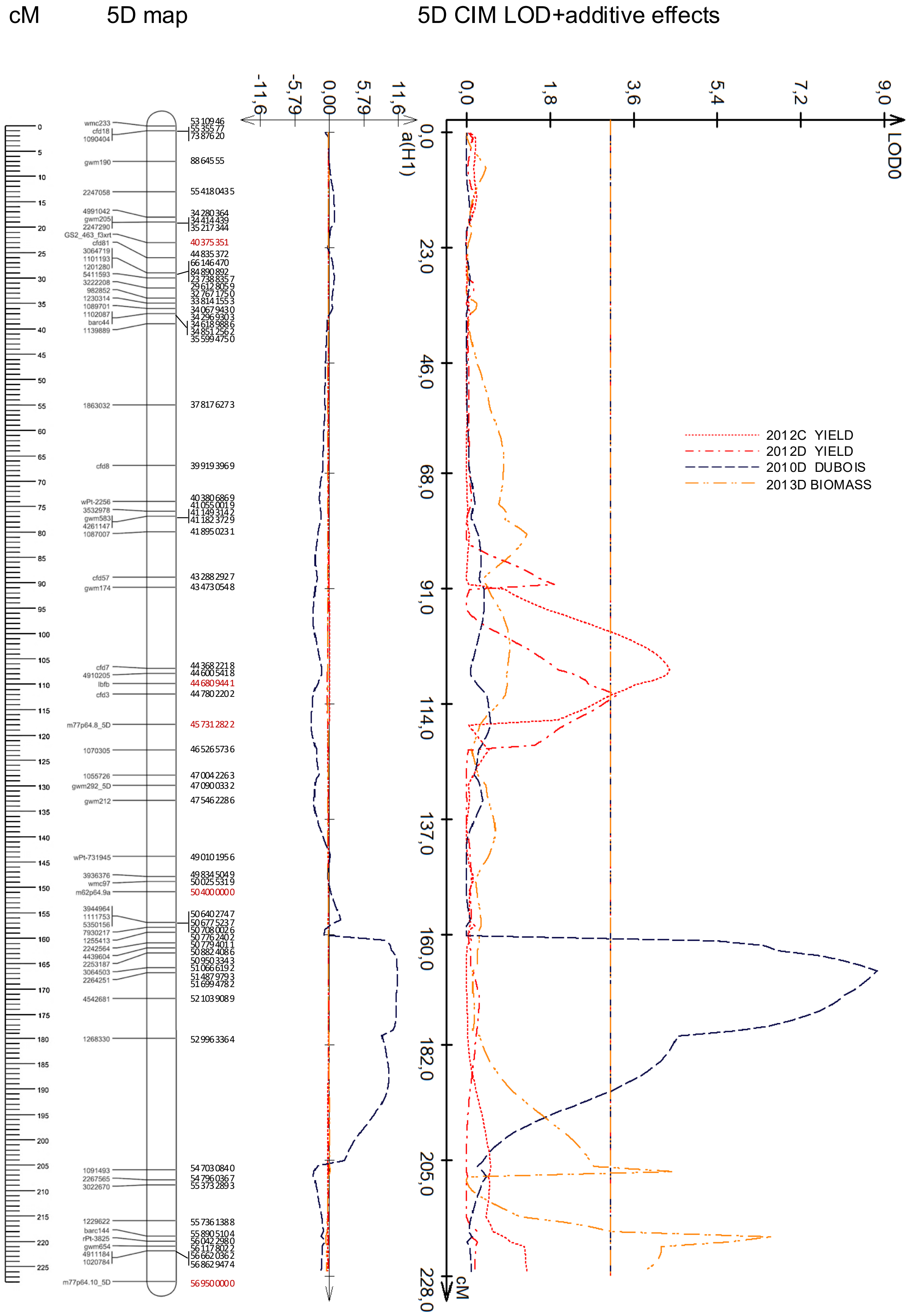

cM
5D map
5D CIM LOD+additive effects
-11,6
-5,79
0,00
5,79
11,6
a(H1)
0,0
1,8
3,6
5,4
7,2
9,0
LOD0
0,0
23,0
46,0
68,0
91,0
114,0
137,0
160,0
182,0
205,0
228,0
cM
2012C YIELD
2012D YIELD
2010D DUBOIS
2013D BIOMASS

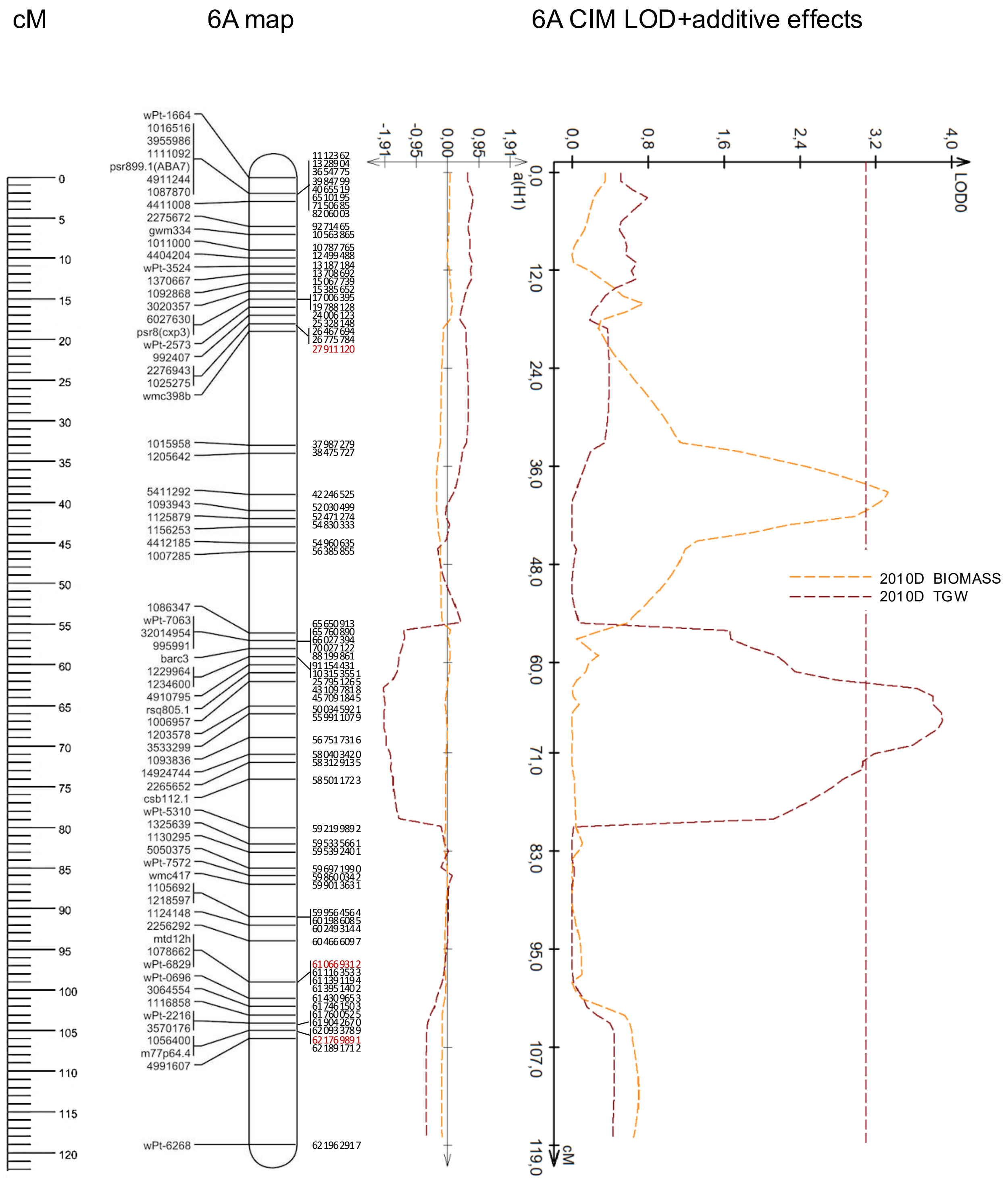
cM
6A map
6A CIM LOD+additive effects
wPt-1664
1016516
3955986
1111092
psr899.1(ABA7)
4911244
1087870
4411008
2275672
gwm334
1011000
4404204
wPt-3524
1370667
1092868
3020357
6027630
psr8(cxp3)
wPt-2573
992407
2276943
1025275
wmc398b
1015958
1205642
5411292
1093943
1125879
1156253
4412185
1007285
1086347
wPt-7063
32014954
995991
barc3
1229964
1234600
4910795
rsq805.1
1006957
1203578
3533299
1093836
14924744
2265652
csb112.1
wPt-5310
1325639
1130295
5050375
wPt-7572
wmc417
1105692
1218597
1124148
2256292
mtd12h
1078662
wPt-6829
wPt-0696
3064554
1116858
wPt-2216
3570176
1056400
m77p64.4
4991607
wPt-6268
a(H1)
LOD0
cM
2010D BIOMASS
2010D TGW

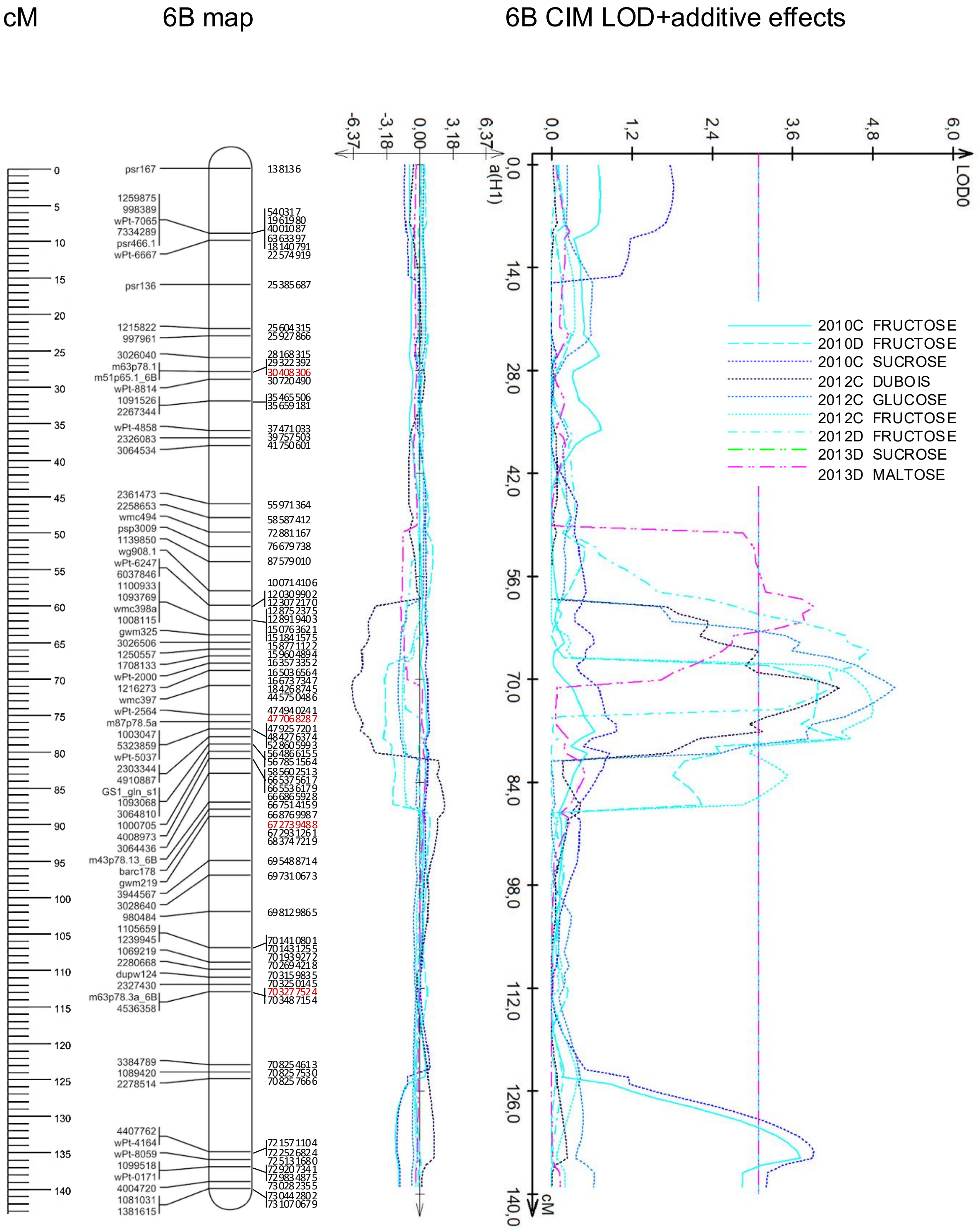
cM
6B map
6B CIM LOD+additive effects
a(H1)
-6,37
-3,18
0,00
3,18
6,37
LOD0
0,0
1,2
2,4
3,6
4,8
6,0
cM
2010C FRUCTOSE
2010D FRUCTOSE
2010C SUCROSE
2012C DUBOIS
2012C GLUCOSE
2012C FRUCTOSE
2012D FRUCTOSE
2013D SUCROSE
2013D MALTOSE

cM 6D map 6D CIM LOD+additive effects

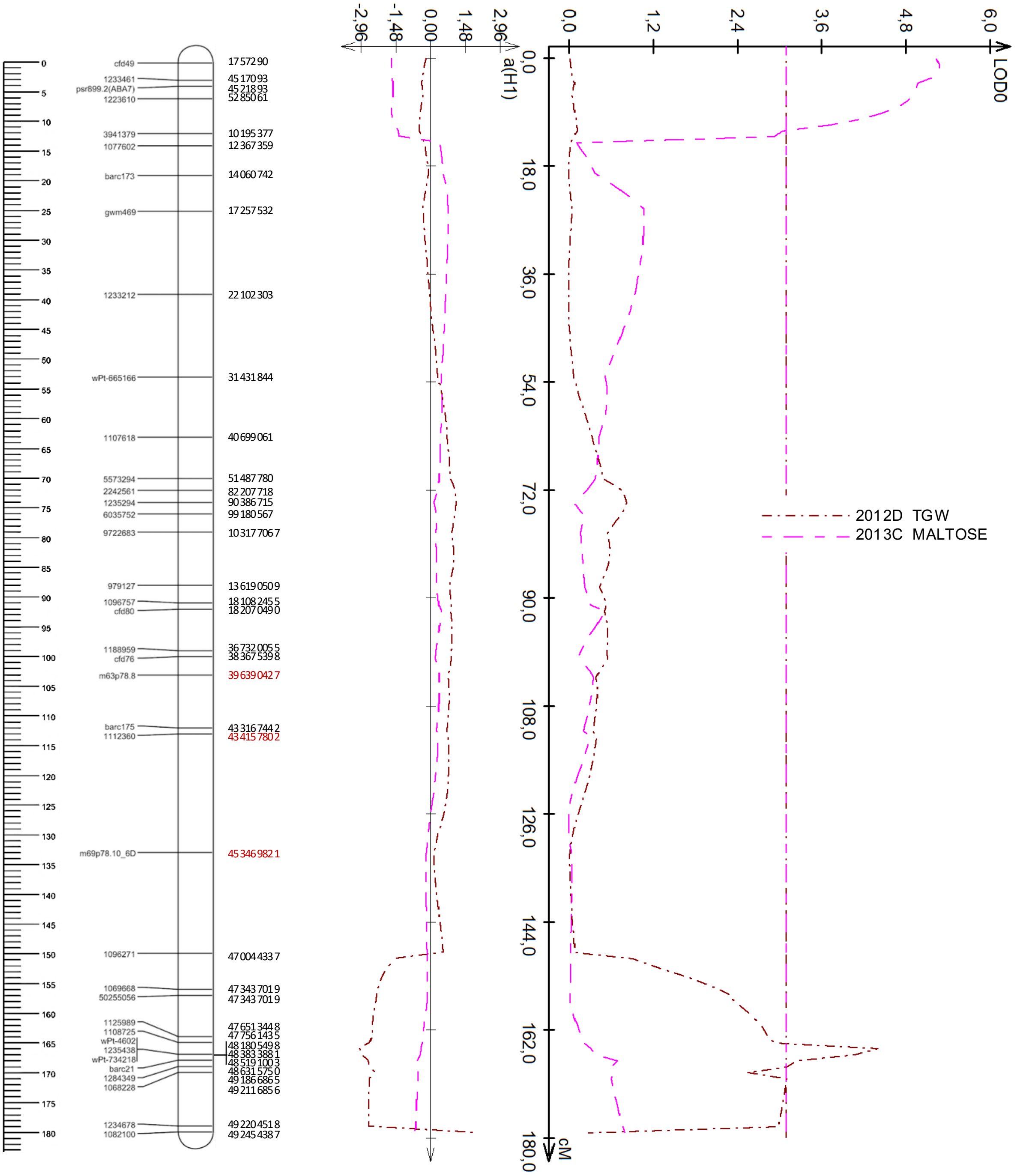

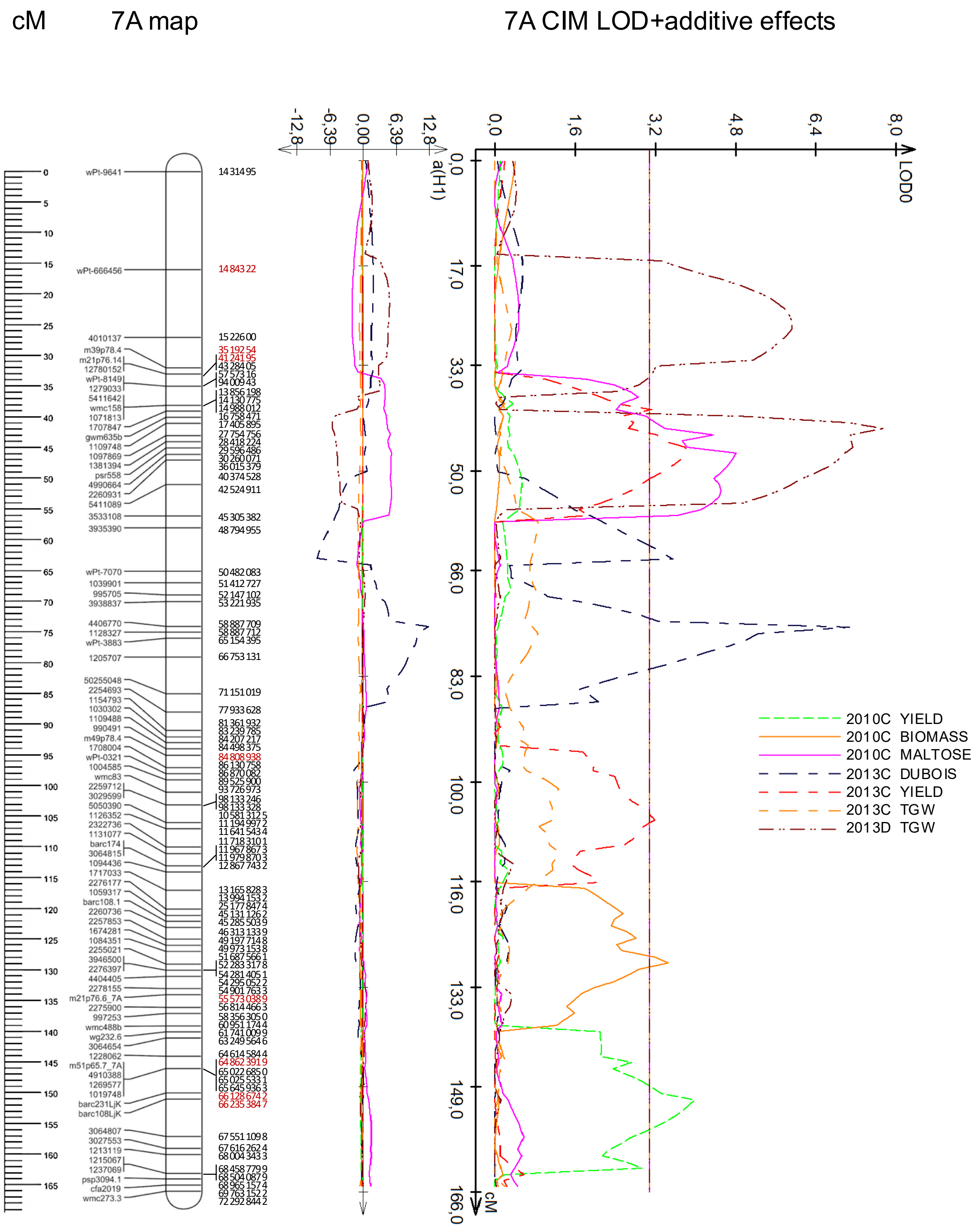
cM
7A map
7A CIM LOD+additive effects
a(H1)
LOD0
cM
2010C YIELD
2010C BIOMASS
2010C MALTOSE
2013C DUBOIS
2013C YIELD
2013C TGW
2013D TGW

cM 7B map

7B CIM LOD+additive effects

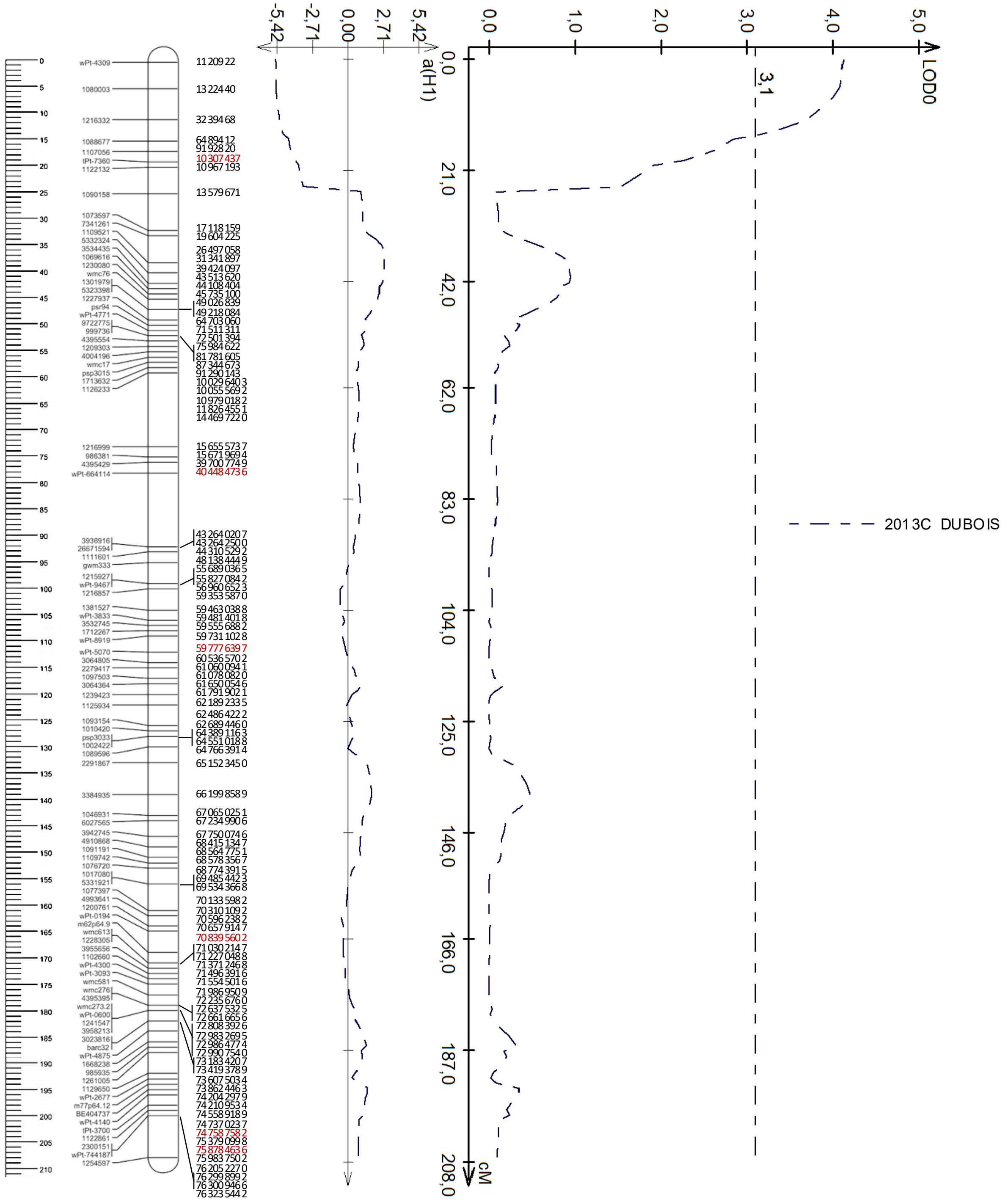

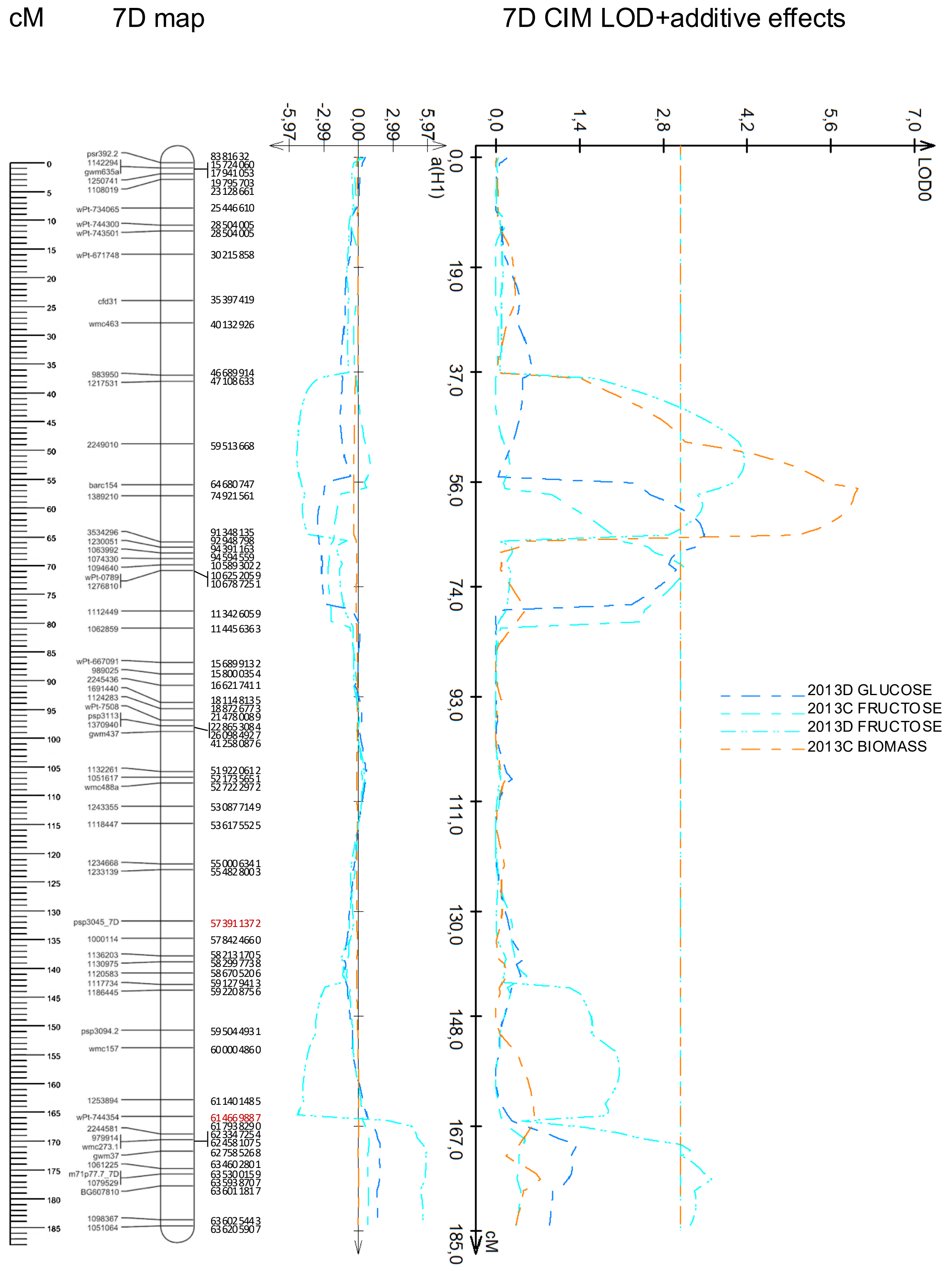
cM
7D map
7D CIM LOD+additive effects
a(H1)
LOD0
cM
2013D GLUCOSE
2013C FRUCTOSE
2013D FRUCTOSE
2013C BIOMASS
