# Supplementary material for: Impact of Soil Drought on Yield and Leaf Sugar Content in Wheat: Genotypic and Phenotypic Relationships Compared Using a Doubled Haploid Population
Source: Int J Mol Sci. 2025 Aug 13;26(16):7833. doi: 10.3390/ijms26167833 (PMC12386748; doi:10.3390/ijms26167833)
Supplement: Supplementary file 1 [file ijms-26-07833-s001.zip › ijms-3707475-supplementary_R1(TS1-TS8; FIG.S1-FIG.pdf]

Table S1. Average yield (in g), for genotypes and environments, the principal component analysis values, the AMMI stability value (ASV), rank of the AMMI stability value (RA), rank of trait mean (RM), and genotype selection index (GSI) of tested DH lines and their parental components (CS and SQ1)

Line	2010,C	2010,D	2012,C	2012,D	2013,C	2013,D	Mean	IPCAg1	IPCAg2	ASV	RA	RM	GSI
1	2.629	1.377	3.386	0.617	1.281	0.521	1.635	0.311	-0.025	0.436	68	55	123
2	3.288	2.377	2.831	1.577	1.970	0.591	2.106	-0.166	0.152	0.277	36	7	43
3	3.045	0.495	3.323	0.489	1.601	0.728	1.613	0.206	-0.250	0.382	60	61	121
4	2.855	1.857	3.346	0.914	1.781	0.434	1.864	0.196	0.038	0.276	34	24	58
5	3.49	1.271	3.288	0.560	1.562	0.719	1.815	0.110	-0.246	0.290	38	31	69
7	3.664	1.355	2.233	0.456	1.676	0.804	1.698	-0.279	-0.078	0.398	64	49	113
8	2.646	0.899	4.328	0.629	1.386	0.818	1.785	0.630	-0.219	0.907	91	34	125
9	2.541	1.703	3.178	0.454	1.704	0.835	1.736	0.275	0.197	0.431	67	42	109
10	1.422	1.451	2.215	0.603	1.694	0.860	1.374	0.129	0.691	0.714	88	85	173
11	2.499	1.362	2.715	0.654	1.428	0.403	1.510	0.088	0.125	0.175	16	75	91
13	3.051	1.805	3.298	0.990	1.829	0.673	1.941	0.131	0.025	0.185	19	15	34
14	2.114	1.838	3.306	0.722	1.722	0.933	1.772	0.365	0.367	0.628	84	38	122
15	2.780	1.386	2.608	1.674	1.600	1.021	1.853	-0.153	0.228	0.313	45	25	70
17	2.824	1.372	2.312	0.760	1.755	0.617	1.607	-0.140	0.166	0.257	29	65	93.5
18	3.366	1.159	3.945	0.854	1.821	0.397	1.923	0.303	-0.371	0.563	77	17	94
19	3.142	1.340	3.122	1.628	1.367	0.621	1.870	-0.036	-0.097	0.109	8	23	31
20	3.483	1.932	2.318	1.481	1.818	0.981	2.002	-0.363	0.152	0.529	76	11	87
21	2.951	1.438	2.998	0.811	1.464	0.823	1.747	0.083	0.030	0.120	11	41	52
22	3.118	1.511	3.606	0.533	1.647	1.046	1.910	0.304	-0.066	0.429	66	19	85
23	2.215	1.374	3.069	1.281	1.594	1.019	1.759	0.177	0.309	0.396	63	40	103
24	3.155	1.573	4.161	0.843	2.081	1.357	2.195	0.434	-0.055	0.609	83	4	87
25	3.693	1.268	3.764	1.549	1.860	1.041	2.196	0.082	-0.279	0.302	40	3	43
26	4.437	1.974	3.900	3.372	2.041	0.662	2.731	-0.293	-0.416	0.583	79	1	80
27	2.624	1.568	3.405	0.516	1.069	0.716	1.650	0.348	0.024	0.487	73	51	124
28	2.648	0.996	1.533	1.244	1.765	0.786	1.495	-0.456	0.336	0.720	89	77	166
29	2.442	1.849	2.390	0.830	1.133	0.325	1.495	-0.025	0.255	0.257	30	77	107

30	2.238	1.602	2.610	0.508	1.053	0.697	1.451	0.146	0.291	0.356	54	79	133
31	3.687	1.611	2.586	0.445	1.517	0.358	1.701	-0.157	-0.199	0.296	39	48	87
32	3.584	1.149	2.667	0.728	1.667	0.550	1.724	-0.160	-0.208	0.306	41	46	87
33	2.843	1.914	3.072	0.333	1.502	0.697	1.727	0.206	0.108	0.307	43	45	88
34	2.665	1.026	2.410	0.480	1.201	0.559	1.390	-0.018	0.064	0.069	3	83	86
35	3.523	1.431	2.055	0.674	0.870	0.697	1.542	-0.318	-0.076	0.451	69	73	142
36	2.524	1.264	2.598	0.201	0.871	0.297	1.292	0.128	0.034	0.182	18	90	108
37	2.330	1.308	2.812	0.567	0.855	0.701	1.429	0.194	0.154	0.311	44	81	125
38	2.604	1.361	2.447	0.399	1.256	0.153	1.370	0.014	0.066	0.070	4	86	90
39	3.319	1.620	2.867	0.649	1.596	1.363	1.902	-0.003	0.078	0.078	6	20	26
40	2.610	1.754	2.026	0.479	1.186	0.662	1.453	-0.129	0.306	0.355	53	78	131
41	2.680	1.203	1.630	0.381	1.457	0.602	1.326	-0.286	0.259	0.476	71	87	158
42	2.737	0.852	2.255	0.844	1.731	0.848	1.545	-0.159	0.150	0.268	31	72	103
43	3.939	1.517	3.642	0.951	1.389	0.560	2.000	0.095	-0.444	0.463	70	13	82.5
45	2.259	1.107	2.931	1.050	1.528	0.593	1.578	0.146	0.174	0.268	32	70	102
46	2.925	1.191	3.500	0.748	1.467	0.807	1.773	0.269	-0.101	0.389	62	36	98
47	2.986	1.222	2.521	0.522	1.412	0.728	1.565	-0.051	0.030	0.077	5	71	76
48	3.546	1.301	2.773	0.592	1.494	0.972	1.780	-0.079	-0.125	0.167	15	35	50
49	2.822	1.079	1.519	0.380	1.649	1.122	1.429	-0.353	0.326	0.591	80	805	885
50	2.441	1.179	2.438	0.409	1.123	0.942	1.422	0.057	0.225	0.239	26	82	108
51	3.947	1.188	2.949	0.411	1.703	1.349	1.925	-0.071	-0.222	0.243	27	16	43
52	2.804	1.684	3.393	0.914	1.679	0.916	1.898	0.232	0.096	0.337	49	21	70
53	3.958	1.010	2.687	1.398	1.477	0.420	1.825	-0.324	-0.384	0.593	81	30	111
54	3.012	1.230	2.892	0.766	1.634	0.738	1.712	0.030	-0.012	0.043	1	47	48
55	1.958	1.255	2.443	0.487	0.968	0.610	1.287	0.142	0.320	0.377	59	91	150
56	3.285	1.276	3.489	0.698	1.613	0.718	1.847	0.197	-0.205	0.344	51	26	77
57	2.838	1.871	2.813	0.974	1.654	1.523	1.946	0.027	0.337	0.339	50	14	64
58	3.709	1.644	2.420	1.345	1.557	0.655	1.888	-0.351	-0.085	0.498	74	22	96
59	2.694	1.205	2.551	0.756	1.467	0.860	1.589	-0.019	0.154	0.157	13	69	82
60	2.854	1.679	2.134	0.716	1.606	0.718	1.618	-0.190	0.249	0.365	55	58	113

61	2.810	1.620	3.158	1.028	1.026	0.334	1.663	0.144	-0.046	0.206	22	50	72
62	3.259	1.442	3.376	0.838	1.022	0.697	1.772	0.166	-0.201	0.306	42	38	79.5
63	2.576	1.558	2.742	1.298	2.075	0.570	1.803	-0.033	0.243	0.247	28	32	60
64	2.709	1.489	3.089	1.079	1.517	0.921	1.800	0.122	0.134	0.216	24	33	57
65	3.320	1.977	2.773	1.838	1.501	0.817	2.038	-0.217	0.083	0.314	46	9	55
66	3.679	1.600	4.413	0.660	1.531	0.324	2.034	0.452	-0.519	0.817	90	10	100
67	3.347	1.071	2.680	0.648	1.529	0.507	1.630	-0.094	-0.172	0.216	23	56	79
69	2.599	0.963	3.102	1.970	1.592	0.723	1.825	-0.001	0.063	0.063	2	30	31.5
70	3.249	2.056	2.704	0.933	1.349	0.663	1.826	-0.086	0.064	0.136	12	28	40
71	4.111	1.235	3.284	1.707	1.985	0.682	2.167	-0.202	-0.389	0.481	72	6	78
72	3.125	1.373	2.768	0.792	1.279	0.415	1.625	-0.030	-0.094	0.103	7	57	64
80	3.449	1.500	2.746	0.450	1.232	0.474	1.642	-0.044	-0.175	0.186	20	52	72
86	2.840	1.158	3.207	0.772	1.449	0.273	1.616	0.171	-0.131	0.272	33	59	92
87	3.159	1.499	3.019	0.820	0.948	0.927	1.729	0.070	-0.058	0.114	9	44	52.5
88	3.279	1.394	2.367	0.776	1.379	0.645	1.640	-0.197	-0.017	0.276	35	53	88
90	3.808	1.327	3.636	0.669	1.951	0.969	2.060	0.144	-0.313	0.372	57	8	65
91	3.462	1.653	2.771	0.684	1.584	0.823	1.829	-0.078	-0.048	0.119	10	27	37
92	1.991	1.674	2.483	0.500	0.858	0.325	1.305	0.153	0.318	0.383	61	88	149
93	2.218	1.487	3.592	0.529	1.216	0.635	1.613	0.483	0.107	0.682	86	61	147
94	2.808	0.662	2.645	0.365	0.900	0.107	1.248	0.051	-0.210	0.222	25	92	117
97	3.738	1.240	3.245	0.369	1.300	0.482	1.729	0.081	-0.401	0.416	65	44	109
98	4.242	1.467	2.934	2.357	2.134	0.609	2.291	-0.452	-0.308	0.703	87	2	89
99	3.389	1.417	2.512	0.440	1.368	0.493	1.603	-0.119	-0.114	0.201	21	67	88
102	3.716	1.241	2.090	0.835	1.527	0.260	1.611	-0.403	-0.198	0.596	82	63	145
104	2.585	1.712	2.499	0.381	1.673	0.747	1.599	0.039	0.282	0.287	37	68	105
105	3.670	1.585	2.519	0.896	1.234	0.684	1.765	-0.229	-0.138	0.349	52	39	91
114	3.391	1.822	3.193	0.455	0.718	0.240	1.636	0.143	-0.266	0.333	48	54	102
115	3.708	1.789	2.697	2.685	1.464	0.697	2.173	-0.450	-0.068	0.632	85	5	90
124	2.665	1.359	3.277	0.735	0.870	0.737	1.607	0.267	-0.015	0.373	58	65	123
127	2.192	1.344	2.715	0.447	0.857	0.763	1.386	0.206	0.231	0.369	56	84	140

128	3.020	1.593	2.902	0.336	1.165	0.606	1.604	0.117	-0.029	0.166	14	66	80
143	4.046	1.774	1.587	1.653	1.861	1.081	2.000	-0.757	0.095	1.062	92	13	105
144	2.936	0.473	1.655	0.844	1.308	0.588	1.301	-0.403	0.030	0.563	78	89	167
146	4.048	1.273	2.587	1.057	1.772	0.748	1.914	-0.327	-0.265	0.528	75	18	93
CS	3.382	1.626	2.051	0.251	1.161	0.728	1.533	-0.236	0.029	0.330	47	74	121
SQ1	2.800	1.665	2.680	0.299	1.550	0.673	1.611	0.076	0.143	0.178	17	63	79.5
Mean	3.052	1.429	2.841	0.850	1.464	0.697	1.722						
Variance	0.588	0.214	0.479	0.326	0.130	0.091	1.135						
IPCAe1	-1.048	0.080	1.892	-0.817	-0.200	0.092							
IPCAe2	-1.490	0.863	-0.803	0.130	0.427	0.871							

IPCAg1, IPCAg2, principal components of interaction for genotypes; IPCAe1, IPCAe2, principal components of interaction for environments.

Table S2. Average biomass (in g), for genotypes and environments, the principal component analysis values, the AMMI stability value (ASV), rank of the AMMI stability value (RA), rank of trait mean (RM), and genotype selection index (GSI) of tested DH lines and their parental components (CS and SQ1)

Line	2010,C	2010,D	2012,C	2012,D	2013,C	2013,D	Mean	IPCAg1	IPCAg2	ASV	RA	RM	GSI
1	5.049	2.839	6.074	1.841	2.398	1.451	3.28	0.361	-0.148	0.469	1	50	118
2	6.792	5.181	5.802	3.548	4.108	1.608	4.51	-0.198	0.182	0.305	2	18	34
3	6.385	2.174	6.446	2.009	2.867	1.742	3.60	0.096	-0.418	0.434	3	59	90
4	5.976	3.877	6.673	2.244	3.280	1.172	3.87	0.190	-0.293	0.375	4	84	67
5	6.268	2.907	5.637	1.93	2.882	1.743	3.56	-0.027	-0.100	0.106	5	42	47
7	7.111	3.228	4.634	1.909	3.481	1.847	3.70	-0.435	0.145	0.556	6	12	107
8	5.781	2.444	6.793	1.643	2.485	1.604	3.46	0.324	-0.464	0.613	7	57	129
9	4.736	3.246	5.248	1.492	3.153	1.855	3.29	0.353	0.251	0.502	8	62	126
10	3.565	1.926	4.726	0.932	3.156	1.925	2.71	0.604	0.366	0.831	9	22	177
11	5.071	2.962	5.702	2.490	3.032	1.356	3.44	0.258	0.013	0.318	10	37	81
13	6.045	3.791	6.292	2.426	3.620	1.426	3.93	0.121	-0.138	0.204	11	79	36
14	5.603	4.269	6.910	2.420	3.882	2.464	4.26	0.477	0.005	0.588	12	24	88
15	5.346	2.964	5.131	3.769	3.056	2.296	3.76	0.121	0.375	0.403	13	27	74
17	5.347	3.012	4.648	2.338	2.710	1.414	3.25	-0.017	0.256	0.257	14	76	80
18	6.180	2.464	6.453	2.063	3.150	1.031	3.56	0.082	-0.470	0.481	15	23	105
19	6.689	3.329	6.290	3.823	2.720	1.963	4.14	-0.067	-0.140	0.163	16	64	18
20	6.911	4.276	5.130	3.398	3.804	1.951	4.25	-0.324	0.280	0.488	17	54	74
21	5.902	2.998	5.249	2.033	2.787	1.683	3.44	-0.013	0.054	0.056	18	67	51
22	6.082	3.412	6.509	1.729	2.974	2.224	3.82	0.254	-0.174	0.358	19	65	66
23	4.488	2.920	5.370	2.580	2.939	2.303	3.43	0.448	0.343	0.651	20	92	134
24	5.768	2.963	7.535	1.962	3.859	2.637	4.12	0.589	-0.338	0.801	21	74	103
25	7.487	3.252	7.000	3.674	4.250	2.532	4.70	-0.060	-0.283	0.293	22	82	26
26	8.814	4.385	7.941	6.110	4.495	2.215	5.66	-0.366	-0.480	0.659	23	3	85
27	5.013	3.282	5.651	1.600	2.104	1.427	3.18	0.290	-0.008	0.358	24	89	104
28	5.605	3.497	5.848	3.418	3.494	2.147	4.00	0.193	0.184	0.301	25	81	43
29	4.843	3.319	4.323	1.586	2.362	0.844	2.88	0.022	0.285	0.287	26	17	104

30	4.213	3.134	4.671	1.491	2.040	0.340	2.65	0.210	0.141	0.295	27	8	113
31	7.378	4.029	6.256	1.710	3.128	1.304	3.97	-0.248	-0.357	0.470	28	6	79
32	6.944	3.098	5.101	2.290	3.140	1.548	3.69	-0.349	-0.022	0.431	29	39	83
33	6.433	4.359	5.712	1.855	2.708	0.498	3.59	-0.189	-0.203	0.309	30	51	68
34	5.014	2.252	3.797	1.298	2.024	1.249	2.61	-0.079	0.319	0.334	31	46	123
35	6.687	2.889	4.092	2.260	2.520	0.498	3.16	-0.593	0.042	0.732	32	91	157
36	5.109	2.745	4.812	1.416	1.894	0.930	2.82	0.052	0.035	0.073	33	36	88
37	4.379	2.800	4.717	1.499	1.524	1.498	2.74	0.286	0.251	0.433	34	58	136
38	4.250	2.257	4.060	1.410	2.041	0.617	2.44	0.113	0.242	0.279	35	73	112
39	6.377	3.537	5.433	2.048	3.018	2.741	3.86	0.005	0.207	0.207	36	47	40
40	4.609	2.970	3.536	1.418	1.969	1.349	2.64	-0.014	0.550	0.550	37	29	162
41	5.306	2.727	4.407	1.446	2.685	1.638	3.04	0.004	0.280	0.280	38	66	95
42	5.155	2.231	4.822	0.836	2.995	1.802	2.97	0.172	0.137	0.252	39	83	90
43	6.785	3.139	5.849	2.100	2.284	1.164	3.55	-0.205	-0.317	0.405	40	25	92
45	4.975	2.912	6.579	2.879	3.150	1.499	3.67	0.458	-0.164	0.588	41	26	113
46	6.681	2.863	7.130	2.303	3.093	1.747	3.97	0.140	-0.531	0.559	42	15	96
47	5.162	2.467	4.212	1.514	2.387	1.554	2.88	-0.010	0.296	0.296	43	88	106
48	7.104	2.940	5.408	1.672	2.818	2.049	3.67	-0.262	-0.105	0.340	44	30	69
49	5.260	2.419	3.737	1.487	2.862	2.060	2.97	-0.069	0.516	0.523	46	31	146
50	5.024	2.643	4.204	1.454	2.035	1.807	2.86	0.050	0.363	0.368	46	72	122
51	7.361	3.152	5.750	1.447	3.286	2.630	3.94	-0.190	-0.094	0.253	47	70	38
52	6.133	3.858	6.281	2.452	3.042	1.969	3.96	0.142	-0.066	0.187	48	44	31
53	6.883	2.590	5.176	2.505	2.785	1.115	3.51	-0.375	-0.176	0.495	49	40	110
54	6.453	3.258	7.164	2.603	4.008	1.795	4.21	0.217	-0.397	0.479	50	33	72
55	4.481	2.968	5.020	1.771	1.831	1.493	2.93	0.311	0.204	0.435	51	85	131
56	6.908	3.380	6.870	2.012	3.130	1.542	3.97	0.016	-0.480	0.481	52	38	82
57	5.566	3.866	5.106	2.357	3.258	2.807	3.83	0.164	0.480	0.521	53	78	96
58	7.143	4.132	5.121	3.043	3.122	1.611	4.03	-0.422	0.124	0.536	54	49	87
59	5.378	2.858	6.223	2.454	2.760	1.818	3.58	0.320	-0.112	0.410	55	63	89
60	5.192	3.113	4.068	1.748	3.031	1.885	3.17	-0.012	0.510	0.510	56	4	135

61	6.650	5.073	7.718	3.424	3.024	1.508	4.57	0.193	-0.404	0.469	57	61	60
62	6.346	2.825	5.496	1.809	2.123	0.294	3.15	-0.234	-0.381	0.478	58	21	131
63	5.066	3.159	6.105	3.15	3.514	1.427	3.74	0.327	0.001	0.404	59	54	76.5
64	4.873	2.790	5.578	2.469	2.632	2.108	3.41	0.362	0.167	0.477	60	71	113
65	6.658	3.689	5.252	3.915	2.946	1.801	4.04	-0.278	0.174	0.385	61	11	57
66	5.882	2.769	6.531	1.707	2.574	0.853	3.39	0.165	-0.494	0.535	62	43	126
67	7.281	2.940	5.327	2.073	3.091	1.297	3.67	-0.417	-0.205	0.553	63	19	108
69	5.930	2.539	5.892	4.492	3.086	1.938	3.98	0.044	0.022	0.059	64	10	20
70	6.057	3.522	4.577	1.919	2.396	1.588	3.34	-0.199	0.237	0.341	65	45	92
71	8.149	3.060	6.626	3.975	3.709	1.837	4.56	-0.409	-0.421	0.657	66	60	88
72	5.362	2.825	4.406	1.698	2.244	0.972	2.92	-0.098	0.161	0.201	67	68	90
80	6.161	3.093	4.811	1.708	2.027	1.244	3.17	-0.215	0.019	0.266	68	28	85
86	5.896	2.894	5.433	1.889	2.505	0.911	3.26	-0.055	-0.166	0.180	69	77	70
87	6.427	2.935	5.023	2.270	1.944	1.849	3.41	-0.207	0.045	0.259	70	56	70.5
88	5.933	2.743	4.183	1.981	2.556	1.437	3.14	-0.257	0.251	0.404	71	16	118
90	7.263	3.042	7.054	0.943	4.380	1.956	4.11	0.057	-0.548	0.552	72	90	87
91	5.754	2.981	5.265	1.347	2.807	1.583	3.29	0.047	0.010	0.059	73	14	62
92	3.946	3.070	5.530	1.567	2.266	1.118	2.92	0.533	0.089	0.664	74	34	165
93	4.266	2.920	5.474	1.724	2.096	1.416	2.98	0.457	0.099	0.572	75	32	152
94	6.378	2.359	5.396	1.502	2.470	0.797	3.15	-0.193	-0.326	0.404	76	20	117
97	6.666	2.927	5.937	1.397	2.672	1.384	3.5	-0.099	-0.335	0.356	77	75	83
98	8.550	4.627	6.053	4.671	5.372	1.868	5.19	-0.630	-0.011	0.778	78	35	91
99	6.880	3.393	4.968	1.539	2.637	1.289	3.45	-0.363	-0.055	0.452	79	9	103
102	6.967	2.807	4.665	1.830	3.117	0.929	3.39	-0.488	-0.080	0.607	80	56	136
104	5.710	3.965	5.520	1.741	3.013	1.778	3.62	0.117	0.131	0.195	81	48	47
105	6.356	3.241	4.864	2.439	2.539	1.573	3.50	-0.244	0.121	0.325	82	52	77
114	6.075	3.444	5.429	2.115	2.002	1.064	3.36	-0.106	-0.105	0.168	83	5	64
115	6.909	3.608	6.405	5.096	2.708	1.508	4.37	-0.201	-0.175	0.304	84	1	35
124	5.091	2.895	6.173	2.157	1.820	1.379	3.25	0.341	-0.194	0.463	85	80	118
127	3.756	2.406	4.983	1.597	1.663	1.665	2.68	0.527	0.254	0.697	86	41	174

128	5.266	3.222	5.634	1.583	2.308	1.426	3.24	0.221	-0.035	0.274	87	87	84
143	7.748	4.56	4.128	3.999	3.738	2.348	4.42	-0.734	0.579	1.075	88	69	99
144	5.704	1.628	3.648	0.898	2.632	1.393	2.65	-0.259	0.225	0.392	89	2	131
146	7.135	2.662	5.721	2.493	3.383	1.829	3.87	-0.254	-0.194	0.369	90	13	65
CS	7.167	4.32	4.317	1.359	2.388	1.910	3.58	-0.503	0.284	0.682	91	86	127
SQ1	4.945	3.11	4.641	1.603	2.708	1.577	3.10	0.137	0.308	0.351	92	7	108
Mean	5.961	3.158	5.479	2.216	2.856	1.59	3.54						
Variance	1.603	0.704	1.23	1.045	0.544	0.329	3.54						
IPCAe1	-2.174	-0.006	1.535	-0.296	0.119	0.823							
IPCAe2	-0.915	0.844	-1.916	0.407	0.318	1.262							

IPCAg1, IPCAg2, principal components of interaction for genotypes; IPCAe1, IPCAe2, principal components of interaction for environments.

Table S3. Average TGW (in g), for genotypes and environments, the principal component analysis values, the AMMI stability value (ASV), rank of the AMMI stability value (RA), rank of trait mean (RM), and genotype selection index (GSI) of tested DH lines and their parental components (CS and SQ1)

Line	2010,C	2010,D	2012,C	2012,D	2013,C	2013,D	Mean	IPCAg1	IPCAg2	ASV	RA	RM	GSI
1	29.19	21.89	37.00	15.00	33.67	17.33	25.7	1.681	0.562	2.070	87	73	160
2	33.00	27.67	35.33	22.67	30.33	21.00	28.3	1.134	0.095	1.348	72	39	111
3	22.8	13.49	35.33	19.67	26.33	19.33	22.8	0.246	0.444	0.531	24	85	109
4	23.07	18.29	33.33	22.33	28.33	13.67	23.2	0.190	0.840	0.870	49	83	132
5	32.15	28.35	39.67	35.33	34.67	22.33	32.1	-0.309	0.702	0.792	42	8	50
7	28.88	20.49	32.33	25.00	27.33	21.00	25.8	0.218	0.011	0.259	6	69	75
8	23.35	15.25	28.67	20.00	30.67	18.33	22.7	0.228	-0.097	0.287	9	87	96
9	32.35	29.4	36.33	22.67	30.33	20.00	28.5	1.189	0.248	1.431	74	37	111
10	21.72	22.00	41.00	23.13	36.00	22.00	27.6	0.223	0.574	0.632	35	51	86
11	25.60	23.94	31.67	26.33	29.00	18.00	25.8	0.007	0.195	0.195	3	71	74
13	30.75	20.33	35.33	29.00	24.67	19.33	26.6	-0.070	0.612	0.618	34	62	96
14	32.21	25.7	36.67	30.00	24.67	20.67	28.3	0.119	0.494	0.514	22	40	62
15	28.47	22.38	37.33	34.00	33.00	22.67	29.6	-0.693	0.518	0.971	55	23	78
17	24.82	17.49	29.67	23.00	34.00	14.33	23.9	0.164	0.493	0.531	23	80	103
18	27.92	20.90	36.67	28.67	30.33	25.67	28.4	-0.232	-0.004	0.275	8	38	46
19	23.60	19.59	29.00	19.33	22.33	19.67	22.3	0.409	-0.368	0.609	32	90	122
20	32.64	26.94	35.33	35.33	31.67	26.67	31.4	-0.503	-0.156	0.616	33	12	45
21	30.25	26.79	32.00	31.00	28.67	25.33	29.0	-0.227	-0.478	0.549	26	33	59
22	26.54	17.62	34.33	27.00	31.33	19.00	26.0	-0.188	0.541	0.585	30	68	98
23	23.14	25.36	31.33	31.00	32.33	30.00	28.9	-0.830	-1.091	1.470	75	34	109
24	31.24	28.96	34.67	31.67	31.00	25.33	30.5	-0.086	-0.246	0.266	7	18	25
25	30.82	18.52	36.33	28.33	30.33	18.33	27.1	0.025	0.864	0.864	47	59	106
26	29.76	18.93	30.67	24.67	36.00	23.33	27.2	0.260	-0.340	0.459	18	57	75
27	31.93	24.97	37.33	29.67	31.00	21.00	29.3	0.170	0.545	0.581	29	29	58
28	29.66	31.47	43.00	35.00	41.33	38.00	36.4	-0.448	-0.821	0.977	56	1	57
29	28.24	25.97	32.00	23.00	18.33	20.00	24.6	0.557	-0.144	0.675	38	78	116

30	26.09	21.64	35.67	33.00	33.00	22.85	28.7	-0.804	0.304	1.000	57	35	92
31	26.81	25.97	31.33	24.00	32.00	22.00	27.0	0.401	-0.378	0.608	31	61	92
32	27.54	22.10	34.33	19.00	23.33	16.67	23.8	0.975	0.443	1.238	66	81	147
33	32.65	27.88	42.67	29.67	31.67	22.85	31.2	0.410	0.779	0.919	54	14	68
34	21.14	20.41	33.00	39.00	32.00	22.67	28.0	-1.965	0.215	2.339	92	44	136
35	27.39	22.80	31.00	30.67	27.67	22.85	27.1	-0.525	-0.236	0.666	37	60	97
36	25.72	27.53	32.00	33.67	31.33	37.67	31.3	-1.000	-1.834	2.184	91	13	104
37	27.57	23.04	27.67	17.67	30.00	24.33	25.1	0.975	-1.085	1.584	79	77	156
38	21.44	19.09	31.33	23.00	25.00	14.67	22.4	-0.053	0.509	0.513	21	89	110
39	33.05	33.97	37.67	36.00	27.00	26.33	32.3	-0.276	-0.073	0.335	13	6	19
40	26.04	24.97	33.33	34.33	27.00	22.00	28.0	-0.920	0.108	1.096	62	46	108
41	24.17	26.74	30.67	21.11	33.00	33.00	28.1	0.418	-1.791	1.859	84	42	126
42	20.98	17.17	25.67	26.91	28.67	15.67	22.5	-0.720	0.027	0.854	45	88	133
43	27.18	19.64	31.67	26.33	26.67	22.67	25.7	-0.134	-0.198	0.253	5	72	77
45	20.22	18.71	32.00	25.67	31.00	15.00	23.8	-0.415	0.624	0.795	43	82	125
46	25.51	26.90	38.67	33.33	29.67	23.00	29.5	-0.655	0.426	0.886	51	26	77
47	24.54	18.12	35.67	30.33	30.00	19.33	26.3	-0.695	0.685	1.072	60	66	126
48	23.97	24.29	41.67	36.33	31.00	21.67	29.8	-1.128	1.000	1.669	81	21	102
49	23.45	18.04	27.67	11.67	21.00	23.33	20.9	1.161	-1.083	1.751	83	92	175
50	26.48	21.45	33.33	28.33	33.33	24.67	27.9	-0.299	-0.242	0.429	16	47	63
51	30.81	19.55	39.00	39.00	27.67	27.33	30.6	-1.314	0.387	1.604	80	17	97
52	29.53	26.27	33.67	27.00	28.33	20.00	27.5	0.292	0.163	0.382	14	55	69
53	34.97	24.35	38.00	30.00	37.33	27.67	32.1	0.278	-0.054	0.333	11	9	20
54	28.77	26.03	30.00	28.13	29.67	26.67	28.2	-0.069	-0.891	0.895	52	41	93
55	21.02	21.28	32.00	37.67	30.67	24.00	27.8	-1.833	-0.094	2.174	89	49	138
56	29.80	23.45	34.67	32.33	28.67	18.33	27.9	-0.382	0.672	0.811	44	48	92
57	32.30	26.11	37.00	32.00	34.00	28.67	31.7	-0.140	-0.289	0.334	12	11	23
58	34.11	21.97	38.33	30.33	31.33	21.33	29.6	0.146	0.726	0.747	41	24	65
59	26.84	24.88	33.00	26.67	34.33	22.33	28.0	0.091	-0.146	0.181	2	45	47
60	33.75	32.65	41.00	22.67	35.33	30.67	32.7	1.376	-0.549	1.720	82	5	87

61	29.59	28.36	24.67	27.33	32.00	32.67	29.1	-0.008	-2.132	2.132	88	31	119
62	27.07	20.05	32.33	31.67	29.33	22.85	27.2	-0.730	-0.008	0.865	48	58	106
63	27.12	27.78	33.00	31.00	30.67	25.67	29.2	-0.377	-0.476	0.653	36	30	66
64	23.06	21.18	30.67	25.00	29.33	16.33	24.3	-0.105	0.293	0.318	10	79	89
65	30.22	28.28	35.67	22.33	23.33	16.67	26.1	1.023	0.531	1.323	71	67	138
66	29.34	21.84	33.33	23.67	28.33	22.00	26.4	0.472	-0.070	0.564	28	64	92
67	23.99	22.85	35.00	33.00	25.33	18.00	26.4	-0.903	0.701	1.279	69	65	134
69	21.18	19.12	35.67	31.67	34.67	24.00	27.7	-1.085	0.154	1.295	70	50	120
70	27.22	25.73	30.33	25.67	29.67	15.33	25.7	0.286	0.318	0.465	19	74	93
71	30.14	21.29	30.33	21.67	29.33	17.67	25.1	0.761	0.095	0.907	53	76	129
72	29.56	22.93	41.00	35.33	40.00	25.00	32.3	-0.665	0.663	1.030	58	7	65
80	27.29	25.68	32.00	36.67	29.00	28.33	29.8	-1.192	-0.654	1.557	78	20	98
86	29.28	20.79	28.67	26.67	30.33	23.33	26.5	-0.026	-0.543	0.544	25	63	88
87	23.35	19.11	30.33	17.00	23.67	20.00	22.2	0.683	-0.331	0.874	50	91	141
88	30.03	23.00	37.33	33.00	30.33	22.67	29.4	-0.464	0.483	0.732	39	28	67
90	34.85	27.38	38.00	28.47	37.33	31.67	33.0	0.504	-0.623	0.863	46	4	50
91	34.48	28.43	40.33	25.48	35.00	28.00	32.0	0.958	-0.117	1.141	64	10	74
92	21.73	22.06	26.33	23.77	29.67	31.33	25.8	-0.331	-1.855	1.896	85	70	155
93	22.38	21.22	32.33	18.67	27.33	17.00	23.2	0.610	0.183	0.746	40	84	124
94	32.26	18.4.0	36.33	25.44	31.33	35.00	29.8	0.217	-1.045	1.077	61	22	83
97	34.61	21.14	39.67	31.67	31.67	18.33	29.5	0.066	1.252	1.255	68	26	94
98	36.47	22.25	36.00	22.00	33.00	22.33	28.7	1.265	0.181	1.510	77	36	113
99	30.55	25.35	35.00	21.33	27.67	24.00	27.3	0.962	-0.281	1.175	65	56	121
102	30.6.0	27.00	29.33	32.33	29.33	20.00	28.1	-0.319	-0.102	0.391	15	43	58
104	23.37	26.13	41.33	33.33	32.33	17.67	29.0	-0.678	1.275	1.507	76	32	108
105	33.68	27.98	40.67	34.00	36.67	34.00	34.5	-0.202	-0.502	0.556	27	2	29
114	30.12	28.19	32.67	38.00	35.00	35.67	33.3	-1.113	-1.384	1.912	86	3	89
115	30.73	29.54	25.33	26.67	29.82	22.85	27.5	0.341	-1.023	1.100	63	54	117
124	26.81	21.00	34.33	27.33	23.33	19.33	25.4	-0.151	0.404	0.442	17	75	92
127	31.31	28.67	32.67	30.33	31.00	23.00	29.5	0.064	-0.206	0.220	4	27	31

128	33.82	29.80	37.00	32.33	28.67	25.67	31.2	0.068	-0.042	0.091	1	15	16
143	32.07	19.60	39.00	23.67	29.67	21.33	27.6	0.710	0.636	1.054	59	53	112
144	21.08	17.20	51.67	26.91	35.33	27.67	30.0	-0.333	1.183	1.247	67	19	86
146	32.26	25.04	31.00	21.00	38.00	18.33	27.6	1.198	0.015	1.421	73	52	125
CS	25.75	21.27	28.00	21.67	24.00	16.00	22.8	0.393	-0.005	0.466	20	86	106
SQ1	34.54	32.72	43.67	22.33	32.00	19.67	30.8	1.668	0.913	2.178	90	16	106
Mean	28.15	23.57	34.42	27.68	30.36	22.85	27.8						
Variance	30.38	27.21	37.24	47.5	25.03	39.51	49.9						
IPCAe1	3.290	1.780	0.932	-5.646	0.370	-0.727							
IPCAe2	0.490	-1.152	4.135	1.235	0.122	-4.831							

IPCAg1, IPCAg2, principal components of interaction for genotypes; IPCAe1, IPCAe2, principal components of interaction for environments.

Table S4. Average DUBOIS (µg mg^{-1} dry mass), for genotypes and environments, the principal component analysis values, the AMMI stability value (ASV), rank of the AMMI stability value (RA), rank of trait mean (RM), and genotype selection index (GSI) of tested DH lines and their parental components (CS and SQ1)

Line	2010,C	2010,D	2012,C	2012,D	2013,C	2013,D	Mean	IPCAg1	IPCAg2	ASV	RA	RM	GSI
1	111.2	93.38	69.06	35.79	33.95	41.69	64.19	0.372	-1.512	1.702	36	58	94
2	72.45	102.3	66.95	93.85	74.33	56.88	77.80	0.090	1.080	1.097	16	16	32
3	98.39	105.5	75.24	43.73	43.2	60.23	71.05	-0.427	-1.242	1.533	29	37	66
4	73.87	70.56	65.72	62.69	39.78	85.14	66.29	-2.225	1.716	4.988	81	52	133
5	101.1	95.29	94.72	36.94	38.14	77.51	73.95	-1.177	-0.285	2.493	54	27	81
7	86.73	109.6	103.0	26.8	33.11	170.47	88.29	-6.265	-0.172	13.19	91	6	97
8	112.7	90.02	72.38	43.50	36.09	42.86	66.26	0.484	-0.980	1.414	25	53	78
9	88.68	110.2	98.53	56.38	87.90	181.66	103.9	-6.553	0.952	13.82	92	1	93
10	89.40	116.9	106.5	48.43	41.06	47.73	74.99	0.945	-0.644	2.09	39	22	61
11	57.18	57.77	98.38	57.02	40.98	37.88	58.20	0.557	3.244	3.449	65	76	141
13	82.43	113.0	101.9	52.73	40.42	39.21	71.61	1.286	-0.334	2.727	58	35	92.5
14	79.98	99.93	67.16	50.92	93.22	151.77	90.50	-5.821	0.362	12.25	90	5	95
15	76.07	95.15	115.8	88.83	27.06	68.03	78.48	0.407	2.576	2.714	57	14	71
17	83.35	111.9	108.6	103.5	28.04	33.69	78.17	2.639	1.539	5.763	84	15	99
18	96.59	91.21	81.39	38.69	21.49	42.01	61.90	0.490	-0.645	1.216	20	66	86
19	73.93	105.0	77.94	60.77	36.71	38.38	65.46	0.862	-0.196	1.824	37	56	93
20	107.7	121.4	88.84	69.94	34.22	81.53	83.94	-0.590	-0.884	1.524	28	8	36
21	110.7	82.78	131.2	50.14	30.80	40.52	74.35	1.759	1.434	3.97	74	26	100
22	96.05	81.40	59.29	32.73	32.43	48.84	58.46	-0.533	-0.882	1.427	26	75	101
23	84.99	109.4	108.0	58.62	34.68	34.06	71.61	1.793	0.124	3.774	71	35	106
24	91.88	72.46	77.82	51.61	39.20	92.87	70.97	-2.399	1.256	5.201	83	38	121
25	110.1	91.27	77.54	62.39	33.70	40.85	69.31	1.014	-0.213	2.144	42	44	86
26	86.81	79.89	51.49	66.61	33.36	44.33	60.42	0.045	0.296	0.311	2	70	72
27	82.14	119.0	91.70	51.75	27.75	31.53	67.32	1.584	-1.114	3.514	68	49	117
28	86.89	93.28	97.74	85.52	28.01	36.11	71.37	1.850	1.528	4.183	76	36	112
29	64.99	91.74	69.61	52.15	26.21	34.18	56.48	0.582	0.097	1.229	22	79	101

30	145.2	90.75	74.66	56.44	31.00	84.85	80.48	-1.160	-0.952	2.62	55	10	65
31	114.8	106.3	90.62	63.90	36.78	39.12	75.26	1.571	-0.678	3.374	64	21	85
32	79.43	90.49	54.80	52.33	28.27	79.45	64.13	-2.021	-0.264	4.261	77	59	136
33	97.90	98.84	106.6	51.22	44.96	43.68	73.86	1.122	0.226	2.373	48	28	76
34	82.77	72.70	77.04	48.44	31.39	42.26	59.10	0.211	0.910	1.013	9	73	82
35	103.5	75.74	88.40	31.31	34.17	59.64	65.47	-0.576	0.183	1.225	21	55	76
36	74.54	123.7	85.60	69.49	30.98	35.59	69.98	1.514	-0.730	3.269	62	41	103
37	83.21	101.7	65.10	33.12	32.36	45.11	60.09	-0.155	-1.527	1.561	32	72	104
38	64.35	72.59	62.07	36.69	25.48	43.17	50.72	-0.513	0.451	1.17	18	89	107
39	105.8	98.53	84.42	44.69	50.67	62.56	74.44	-0.371	-0.669	1.028	13	25	38
40	65.69	74.66	88.93	65.87	33.20	48.84	62.86	0.214	2.200	2.246	45	63	108
41	70.55	67.57	61.13	48.16	29.67	48.04	54.19	-0.587	1.000	1.589	33	83	116
42	54.49	67.44	72.18	35.76	29.05	36.88	49.30	-0.162	1.197	1.244	23	91	114
43	101.4	98.21	102.7	47.82	47.04	43.38	73.43	1.033	-0.045	2.174	43	31	74
45	89.78	69.62	89.88	66.37	35.70	44.19	65.92	0.699	1.956	2.448	51	54	105
46	92.67	129.5	92.52	45.57	57.17	130.98	91.4	-3.786	-1.242	8.064	89	4	93
47	111.5	84.37	85.69	62.55	30.96	40.41	69.25	1.149	0.367	2.446	50	45	95
48	116.9	99.53	63.03	46.57	56.63	39.83	70.41	0.611	-1.698	2.129	41	39	80
49	75.68	78.47	75.94	65.53	38.10	44.07	62.96	0.355	1.350	1.543	30	62	92
50	77.50	86.23	81.15	33.35	34.11	35.24	57.93	0.473	-0.131	1.003	8	77	85
51	109.8	105.3	85.45	60.23	56.82	55.61	78.87	0.395	-0.587	1.017	10	12	22
52	108.7	150.2	115.9	79.66	57.59	37.57	91.58	2.715	-1.520	5.912	85	2	87
53	119.6	167.7	83.94	49.60	44.82	82.92	91.43	-0.566	-4.439	4.596	80	3	83
54	90.24	122.2	73.37	74.27	88.17	80.70	88.15	-1.081	-0.626	2.36	47	7	54
55	61.32	78.05	66.32	47.97	36.63	28.77	53.18	0.541	0.683	1.328	24	86	110
56	85.47	110.4	109.9	58.27	51.67	33.99	74.95	1.799	0.181	3.789	72	23	95
57	98.36	95.66	78.74	41.09	64.61	43.91	70.39	0.330	-0.717	0.999	7	40	47
58	88.76	103.3	111.8	88.10	39.31	41.08	78.71	1.971	1.564	4.433	78	13	91
59	87.34	54.71	66.14	53.42	36.62	30.33	54.76	0.604	1.554	2.008	38	81	119
60	113.4	117.4	95.63	25.00	54.78	49.11	75.88	0.481	-2.266	2.482	53	19	72

61	86.28	114.7	71.09	65.20	46.31	69.13	75.45	-0.662	-0.782	1.598	34	20	54
62	91.16	67.94	80.25	49.16	34.36	45.74	61.44	0.132	1.141	1.174	19	68	87
63	77.21	127.0	76.86	77.65	45.16	35.62	73.24	1.516	-0.893	3.313	63	32	95
64	80.31	69.91	69.30	40.24	47.85	35.11	57.12	0.214	0.622	0.767	3	78	81
65	75.19	97.26	76.74	78.15	37.23	100.92	77.58	-2.324	1.212	5.039	82	17	99
66	81.77	87.31	97.35	44.99	50.21	55.77	69.56	-0.119	0.828	0.865	4	42	46
67	101.5	119.8	86.92	34.75	33.63	30.64	67.86	1.444	-2.291	3.805	73	47	120
69	73.32	81.96	80.37	65.30	33.58	34.69	61.54	0.966	1.253	2.387	49	67	116
70	71.19	116.8	69.41	36.58	36.50	33.98	60.75	0.599	-1.889	2.271	46	69	115
71	68.88	96.45	76.86	46.71	40.55	45.41	62.48	0.063	-0.063	0.146	1	65	66
72	96.87	69.11	58.67	33.07	31.77	17.83	51.22	1.041	-0.473	2.241	44	88	132
80	81.05	112.9	54.19	36.25	30.82	18.05	55.54	1.257	-2.513	3.648	70	80	150
86	86.78	77.69	54.22	29.86	29.76	31.92	51.71	0.113	-0.862	0.894	6	87	93
87	89.58	55.78	88.47	30.63	32.50	24.16	53.52	1.005	1.286	2.476	52	85	137
88	85.49	91.72	107.0	82.58	44.39	45.72	76.16	1.371	1.985	3.502	67	18	85
90	76.18	103.6	82.61	59.44	39.86	68.84	71.75	-0.731	0.168	1.547	31	33	64
91	84.98	90.92	77.95	56.05	32.13	38.57	63.43	0.762	0.140	1.61	35	60	95
92	73.40	97.99	63.47	59.89	37.46	29.22	60.24	0.996	-0.338	2.122	40	71	111
93	73.91	111.7	77.79	35.91	37.57	104.78	73.61	-3.133	-0.919	6.658	88	29	117
94	82.88	90.96	60.44	57.38	38.29	70.19	66.69	-1.305	-0.045	2.746	59	50	109
97	88.38	92.23	84.50	44.44	88.18	105.61	83.89	-3.065	0.538	6.472	87	9	96
98	84.01	51.52	87.98	51.49	37.92	38.06	58.50	0.491	2.463	2.671	56	74	130
99	91.48	118.2	78.62	53.43	75.85	23.76	73.55	1.758	-1.459	3.977	75	30	105
102	67.62	109.1	60.85	51.75	45.54	44.14	63.17	0.015	-1.022	1.022	12	61	73
104	116.9	138.4	61.60	59.91	42.64	54.62	79.02	0.428	-3.344	3.463	66	11	77
105	64.51	56.04	56.16	35.82	21.72	18.48	42.12	0.555	0.934	1.496	27	92	119
114	88.80	104.5	96.77	41.97	34.99	49.79	69.47	0.425	-0.490	1.019	11	43	54
115	74.59	106.1	47.80	69.34	40.81	53.20	65.31	-0.356	-0.768	1.074	15	57	72
124	77.84	60.73	63.35	42.20	27.61	30.77	50.42	0.308	0.936	1.139	17	90	107
127	75.69	92.76	96.60	45.18	38.03	59.21	67.91	-0.299	0.624	0.886	5	46	51

128	58.04	101.1	105.5	41.03	43.55	26.06	62.55	1.477	0.481	3.146	61	64	125
143	82.05	80.16	55.4	52.62	35.19	93.47	66.48	-2.858	0.397	6.027	86	51	137
144	61.38	43.23	91.02	54.52	34.11	39.25	53.92	0.221	3.605	3.635	69	84	153
146	68.65	68.72	64.10	48.33	37.40	38.89	54.35	-0.059	1.045	1.052	14	82	96
CS	112.4	131.2	56.76	32.99	43.00	70.96	74.54	-1.148	-3.796	4.499	79	24	103
SQ1	80.66	108.3	104.6	36.54	44.02	32.84	67.82	1.325	-0.542	2.84	60	48	108
Mean	87.05	95.06	81.17	52.68	40.82	53.33	68.35						
Variance	460.5	904.5	397.4	310.0	238.0	980.2	956.5						
IPCAe1	2.811	2.524	4.901	4.575	-0.669	-14.142							
IPCAe2	-4.004	-9.670	5.461	6.106	0.799	1.308							

IPCAg1, IPCAg2, principal components of interaction for genotypes; IPCAe1, IPCAe2, principal components of interaction for environments.

Table S5. Average glucose (µg mg^{-1} dry mass), for genotypes and environments, the principal component analysis values, the AMMI stability value (ASV), rank of the AMMI stability value (RA), rank of trait mean (RM), and genotype selection index (GSI) of tested DH lines and their parental components (CS and SQ1)

Line	2010,C	2010,D	2012,C	2012,D	2013,C	2013,D	Mean	IPCAg1	IPCAg2	ASV	RA	RM	GSI
1	28.42	23.43	13.29	12.02	3.21	16.69	16.2	-0.214	0.959	1.03	39	34	73
2	18.44	36.66	18.24	24.87	34.77	22.44	25.9	-0.626	-0.886	1.44	56	1	57
3	18.98	22.26	13.60	15.39	4.77	10.38	14.2	0.162	0.393	0.49	12	55	67
4	15.65	33.97	14.79	12.75	7.54	23.94	18.1	-0.750	-1.106	1.75	66	22	88
5	27.03	31.42	17.31	12.58	8.50	13.83	18.4	0.344	0.207	0.66	18	19	37
7	12.42	29.87	16.25	10.53	7.21	11.81	14.7	0.137	-0.883	0.92	33	51	84
8	14.66	21.94	11.09	14.77	3.56	4.80	11.8	0.483	0.110	0.88	31	77	108
9	10.06	26.05	14.49	12.27	22.03	46.91	22.0	-3.433	-1.141	6.32	91	10	101
10	21.07	30.89	17.97	15.40	5.00	12.28	17.1	0.482	-0.199	0.89	32	30	61.5
11	17.15	21.41	18.02	11.80	8.17	17.79	15.7	-0.521	0.206	0.97	34	36	70
13	10.27	17.10	13.57	10.58	7.00	7.26	11.0	-0.029	0.109	0.12	3	84	87
14	14.77	32.80	11.79	17.38	11.18	44.52	22.1	-2.717	-1.288	5.08	90	9	99
15	15.78	40.26	21.39	18.52	3.46	11.91	18.6	0.934	-1.419	2.21	74	18	92
17	13.91	24.20	19.64	23.30	5.68	5.03	15.3	0.936	-0.023	1.69	64	41	105
18	13.86	17.25	13.40	11.48	4.34	4.72	10.8	0.326	0.432	0.73	22	85	107
19	13.87	24.23	9.08	10.76	3.53	6.06	11.3	0.295	-0.225	0.58	15	83	98
20	11.58	37.56	28.13	17.58	9.61	27.07	21.9	-0.465	-1.694	1.89	69	11	80
21	11.73	25.90	22.68	16.08	3.62	10.42	15.1	0.497	-0.495	1.03	38	46	84
22	12.64	19.36	13.93	10.31	4.79	8.73	11.6	0.011	0.084	0.09	2	79	81
23	17.61	41.88	22.55	16.64	5.14	5.62	18.2	1.559	-1.342	3.12	84	21	105
24	10.03	22.24	13.52	16.12	13.15	32.99	18.0	-2.146	-0.613	3.93	87	23	110
25	21.27	34.84	14.87	15.11	8.90	10.40	17.6	0.602	-0.514	1.21	46	26	72
26	20.35	31.51	17.11	11.11	11.25	11.37	17.1	0.354	-0.340	0.73	21	28	48.5
27	11.21	30.25	24.75	10.86	4.46	6.39	14.7	0.961	-0.919	1.97	71	52	123
28	16.42	31.50	16.52	26.03	9.81	23.31	20.6	-0.517	-0.659	1.14	44	12	56
29	10.97	18.72	13.55	11.66	5.64	7.78	11.4	0.036	0.028	0.07	1	82	83

30	30.12	21.35	10.11	9.81	3.21	15.26	15.0	-0.275	1.266	1.36	51	47	98
31	25.89	24.35	14.42	15.49	5.07	8.61	15.6	0.550	0.813	1.29	47	37	84
32	16.39	30.62	13.71	13.81	4.40	12.16	15.2	0.225	-0.601	0.73	21	43	63.5
33	15.89	19.28	25.18	23.80	5.52	12.22	17.0	0.379	0.512	0.86	29	31	60
34	15.97	15.87	12.68	7.95	4.22	6.43	10.5	0.084	0.667	0.68	19	87	106
35	23.56	18.95	16.31	10.75	5.43	13.31	14.7	-0.115	0.992	1.01	37	50	87
36	12.19	11.79	8.64	9.11	3.79	5.07	8.43	-0.124	0.723	0.76	23	92	115
37	17.51	26.84	9.11	9.86	4.30	4.75	12.1	0.543	-0.143	0.99	36	73	109
38	18.40	9.74	9.91	8.54	4.26	10.55	10.2	-0.531	1.355	1.66	62	89	151
39	20.11	38.97	22.81	17.55	4.48	15.48	19.9	0.673	-0.990	1.57	60	14	74
40	23.50	14.15	13.00	11.26	4.87	16.52	13.9	-0.663	1.361	1.82	67	57	124
41	18.46	20.18	6.91	14.27	8.05	13.20	13.5	-0.482	0.466	0.99	35	59	94
42	12.99	13.28	10.02	16.25	6.88	9.02	11.4	-0.289	0.700	0.87	30	81	111
43	19.88	24.51	16.71	14.31	6.76	9.40	15.3	0.399	0.287	0.78	24	42	66
45	27.45	20.05	23.75	16.62	8.61	19.71	19.4	-0.261	1.240	1.33	49	17	66
46	22.34	34.93	16.93	14.97	21.97	35.83	24.5	-1.781	-0.717	3.3	85	5	90
47	19.08	21.57	13.99	9.77	5.12	12.94	13.8	-0.174	0.370	0.49	11	58	69
48	15.33	25.44	16.96	13.32	12.91	6.88	15.1	0.449	-0.141	0.83	27	44	71
49	20.00	23.17	13.11	13.95	4.11	10.55	14.2	0.167	0.377	0.48	10	56	66
50	18.39	20.46	11.70	15.05	6.02	6.56	13.0	0.332	0.542	0.81	26	65	90.5
51	19.30	24.67	14.02	17.06	4.06	16.63	16.0	-0.245	0.147	0.47	9	35	44
52	12.07	32.11	27.24	18.70	7.54	5.17	17.1	1.316	-0.893	2.54	80	27	107
53	28.22	43.45	26.57	20.85	7.99	17.27	24.1	0.953	-0.670	1.85	68	6	74
54	10.46	35.81	19.93	13.55	19.41	21.28	20.1	-0.567	-1.616	1.91	70	13	83
55	9.31	27.45	11.42	15.02	6.64	6.05	12.7	0.419	-0.828	1.12	43	66	109
56	18.77	32.79	25.14	15.09	4.49	6.29	17.1	1.288	-0.461	2.38	77	30	107
57	36.14	32.56	25.07	14.40	12.37	15.06	22.6	0.688	0.911	1.54	58	8	66
58	26.55	46.96	35.82	31.08	4.68	7.87	25.5	2.431	-0.861	4.48	88	2	90
59	29.07	27.62	17.73	22.04	8.10	5.90	18.4	1.138	0.917	2.25	75	20	95
60	32.34	34.81	19.77	7.63	5.62	18.34	19.8	0.218	0.245	0.46	8	15	23

61	11.23	17.5	17.05	13.86	9.18	29.99	16.5	-1.864	-0.077	3.37	86	32	118
62	17.67	18.63	15.93	11.73	2.37	7.63	12.3	0.318	0.601	0.83	28	71	99
63	11.43	27.21	22.00	18.88	5.94	3.92	14.9	1.093	-0.521	2.05	72	48	120
64	7.48	27.48	12.18	11.79	4.59	5.13	11.4	0.473	-1.009	1.32	48	80	128
65	24.32	28.57	17.36	23.40	8.11	34.84	22.8	-1.467	0.085	2.66	82	7	89
66	9.79	22.27	13.52	8.47	2.70	6.25	10.5	0.265	-0.406	0.63	17	88	105
67	30.67	19.75	17.55	13.20	3.64	3.88	14.8	0.999	1.662	2.46	79	49	128
69	9.14	15.98	9.29	11.64	6.32	6.70	9.85	-0.162	0.114	0.32	4	90	94
70	11.07	16.56	14.67	8.24	3.15	5.11	9.80	0.224	0.221	0.46	7	91	98
71	12.91	41.68	17.96	14.27	10.1	10.14	17.8	0.771	-1.808	2.28	76	24	100
72	31.00	23.35	18.10	11.41	3.52	6.16	15.6	0.918	1.326	2.13	73	38	111
80	20.9	23.85	15.14	9.46	5.59	3.71	13.1	0.782	0.440	1.48	57	64	121
86	13.87	30.06	12.29	9.13	5.30	4.92	12.6	0.650	-0.726	1.38	52	67	119
87	19.55	20.15	16.69	11.96	7.19	11.10	14.4	0.046	0.596	0.6	16	54	70
88	7.13	22.19	20.05	30.97	19.73	18.28	19.7	-0.537	-0.463	1.08	42	16	58
90	16.94	20.84	11.67	16.12	5.09	17.05	14.6	-0.576	0.264	1.08	41	53	94
91	16.39	28.28	17.47	17.25	4.93	13.59	16.3	0.201	-0.360	0.51	13	33	46
92	15.27	21.41	12.58	16.16	3.58	6.17	12.5	0.432	0.211	0.81	26	68	93.5
93	14.48	40.99	15.71	11.38	5.46	5.27	15.6	1.162	-1.605	2.65	81	39	120
94	17.58	10.19	8.73	14.63	7.29	20.67	13.2	-1.416	1.188	2.83	83	63	146
97	22.87	27.16	11.20	15.43	25.91	49.87	25.4	-3.540	-0.174	6.41	92	3	95
98	15.00	29.58	16.93	13.67	11.32	5.77	15.4	0.716	-0.519	1.4	53	40	93
99	12.79	19.66	13.92	11.56	3.51	2.99	10.7	0.574	0.153	1.05	40	86	126
102	15.30	39.59	10.88	16.92	2.77	5.17	15.1	1.110	-1.375	2.43	78	45	123
104	16.31	16.64	14.04	10.84	2.98	19.28	13.4	-0.898	0.500	1.7	65	60	125
105	17.83	12.84	12.59	13.87	3.15	10.77	11.8	-0.249	1.098	1.19	45	75	120
114	18.94	11.11	19.85	10.71	4.47	9.29	12.4	0.024	1.358	1.36	50	70	120
115	23.14	28.29	16.01	18.47	7.20	13.31	17.7	0.286	0.222	0.56	14	25	39
124	21.22	14.88	11.25	13.07	5.62	13.31	13.2	-0.443	1.161	1.41	55	61	116
127	12.09	20.23	13.39	10.48	3.69	19.24	13.2	-0.894	-0.174	1.63	61	62	123

128	7.18	17.39	11.96	19.38	10.13	4.47	11.8	0.205	-0.042	0.37	5	78	83
143	24.78	41.30	12.29	13.53	8.96	47.92	24.8	-2.541	-1.287	4.77	89	4	93
144	19.54	11.21	9.40	13.24	5.75	11.83	11.8	-0.535	1.357	1.67	63	76	139
146	15.35	19.85	10.6	15.26	6.43	7.55	12.5	0.122	0.328	0.4	6	69	75
CS	24.99	14.50	11.31	10.80	4.08	7.77	12.2	0.097	1.551	1.56	59	72	131
SQ1	19.17	11.99	14.68	14.71	4.40	6.33	11.9	0.206	1.360	1.41	54	74	128
Mean	17.73	25.16	15.83	14.50	7.19	13.31	15.6						
Variance	44.26	86.56	32.13	26.13	30.11	113.4	84.2						
IPCAe1	1.726	3.055	3.161	1.525	-1.582	-7.885							
IPCAe2	5.555	-5.849	0.255	0.732	0.142	-0.834							

IPCAg1, IPCAg2, principal components of interaction for genotypes; IPCAe1, IPCAe2, principal components of interaction for environments.

Table S6. Average fructose (μg mg⁻¹ dry mass), for genotypes and environments, the principal component analysis values, the AMMI stability value (ASV), rank of the AMMI stability value (RA), rank of trait mean (RM), and genotype selection index (GSI) of tested DH lines and their parental components (CS and SQ1)

Line	2010,C	2010,D	2012,C	2012,D	2013,C	2013,D	Mean	IPCAg1	IPCAg2	ASV	RA	RM	GSI
1	22.38	21.56	10.88	12.43	2.95	16.99	14.5	0.023	0.647	0.65	11	43	54
2	18.61	33.58	15.80	23.96	40.19	31.66	27.3	-1.138	0.140	2.77	73	2	75
3	15.87	30.57	11.57	14.46	4.41	9.86	14.5	0.673	-0.492	1.708	49	44	93
4	13.54	31.47	13.48	13.57	8.75	24.03	17.5	-0.531	-0.715	1.477	43	26	69
5	28.08	38.01	15.48	12.40	9.57	21.57	20.9	0.212	-0.548	0.752	14	15	29
7	10.59	30.33	15.37	8.74	8.52	12.36	14.3	0.278	-0.808	1.054	23	46	69
8	15.22	25.94	9.33	15.17	2.99	4.78	12.2	0.931	-0.065	2.263	63	72	135
9	8.27	31.47	11.15	11.76	33.65	50.38	24.5	-3.224	-0.769	7.874	90	7	97
10	18.67	37.53	14.44	14.13	4.63	16.69	17.7	0.421	-1.049	1.466	42	24	66
11	11.92	18.37	14.50	11.00	10.34	21.41	14.6	-0.699	0.488	1.767	52	42	94
13	10.10	18.55	11.40	9.26	7.91	9.25	11.1	0.156	0.326	0.499	7	78	85
14	10.51	29.78	10.29	15.63	12.99	74.62	25.6	-4.841	-0.888	11.8	92	5	97
15	17.08	28.14	16.40	17.71	3.58	14.91	16.3	0.444	-0.109	1.084	26	34	60
17	11.38	23.88	15.77	19.22	5.09	5.53	13.5	0.948	0.109	2.308	64	56	120
18	12.98	16.85	11.88	11.15	3.53	4.32	10.1	0.695	0.653	1.811	53	87	140
19	12.47	24.65	8.22	9.65	3.01	6.08	10.7	0.595	-0.238	1.466	41	82	123
20	10.13	46.81	24.42	15.69	12.64	38.70	24.7	-1.165	-2.306	3.652	82	6	88
21	9.60	38.45	19.66	15.55	3.45	12.69	16.6	0.719	-1.566	2.348	65	29	94
22	11.14	20.31	12.10	10.48	5.12	11.63	11.8	0.109	0.181	0.321	2	75	77
23	14.15	41.11	18.70	14.47	5.27	7.02	16.8	1.284	-1.551	3.485	81	27	108
24	8.91	24.19	10.15	15.55	14.64	38.92	18.7	-2.153	-0.201	5.238	87	18	105
25	18.63	34.66	12.61	12.74	11.69	17.25	17.9	0.141	-0.691	0.771	15	21	36
26	16.99	26.83	14.88	10.71	13.56	13.55	16.1	0.221	0.021	0.538	8	35	43
27	10.86	30.70	20.01	10.89	4.49	9.70	14.4	0.729	-0.799	1.945	57	45	102
28	12.88	27.51	13.05	21.68	10.93	30.69	19.5	-1.071	-0.164	2.61	70	16	86
29	8.85	17.33	11.16	10.87	6.04	10.36	10.8	0.053	0.376	0.397	6	80	86

30	26.06	21.45	8.04	8.85	2.71	13.03	13.4	0.294	0.770	1.051	22	58	80
31	24.12	25.32	12.73	15.26	5.75	15.30	16.4	0.354	0.510	1	20	31	51
32	17.01	36.92	11.95	14.74	5.30	12.19	16.4	0.662	-1.049	1.92	56	33	89
33	16.90	22.48	19.96	23.62	6.10	20.93	18.3	-0.004	0.628	0.628	10	20	30
34	12.58	15.58	10.14	7.58	4.41	6.77	9.51	0.334	0.660	1.046	21	90	111
35	21.95	17.16	13.12	9.97	5.24	16.53	14.0	-0.064	1.064	1.075	25	52	77
36	9.46	9.71	7.04	8.14	4.02	6.87	7.54	0.042	1.054	1.059	24	92	116
37	13.26	25.62	8.45	9.25	3.59	4.20	10.7	0.781	-0.277	1.918	55	81	136
38	13.60	9.39	8.86	8.74	3.56	13.75	9.65	-0.348	1.298	1.55	45	89	134
39	17.76	47.78	23.76	16.73	4.19	18.13	21.4	0.827	-2.000	2.835	75	14	89
40	19.21	14.75	11.26	11.11	4.50	20.77	13.6	-0.553	1.129	1.756	50	54	104
41	14.83	15.66	5.74	14.26	8.75	19.76	13.2	-0.716	0.909	1.963	58	59	117
42	10.61	13.56	9.01	15.19	7.07	11.52	11.2	-0.076	0.948	0.966	17	77	94
43	19.30	23.00	12.92	12.97	5.85	10.94	14.2	0.492	0.447	1.277	34	48	82
45	20.54	20.03	17.73	15.35	9.97	22.23	17.6	-0.333	0.919	1.225	32	25	57
46	21.05	40.06	14.77	15.24	28.47	40.80	26.7	-1.695	-0.846	4.207	84	4	88
47	21.40	20.60	12.08	9.18	4.97	15.88	14.0	0.013	0.662	0.663	12	51	63
48	15.85	24.54	13.09	12.54	17.65	7.21	15.2	0.580	0.323	1.446	40	40	80
49	16.75	21.10	11.39	13.04	3.73	11.01	12.8	0.366	0.447	0.995	19	60	79
50	15.81	20.47	9.78	14.80	6.17	6.42	12.2	0.652	0.555	1.679	48	72	120
51	20.62	29.07	10.82	15.69	3.57	18.47	16.4	0.077	-0.118	0.221	1	32	33
52	9.87	37.66	23.02	16.69	7.16	5.91	16.7	1.321	-1.316	3.47	80	28	108
53	26.34	49.25	20.66	18.56	7.50	16.89	23.2	1.083	-1.588	3.073	79	8	87
54	11.53	36.01	17.39	11.78	24.62	27.55	21.5	-0.899	-1.029	2.416	68	13	81
55	0.02	27.63	10.02	14.49	6.55	8.66	11.2	0.225	-1.043	1.178	28	76	104
56	16.52	30.88	18.57	13.84	4.25	9.95	15.7	0.874	-0.449	2.172	62	38	100
57	30.25	28.57	19.19	14.65	16.75	20.20	21.6	0.211	0.731	0.893	16	12	28
58	25.07	42.71	27.45	26.54	3.85	10.09	22.6	1.831	-0.789	4.521	85	9	94
59	22.99	24.50	15.40	19.82	12.28	11.17	17.7	0.709	0.799	1.899	54	23	77
60	25.10	31.05	16.90	7.66	5.73	27.45	19.0	-0.492	-0.223	1.216	31	17	48

61	10.47	15.47	15.25	14.16	11.71	43.98	18.5	-2.564	0.683	6.271	88	19	107
62	15.65	20.83	13.74	10.39	2.00	13.38	12.7	0.181	0.323	0.546	9	64	73
63	9.53	28.46	18.57	17.30	6.75	3.93	14.1	1.155	-0.435	2.842	76	50	126
64	6.34	24.66	10.38	12.06	4.00	5.46	10.5	0.578	-0.481	1.486	44	83	127
65	19.51	24.08	15.20	22.84	11.04	42.57	22.5	-1.860	0.528	4.552	86	10	96
66	7.30	23.53	11.16	8.74	2.18	9.71	10.4	0.220	-0.444	0.696	13	85	97.5
67	29.05	33.46	13.51	12.96	3.77	3.37	16.0	1.635	-0.048	3.975	83	36	119
69	7.59	18.74	7.31	13.53	5.83	8.61	10.3	0.138	0.205	0.394	5	86	91
70	9.16	16.84	12.70	7.92	2.29	4.48	8.9	0.565	0.348	1.417	38	91	129
71	11.88	42.32	15.68	14.52	11.55	11.28	17.9	0.743	-1.731	2.501	69	22	91
72	26.26	20.13	15.91	10.86	3.38	5.89	13.7	1.091	1.074	2.862	77	53	130
80	17.15	21.86	12.99	9.20	4.45	5.44	11.9	0.809	0.355	1.998	59	74	133
86	12.08	29.38	11.46	8.92	4.97	5.26	12.0	0.809	-0.683	2.082	61	73	134
87	16.32	18.34	13.49	12.14	7.14	8.80	12.7	0.467	0.766	1.369	37	62	99
88	6.34	24.87	17.11	26.26	29.20	27.34	21.9	-1.077	0.222	2.627	71	11	82
90	16.08	34.50	10.10	15.68	5.69	16.45	16.4	0.199	-0.868	0.994	18	30	48
91	12.61	25.82	12.73	15.43	4.32	18.08	14.8	-0.132	-0.212	0.385	4	41	45
92	12.61	24.70	10.14	15.73	3.42	9.41	12.7	0.488	-0.083	1.19	29	64	93
93	13.94	36.20	13.04	11.28	4.99	6.06	14.3	1.034	-1.195	2.783	74	47	121
94	13.02	12.07	7.31	13.67	7.39	22.30	12.6	-0.998	1.128	2.674	72	66	138
97	20.38	33.73	8.70	14.54	32.62	53.21	27.2	-3.094	-0.310	7.529	89	3	92
98	12.82	26.48	15.33	13.18	15.73	9.63	15.5	0.450	-0.052	1.096	27	39	66
99	13.69	16.45	11.51	11.29	3.61	3.06	9.94	0.797	0.740	2.074	60	88	148
102	12.58	39.34	9.62	15.10	2.01	6.12	14.1	1.079	-1.571	3.058	78	49	127
104	15.00	15.97	11.33	11.48	2.39	25.00	13.5	-0.937	0.724	2.391	67	55	122
105	13.46	11.79	10.83	13.72	2.24	10.60	10.4	0.119	1.180	1.215	30	85	115
114	18.27	12.66	17.10	10.52	4.39	10.90	12.3	0.326	1.368	1.581	46	70	116
115	17.91	24.18	13.72	15.39	8.11	16.53	16.0	0.068	0.327	0.366	3	37	40
124	17.74	14.58	9.46	11.76	5.94	16.53	12.7	-0.309	1.114	1.343	36	64	100
127	10.91	21.63	9.69	12.61	3.63	22.13	13.4	-0.724	-0.014	1.76	51	57	108

128	5.57	23.14	9.85	18.91	11.66	4.81	12.3	0.583	-0.072	1.419	39	68	107
143	23.52	44.04	10.60	13.31	12.79	62.86	27.9	-3.264	-1.583	8.092	91	1	92
144	15.76	11.58	7.88	11.84	5.02	12.62	10.8	-0.143	1.298	1.343	35	79	114
146	16.02	16.52	9.79	15.58	7.91	9.59	12.6	0.303	0.993	1.236	33	67	100
CS	25.96	15.25	10.09	9.57	4.23	11.94	12.8	0.288	1.449	1.609	47	61	108
SQ1	22.30	11.20	11.88	16.57	4.16	7.73	12.3	0.619	1.854	2.387	66	70	136
Mean	15.55	25.72	13.25	13.74	8.12	16.53	15.5						
Variance	37.84	100.5	21.33	19.94	56.37	195	99.7						
IPCAe1	2.857	2.536	3.098	2.004	-1.487	-9.008							
IPCAe2	3.957	-7.131	0.567	1.721	1.270	-0.384							

IPCAg1, IPCAg2, principal components of interaction for genotypes; IPCAe1, IPCAe2, principal components of interaction for environments.

Table S7. Average sucrose (μg mg^{-1} dry mass), for genotypes and environments, the principal component analysis values, the AMMI stability value (ASV), rank of the AMMI stability value (RA), rank of trait mean (RM), and genotype selection index (GSI) of tested DH lines and their parental components (CS and SQ1)

Line	2010,C	2010,D	2012,C	2012,D	2013,C	2013,D	Mean	IPCAg1	IPCAg2	ASV	RA	RM	GSI
1	69.23	81.57	59.87	26.91	13.84	15.61	44.5	-0.671	-0.335	1.45	19	71	90
2	64.24	84.86	42.43	66.93	52.58	13.56	54.1	0.097	2.357	2.37	39	25	63.5
3	82.29	99.73	68.21	54.23	29.37	13.11	57.8	-0.923	0.895	2.14	35	15	50
4	64.41	46.94	51.48	50.13	29.45	20.25	43.8	2.066	0.694	4.39	78	72	150
5	74.88	114.75	51.82	19.87	34.12	9.07	50.8	-2.913	-0.546	6.14	87	41	128
7	85.08	112.60	75.42	43.65	35.96	11.22	60.7	-1.823	0.122	3.83	72	6	78
8	75.82	85.82	62.89	11.76	35.40	11.64	47.2	-1.117	-1.819	2.97	54	63	117
9	95.77	106.10	79.01	25.68	59.83	19.54	64.3	-1.471	-2.076	3.72	71	4	75
10	70.67	98.27	49.76	32.42	28.91	12.11	48.7	-1.640	0.247	3.45	69	56	125
11	56.50	49.11	81.05	61.93	36.12	12.17	49.5	2.586	1.389	5.61	86	49	135
13	78.17	114.75	64.38	42.01	43.30	10.69	58.9	-2.106	0.433	4.45	79	9	88
14	67.39	82.52	52.17	44.43	51.33	17.22	52.5	-0.098	0.533	0.57	5	34	39
15	69.82	59.39	66.75	57.35	23.86	13.46	48.4	1.556	1.050	3.43	65	59	124
17	91.83	80.54	62.64	68.70	33.63	12.00	58.2	0.532	1.153	1.61	24	13	37
18	102.60	96.93	64.11	32.87	35.73	16.03	58.1	-1.211	-1.479	2.94	53	14	67
19	68.04	80.99	60.85	60.75	29.98	14.51	52.5	0.290	1.714	1.82	32	33	65
20	96.78	98.87	72.08	39.50	42.91	12.07	60.4	-1.055	-0.932	2.4	40	7	47
21	100.50	67.51	74.56	39.59	31.25	21.40	55.8	0.944	-1.602	2.55	46	20	66
22	93.10	73.13	51.90	27.01	35.98	10.84	48.7	-0.147	-1.608	1.64	25	58	82.5
23	73.45	57.73	65.51	14.80	30.48	11.31	42.2	0.669	-1.963	2.41	42	76	118
24	77.32	69.16	70.71	57.75	27.56	23.66	54.4	1.206	0.697	2.63	48	24	72
25	81.23	57.20	50.96	40.92	31.33	13.03	45.8	1.131	-0.402	2.41	41	68	109
26	67.22	46.12	40.81	49.13	36.50	12.72	42.1	1.885	0.651	4.01	74	77	151
27	82.05	81.42	51.02	40.03	26.73	10.71	48.7	-0.445	-0.006	0.94	11	58	68.5
28	69.39	57.19	75.14	57.76	27.36	18.21	50.8	1.923	0.832	4.12	75	40	115
29	67.41	96.99	51.84	34.00	32.72	11.55	49.1	-1.471	0.395	3.12	57	52	109

30	111.1	90.61	63.96	58.26	33.22	13.75	61.8	-0.282	-0.227	0.64	7	5	12
31	81.02	70.97	69.32	50.09	31.04	14.32	52.8	0.790	0.144	1.67	26	30	56
32	66.69	51.73	38.78	33.99	23.60	21.94	39.5	1.181	-0.129	2.49	43	85	128
33	70.40	76.29	65.21	64.93	37.62	24.37	56.5	0.947	1.536	2.51	44	18	62
34	71.80	72.28	73.58	38.93	27.99	18.01	50.4	0.537	-0.234	1.15	13	42	55
35	98.55	57.77	63.43	30.86	32.67	14.86	49.7	1.088	-2.030	3.06	55	46	101
36	70.82	144.24	49.68	58.66	29.37	8.81	60.3	-3.887	2.762	8.62	92	8	100
37	72.90	129.86	62.41	12.88	31.74	13.47	53.9	-3.802	-0.846	8.03	90	28	118
38	66.09	50.39	45.51	37.61	21.22	8.63	38.2	1.237	0.182	2.61	47	89	136
39	85.40	79.12	50.22	23.82	34.96	23.85	49.6	-0.438	-1.486	1.75	31	48	79
40	57.47	54.37	78.86	55.05	28.66	12.15	47.8	2.007	1.114	4.36	76	61	137
41	65.07	67.87	57.96	49.39	22.62	9.77	45.5	0.654	1.068	1.74	30	69	99
42	56.28	69.95	60.72	23.76	23.12	14.86	41.5	0.042	-0.301	0.31	3	78	81
43	82.54	82.99	63.78	45.94	47.70	18.32	56.9	0.071	-0.161	0.22	1	17	18
45	48.81	59.91	54.15	44.79	30.52	9.50	41.3	1.035	1.297	2.53	45	79	124
46	76.89	95.98	66.36	51.56	37.33	24.31	58.7	-0.558	0.696	1.36	14	12	26
47	83.16	67.99	73.49	36.69	24.65	11.72	49.6	0.632	-0.831	1.57	22	47	69
48	75.08	95.56	60.53	36.54	52.79	15.12	55.9	-0.974	-0.223	2.06	33	19	52
49	62.48	66.95	58.89	43.28	18.93	11.10	43.6	0.569	0.783	1.43	17	73	90
50	92.86	70.90	70.00	28.50	18.95	12.81	49.0	0.180	-1.623	1.67	27	53	80
51	83.98	112.79	63.07	20.17	29.82	14.72	54.1	-2.570	-1.133	5.52	84	26	110
52	104.10	149.02	90.17	62.44	42.70	13.65	77.0	-3.300	0.802	6.98	88	1	89
53	66.51	96.99	59.70	22.63	23.23	23.29	48.7	-1.531	-0.439	3.25	60	55	115
54	77.58	104.05	40.40	56.15	45.13	10.74	55.7	-1.460	1.553	3.44	66	21	87
55	48.50	77.71	53.56	55.02	29.01	11.06	45.8	0.185	2.291	2.32	37	67	104
56	74.58	90.55	85.81	44.53	36.60	12.33	57.4	-0.279	0.082	0.59	6	16	22
57	58.27	63.03	58.74	24.62	44.76	11.03	43.4	0.583	-0.649	1.39	16	74	90
58	56.03	48.29	50.81	39.72	19.47	11.46	37.6	1.517	0.629	3.25	61	90	151
59	56.86	52.22	52.73	27.11	33.43	10.58	38.8	1.113	-0.364	2.37	39	87	126
60	83.33	87.48	54.81	12.04	47.70	11.41	49.5	-1.231	-2.126	3.35	63	50	113

61	66.83	96.80	53.92	64.88	32.66	12.91	54.7	-0.686	2.393	2.79	51	22	73
62	86.13	42.84	65.13	40.38	23.33	12.20	45.0	2.132	-0.999	4.59	81	70	151
63	64.92	97.47	57.45	56.83	30.69	13.84	53.5	-0.867	1.920	2.65	49	29	78
64	80.71	71.70	54.28	39.10	40.63	12.02	49.7	0.311	-0.414	0.77	9	45	54
65	63.61	76.07	63.77	38.46	20.39	20.01	47.1	0.111	0.381	0.45	4	64	68
66	73.02	72.13	69.91	44.10	44.41	11.37	52.5	0.654	-0.030	1.38	15	35	50
67	70.37	82.97	70.58	14.56	24.11	13.98	46.1	-0.825	-1.449	2.26	36	65	101
69	68.53	72.28	72.39	65.66	29.01	15.70	53.9	1.120	1.689	2.9	52	27	79
70	66.52	94.65	48.48	33.30	21.40	10.66	45.8	-1.501	0.554	3.2	59	66	125
71	58.67	68.88	57.06	41.17	36.99	23.86	47.8	0.707	0.502	1.57	23	60	83
72	69.09	50.80	44.70	34.44	31.49	13.49	40.7	1.282	-0.319	2.71	50	81	131
80	65.32	113.15	46.36	34.79	33.49	6.60	50.0	-2.590	0.937	5.52	85	44	129
86	89.29	40.05	46.71	26.17	25.96	8.12	39.4	1.660	-1.817	3.93	73	86	159
87	82.00	51.66	76.40	12.01	50.62	20.65	48.9	1.441	-3.144	4.37	77	54	131
88	69.77	46.19	66.69	40.17	49.13	13.48	47.6	2.173	-0.616	4.61	82	62	144
90	79.72	98.10	64.26	49.77	38.41	22.22	58.8	-0.781	0.534	1.72	29	11	39.5
91	72.78	78.81	65.01	57.95	24.32	13.69	52.1	0.363	1.306	1.51	20	37	57
92	72.29	65.13	68.49	66.05	31.77	9.24	52.2	1.441	1.527	3.39	64	36	100
93	81.33	88.70	64.60	35.23	44.66	12.96	54.6	-0.621	-0.630	1.45	18	23	41
94	64.71	105.42	61.12	63.33	35.53	22.46	58.8	-0.984	2.276	3.07	56	10	66
97	78.72	61.52	70.83	25.00	53.22	26.36	52.6	1.161	-1.995	3.15	58	31	89
98	76.72	18.84	59.52	41.16	25.72	8.98	38.5	3.508	-0.853	7.42	89	88	177
99	76.56	85.69	59.77	44.76	35.89	7.40	51.7	-0.439	0.396	1	12	39	51
102	54.54	69.55	41.92	33.92	22.05	16.45	39.7	0.039	0.722	0.73	8	84	91.5
104	104.4	143.63	52.08	42.98	30.77	15.33	64.9	-4.066	0.150	8.54	91	3	94
105	49.31	40.49	47.80	25.96	12.56	10.93	31.2	1.561	0.028	3.28	62	92	154
114	59.22	86.24	79.14	44.97	30.38	15.42	52.6	-0.116	0.851	0.89	10	32	42
115	58.41	83.9	33.85	32.01	32.74	14.86	42.6	-0.938	0.677	2.08	34	75	109
124	62.24	59.87	54.78	27.41	27.11	14.86	41.0	0.693	-0.446	1.52	21	80	101
127	71.59	80.28	72.45	36.29	27.60	22.30	51.8	0.026	-0.287	0.29	2	38	40

128	73.95	95.75	67.96	16.92	34.58	12.82	50.3	-1.520	-1.315	3.45	68	43	111
143	70.86	39.01	39.36	44.38	28.51	16.33	39.7	2.175	0.148	4.57	80	84	164
144	55.50	33.06	57.66	40.49	22.50	11.97	36.9	2.603	0.297	5.48	83	91	174
146	54.34	58.76	55.90	30.38	26.49	13.51	39.9	0.820	0.076	1.72	29	82	111
CS	112.3	106.61	62.91	33.43	42.89	46.82	67.5	-1.304	-2.102	3.45	68	2	69.5
SQ1	91.46	77.17	67.75	1.75	33.33	23.56	49.2	-0.590	-3.474	3.69	70	51	121
Mean	74.19	78.18	60.6	39.89	32.74	14.86	50.1						
Variance	260.4	744.2	190.6	262.3	104.4	36.5	787						
IPCAe1	0.066	-12.764	3.094	4.879	1.734	2.991							
IPCAe2	-6.210	2.412	-2.229	9.412	-1.827	-1.559							

IPCAg1, IPCAg2, principal components of interaction for genotypes; IPCAe1, IPCAe2, principal components of interaction for environments.

Table S8. Average maltose (µg mg⁻¹ dry mass), for genotypes and environments, the principal component analysis values, the AMMI stability value (ASV), rank of the AMMI stability value (RA), rank of trait mean (RM), and genotype selection index (GSI) of tested DH lines and their parental components (CS and SQ1)

Line	2010,C	2010,D	2012,C	2012,D	2013,C	2013,D	Mean	IPCAg1	IPCAg2	ASV	RA	RM	GSI
1	12.23	6.50	19.80	21.84	13.06	15.61	14.8	0.071	-0.556	0.57	11	36	47
2	3.07	0.79	21.66	26.31	25.46	13.56	15.1	1.412	0.374	2.76	79	33	112
3	4.06	2.40	20.70	27.40	8.48	13.11	12.7	0.399	0.276	0.82	19	59	78
4	3.03	25.55	22.75	22.82	13.36	20.25	18.0	-1.061	1.472	2.53	75	13	88
5	6.28	0.66	23.94	20.06	12.99	9.07	12.2	0.619	-0.346	1.25	44	70	114
7	5.00	2.49	23.79	15.14	10.64	11.22	11.4	0.396	-0.270	0.82	18	83	101
8	28.36	11.40	18.16	26.24	8.90	11.64	17.5	-1.057	-2.034	2.89	80	15	95
9	16.31	16.33	20.96	22.2	14.76	19.54	18.4	-0.632	-0.445	1.3	47	8	55
10	2.27	1.26	17.96	22.96	11.57	12.11	11.4	0.558	0.249	1.11	33	85	118
11	1.00	0.67	27.13	24.19	10.01	12.17	12.5	0.802	0.429	1.61	61	63	124
13	3.17	8.98	17.36	14.19	12.73	10.69	11.2	-0.177	0.179	0.39	3	87	90
14	0.73	1.97	22.11	22.04	18.60	17.22	13.8	1.111	0.563	2.23	74	48	122
15	7.89	14.5	25.62	31.42	10.64	13.46	17.3	-0.434	0.602	1.04	28	18	46
17	3.26	1.51	21.02	21.14	12.82	12.00	12.0	0.631	0.104	1.23	42	75	117
18	4.17	2.43	15.45	19.38	14.05	16.03	11.9	0.544	0.044	1.06	31	76	107
19	1.32	2.20	18.86	21.81	11.59	14.51	11.7	0.581	0.403	1.2	38	77	115
20	14.45	1.47	22.12	12.72	12.25	12.07	12.5	0.281	-1.418	1.52	56	64	120
21	5.95	1.86	25.82	23.15	11.67	21.40	15.0	0.863	0.077	1.68	64	35	99
22	13.77	32.21	21.30	22.17	8.15	10.84	18.1	-2.432	0.384	4.73	89	11	100
23	3.98	5.79	23.40	26.93	8.55	11.31	13.3	0.138	0.404	0.48	6	54	60
24	3.59	1.17	19.87	17.38	10.26	23.66	12.7	0.774	0.109	1.51	55	60	115
25	2.30	5.25	22.59	14.16	10.86	13.03	11.4	0.255	0.149	0.52	7	84	91
26	2.84	8.56	23.50	21.70	11.42	12.72	13.5	0.072	0.514	0.53	9	53	61.5
27	9.68	18.48	19.68	21.37	16.17	10.71	16.0	-0.847	0.196	1.65	62	28	90
28	5.33	8.60	23.73	32.00	17.48	18.21	17.6	0.539	0.743	1.28	46	14	60
29	0.78	1.88	16.75	20.29	10.62	11.55	10.3	0.430	0.327	0.9	23	92	115

30	4.06	0.72	16.73	23.41	11.44	13.75	11.7	0.567	0.069	1.1	32	80	112
31	34.94	42.25	30.94	28.94	8.76	14.32	26.7	-3.386	-1.120	6.66	92	2	94
32	7.92	17.55	19.05	27.54	9.64	21.94	17.3	-0.708	0.727	1.56	58	17	75
33	34.4	37.31	30.52	40.94	7.58	24.37	29.2	-2.662	-0.693	5.21	90	1	91
34	2.93	1.54	24.92	15.99	10.92	18.01	12.4	0.756	0.069	1.47	52	68	120
35	2.05	0.90	23.29	21.14	13.17	14.41	12.5	0.849	0.261	1.67	63	67	130
36	3.01	2.14	18.45	16.07	15.43	8.81	10.7	0.521	-0.072	1.01	27	90	117
37	1.76	3.07	25.67	17.72	13.39	13.47	12.5	0.692	0.265	1.37	48	65	113
38	8.50	1.39	21.43	22.94	12.97	8.63	12.6	0.451	-0.469	0.99	26	61	87
39	9.57	9.19	27.91	17.50	9.34	23.85	16.2	0.117	-0.138	0.27	2	25	27
40	3.78	1.24	27.43	21.23	16.17	12.15	13.7	0.967	0.081	1.88	71	49	120
41	13.44	12.93	18.63	29.22	12.43	9.77	16.1	-0.648	-0.237	1.28	45	27	72
42	1.76	0.41	24.24	15.56	13.05	14.86	11.7	0.879	0.081	1.71	65	81	146
43	17.43	2.60	20.80	19.98	20.90	18.32	16.7	0.728	-1.279	1.91	73	22	95
45	2.25	10.29	19.38	23.62	13.72	9.50	13.1	-0.117	0.668	0.71	15	57	72
46	26.60	18.20	24.10	26.23	17.88	24.31	22.9	-0.657	-1.226	1.77	67	3	70
47	33.86	1.89	24.62	17.40	12.53	11.72	17.0	-0.140	-3.351	3.36	82	21	103
48	3.06	1.23	18.74	19.39	14.44	15.12	12.0	0.749	0.107	1.46	51	74	125
49	6.21	3.42	29.49	17.71	13.17	11.10	13.5	0.572	-0.235	1.13	36	51	87
50	6.17	1.13	22.94	21.75	10.13	12.81	12.5	0.531	-0.203	1.05	29	67	95.5
51	33.06	22.89	21.62	17.51	7.90	14.72	19.6	-2.086	-2.258	4.64	88	6	94
52	21.01	7.50	19.70	20.31	15.90	13.65	16.3	-0.164	-1.543	1.58	59	24	83
53	14.54	12.41	25.37	22.17	10.46	23.29	18.0	-0.261	-0.378	0.63	12	12	24
54	8.93	0.65	18.68	31.30	18.11	10.74	14.7	0.810	-0.196	1.58	60	38	98
55	0.41	3.86	19.30	27.32	13.28	11.06	12.5	0.514	0.725	1.23	43	62	105
56	4.34	1.37	28.26	17.86	16.60	12.33	13.5	0.959	-0.086	1.86	70	53	123
57	5.39	5.66	21.83	18.00	10.68	11.03	12.1	0.095	-0.073	0.2	1	71	72
58	11.15	6.77	23.37	27.03	15.13	11.46	15.8	0.198	-0.292	0.48	5	30	35
59	12.68	5.64	22.41	18.18	12.49	10.58	13.7	0.004	-0.861	0.86	22	50	72
60	16.36	7.37	20.42	18.69	13.99	11.41	14.7	-0.183	-1.147	1.2	39	39	78

61	1.28	7.81	28.53	23.49	10.75	12.91	14.1	0.286	0.730	0.92	24	42	66
62	4.02	0.59	21.62	18.71	7.34	12.20	10.8	0.420	-0.134	0.83	20	89	109
63	1.32	2.34	23.00	21.98	9.78	13.84	12.0	0.567	0.408	1.17	37	73	110
64	9.50	10.53	16.28	22.27	13.38	12.02	14.0	-0.359	-0.129	0.71	16	45	60.5
65	6.38	42.87	24.68	29.38	8.03	20.01	21.9	-2.751	2.124	5.74	91	5	96
66	4.52	9.45	23.03	23.25	11.40	11.37	13.8	-0.081	0.402	0.43	4	47	51
67	1.64	2.14	21.00	22.96	5.69	13.98	11.2	0.338	0.371	0.75	17	86	103
69	6.97	28.96	22.05	47.41	12.55	15.70	22.3	-1.416	1.987	3.39	84	4	88
70	5.59	13.46	19.02	21.69	13.88	10.66	14.1	-0.452	0.404	0.97	25	43	68
71	4.05	20.68	21.37	19.94	12.46	23.86	17.1	-0.689	1.085	1.72	66	20	86
72	7.13	1.27	26.84	20.96	9.22	13.49	13.2	0.559	-0.310	1.13	34	56	90
80	1.51	1.31	20.23	23.21	17.19	6.60	11.7	0.759	0.284	1.5	54	80	134
86	6.63	2.23	18.05	22.80	12.34	8.12	11.7	0.299	-0.255	0.63	13	78	91
87	1.79	0.62	22.88	13.14	20.28	20.65	13.2	1.314	0.141	2.55	76	55	131
88	8.64	2.80	30.27	21.26	26.18	13.48	17.1	1.308	-0.287	2.55	77	19	96
90	8.95	0.85	22.43	18.18	11.34	22.22	14.0	0.750	-0.475	1.53	57	45	102
91	2.39	0.76	24.41	20.38	14.57	13.69	12.7	0.923	0.189	1.8	68	58	126
92	2.00	0.69	20.64	27.60	12.33	9.24	12.1	0.678	0.379	1.37	49	72	121
93	4.00	5.99	21.53	23.62	17.17	12.96	14.2	0.510	0.363	1.05	30	41	71
94	1.72	8.05	17.02	25.25	14.44	22.46	14.8	0.417	0.908	1.22	40	37	77
97	1.62	0.99	18.31	20.94	19.03	26.36	14.5	1.330	0.534	2.64	78	40	118
98	22.86	21.61	24.96	20.30	10.68	8.98	18.2	-1.632	-1.180	3.38	83	9	92
99	15.96	23.35	24.39	22.29	10.83	7.40	17.4	-1.634	-0.302	3.18	81	16	97
102	4.61	20.99	19.82	19.06	14.59	16.45	15.9	-0.868	0.880	1.9	72	29	101
104	9.50	12.16	15.17	20.83	10.59	15.33	13.9	-0.582	-0.063	1.13	35	46	81
105	1.25	0.52	20.21	16.07	14.81	10.93	10.6	0.769	0.086	1.5	53	91	144
114	1.89	1.85	25.11	18.08	11.06	15.42	12.2	0.718	0.227	1.41	50	69	119
115	2.01	29.08	17.38	23.04	12.64	14.41	16.4	-1.651	1.629	3.59	85	23	108
124	7.23	1.52	17.61	18.33	10.67	14.41	11.6	0.380	-0.410	0.84	21	82	103
127	10.27	7.69	24.02	40.32	8.30	22.30	18.8	0.227	0.479	0.65	14	7	21

128	4.59	29.73	24.92	15.44	9.67	12.82	16.2	-1.833	1.089	3.72	86	26	112
143	21.99	5.71	15.92	21.91	9.32	16.33	15.2	-0.369	-1.680	1.83	69	32	101
144	4.17	0.78	22.34	15.23	12.20	11.97	11.1	0.620	-0.239	1.23	41	88	129
146	7.81	31.66	24.38	24.65	6.77	13.51	18.1	-2.135	1.149	4.3	87	10	97
CS	6.76	12.38	26.03	17.72	10.58	16.64	15.0	-0.253	0.197	0.53	8	34	42
SQ1	11.25	10.27	22.54	21.60	12.01	13.95	15.3	-0.247	-0.308	0.57	10	31	41
Mean	8.05	8.72	22.11	22.14	12.64	14.41	14.7						
Variance	70.74	110.9	20.60	43.60	18.76	24.0	80.4						
IPCAe1	-2.235	-7.343	2.192	0.740	4.304	2.341							
IPCAe2	-6.930	2.969	0.221	2.255	0.348	1.136							

IPCAg1, IPCAg2, principal components of interaction for genotypes; IPCAe1, IPCAe2, principal components of interaction for environments.

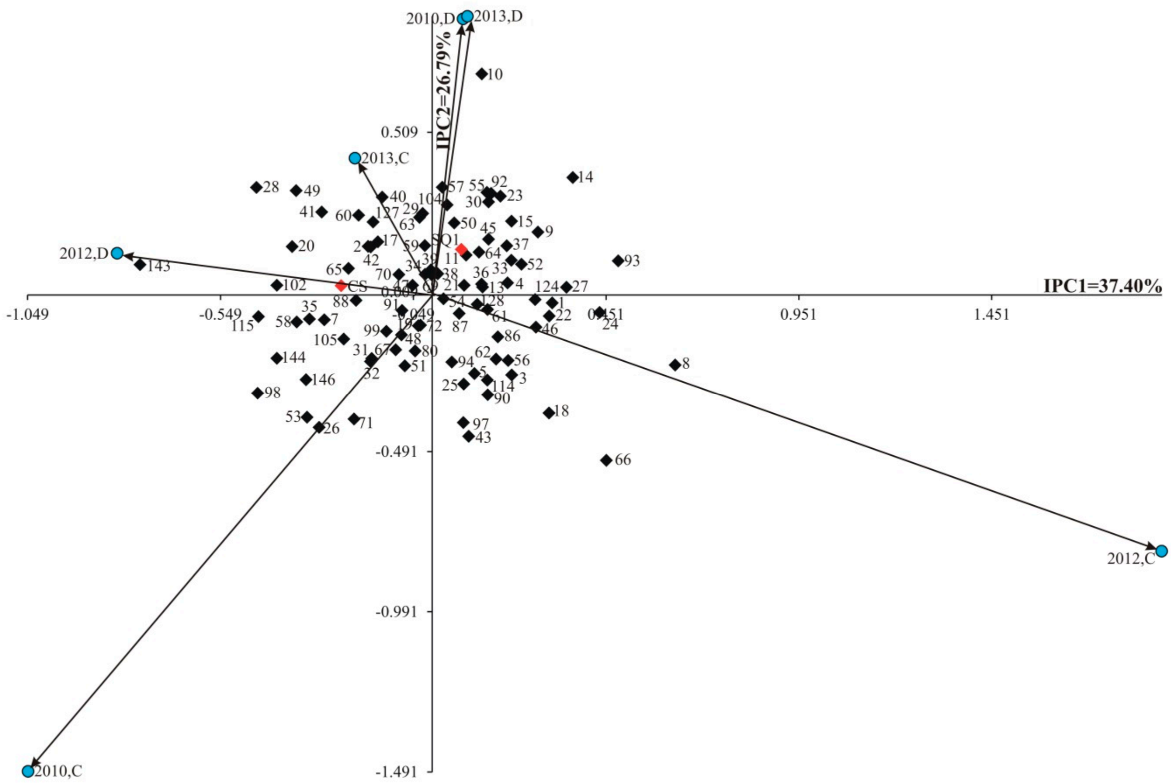

Figure S1. Biplot for genotype by environment interaction of yield in doubled haploid lines of wheat and their parental components in six environments, showing the effects of primary and secondary components (IPCA 1 and IPCA 2, respectively)

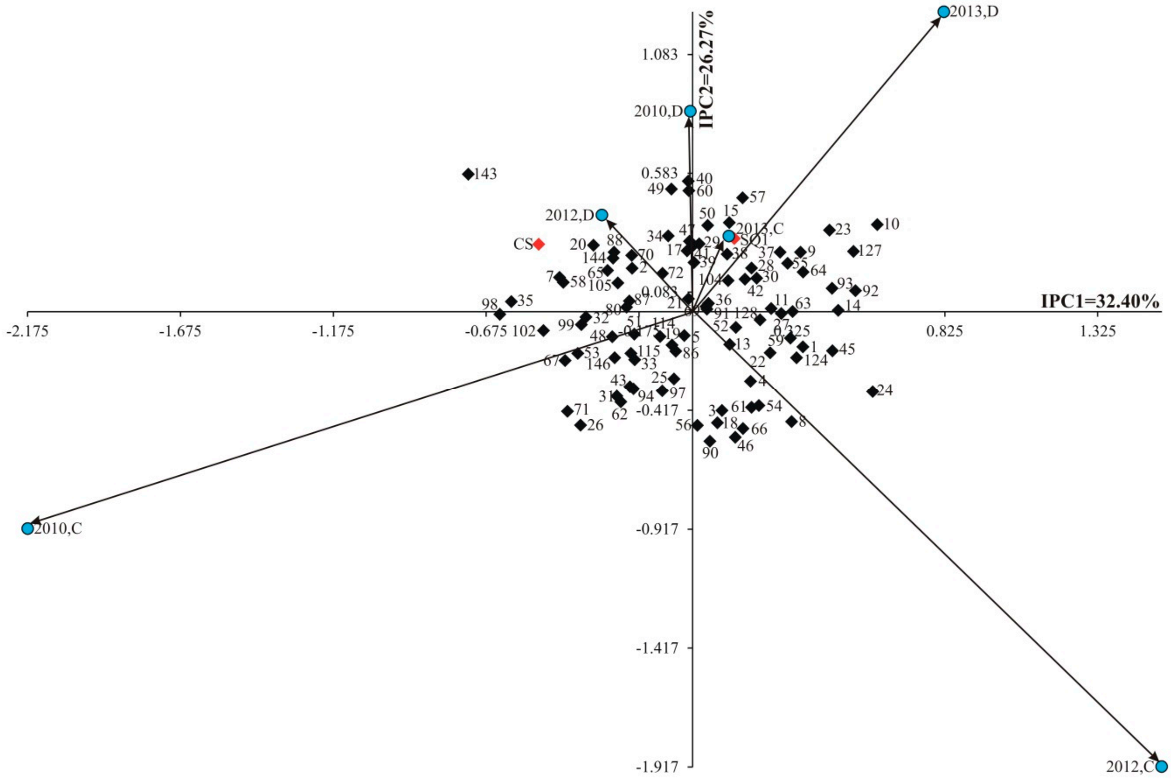

Figure S2. Biplot for genotype by environment interaction of biomass in doubled haploid lines of wheat and their parental components in six environments, showing the effects of primary and secondary components (IPCA 1 and IPCA 2, respectively)

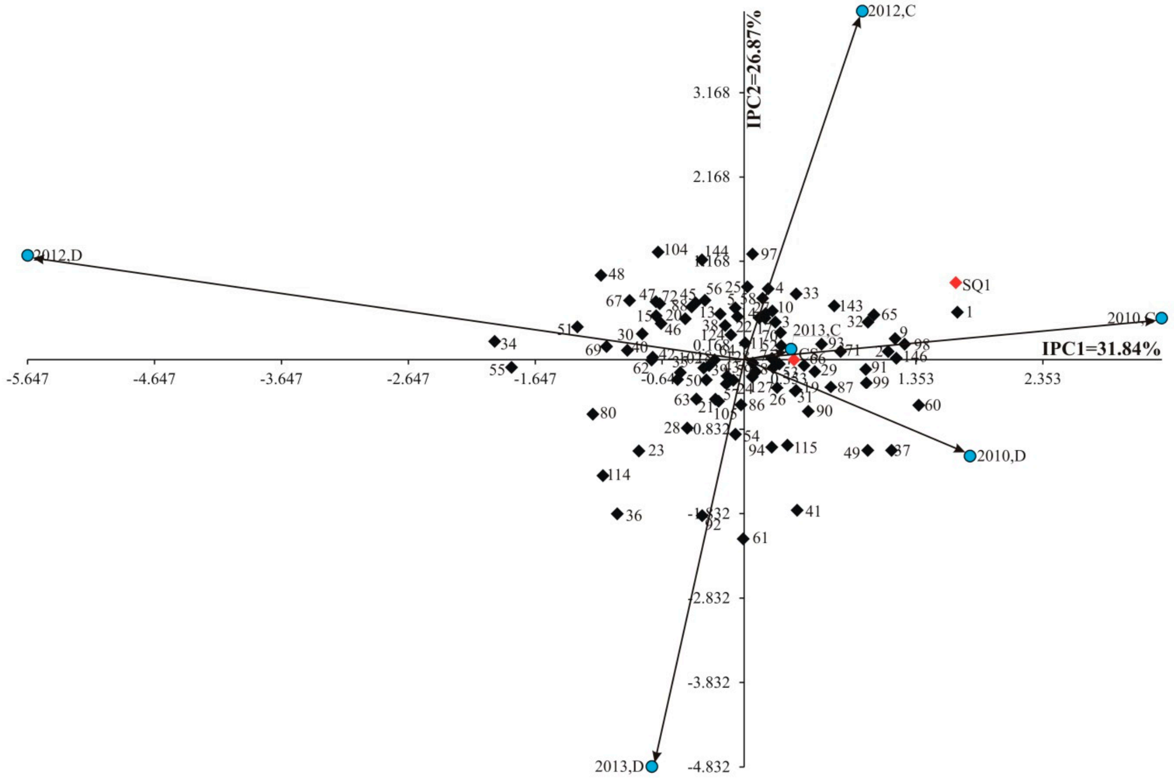

Figure S3. Biplot for genotype by environment interaction of TGW in doubled haploid lines of wheat and their parental components in six environments, showing the effects of primary and secondary components (IPCA 1 and IPCA 2, respectively)

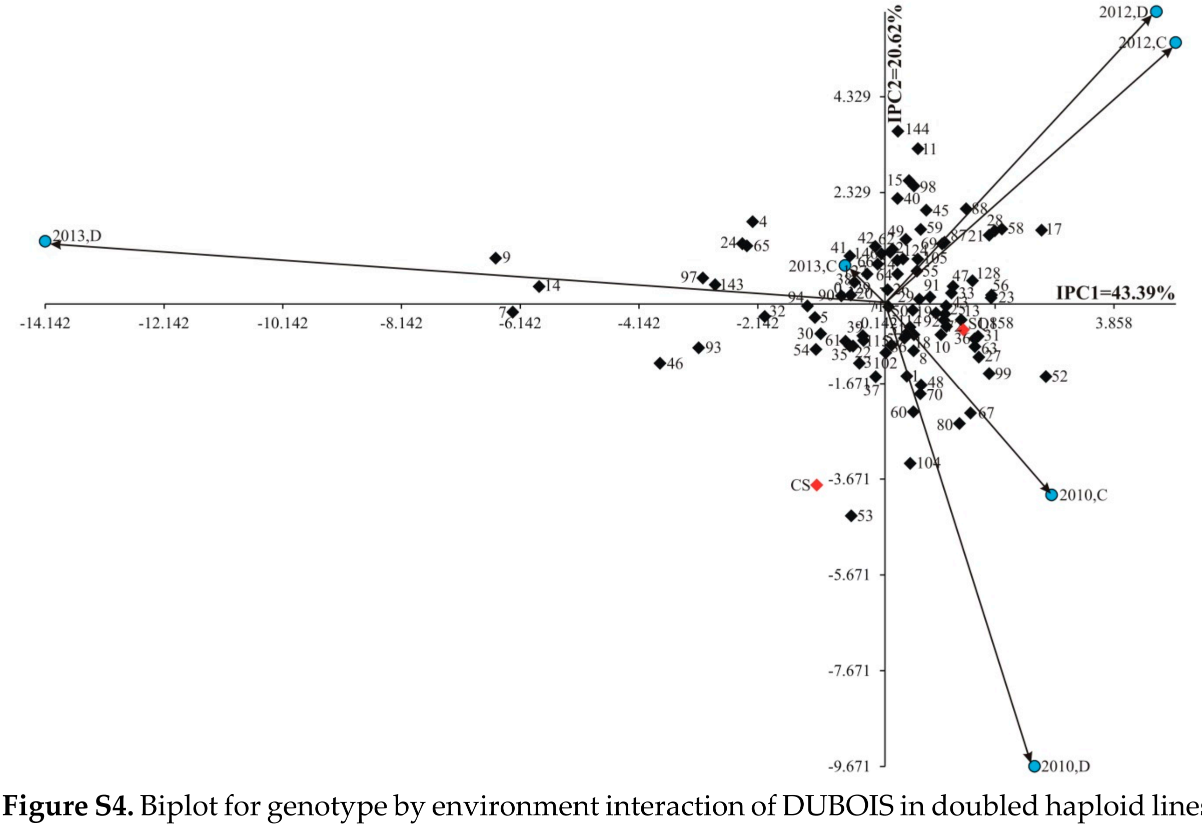

Figure S4. Biplot for genotype by environment interaction of DUBOIS in doubled haploid lines of wheat and their parental components in six environments, showing the effects of primary and secondary components (IPCA 1 and IPCA 2, respectively)

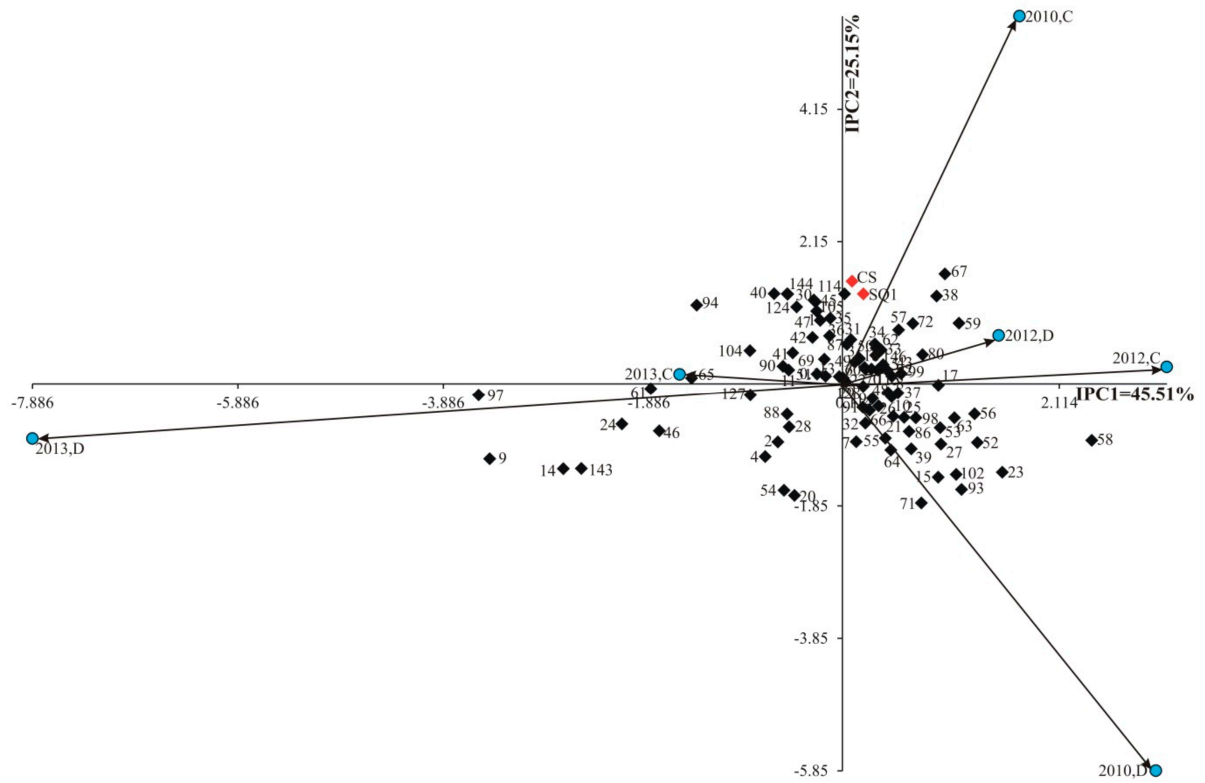

Figure S5. Biplot for genotype by environment interaction of glucose in doubled haploid lines of wheat and their parental components in six environments, showing the effects of primary and secondary components (IPCA 1 and IPCA 2, respectively)

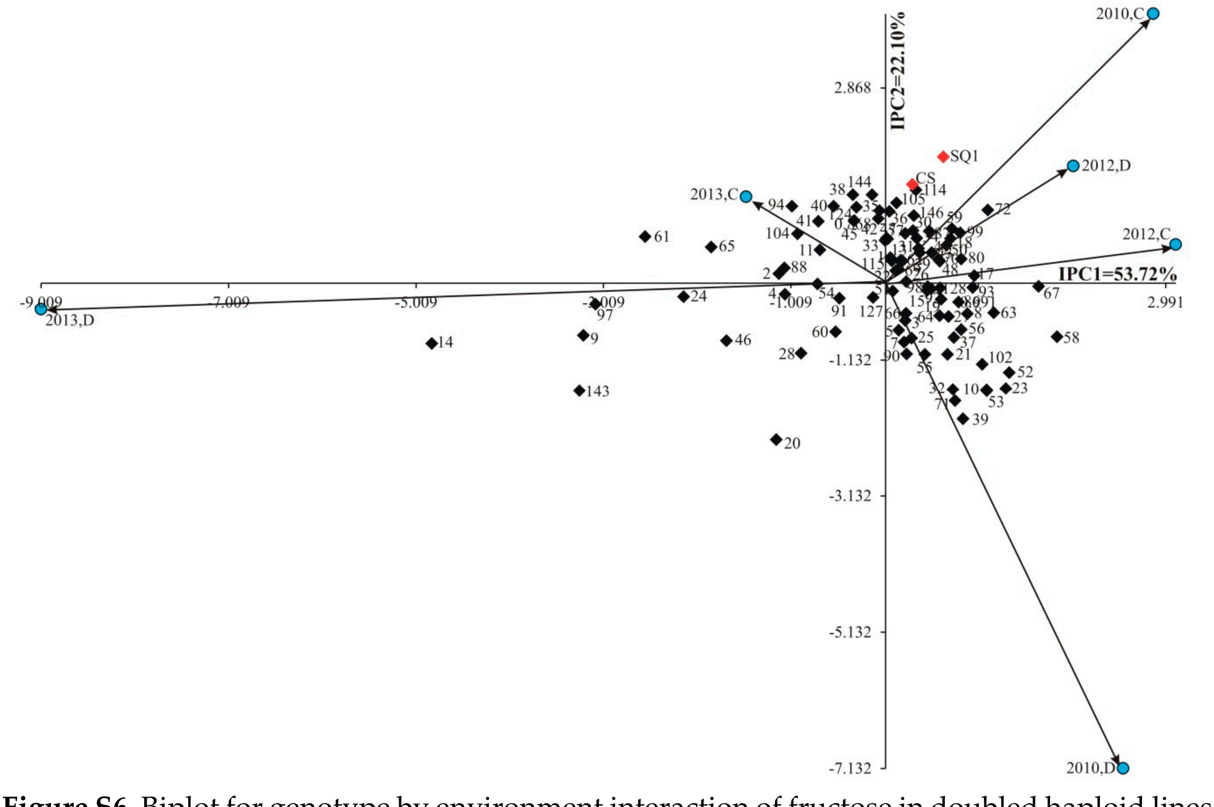

Figure S6. Biplot for genotype by environment interaction of fructose in doubled haploid lines of wheat and their parental components in six environments, showing the effects of primary and secondary components (IPCA 1 and IPCA 2, respectively)

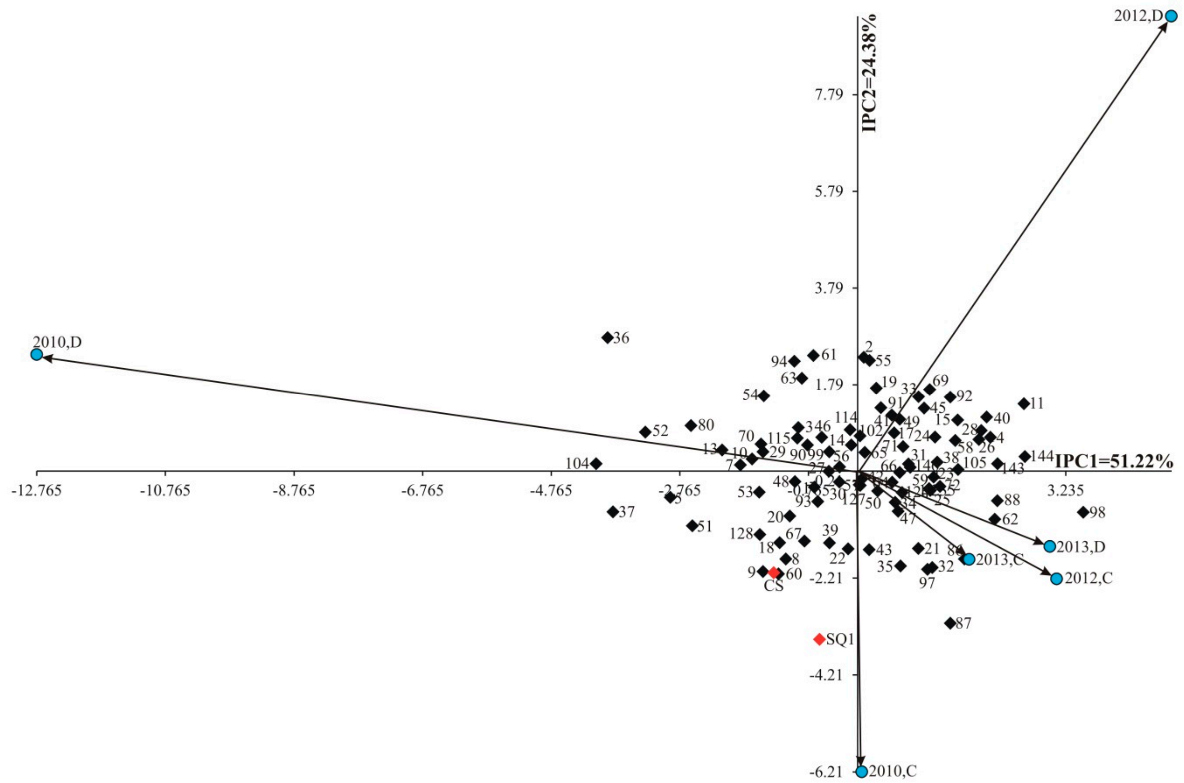

Figure S7. Biplot for genotype by environment interaction of sucrose in doubled haploid lines of wheat and their parental components in six environments, showing the effects of primary and secondary components (IPCA 1 and IPCA 2, respectively)

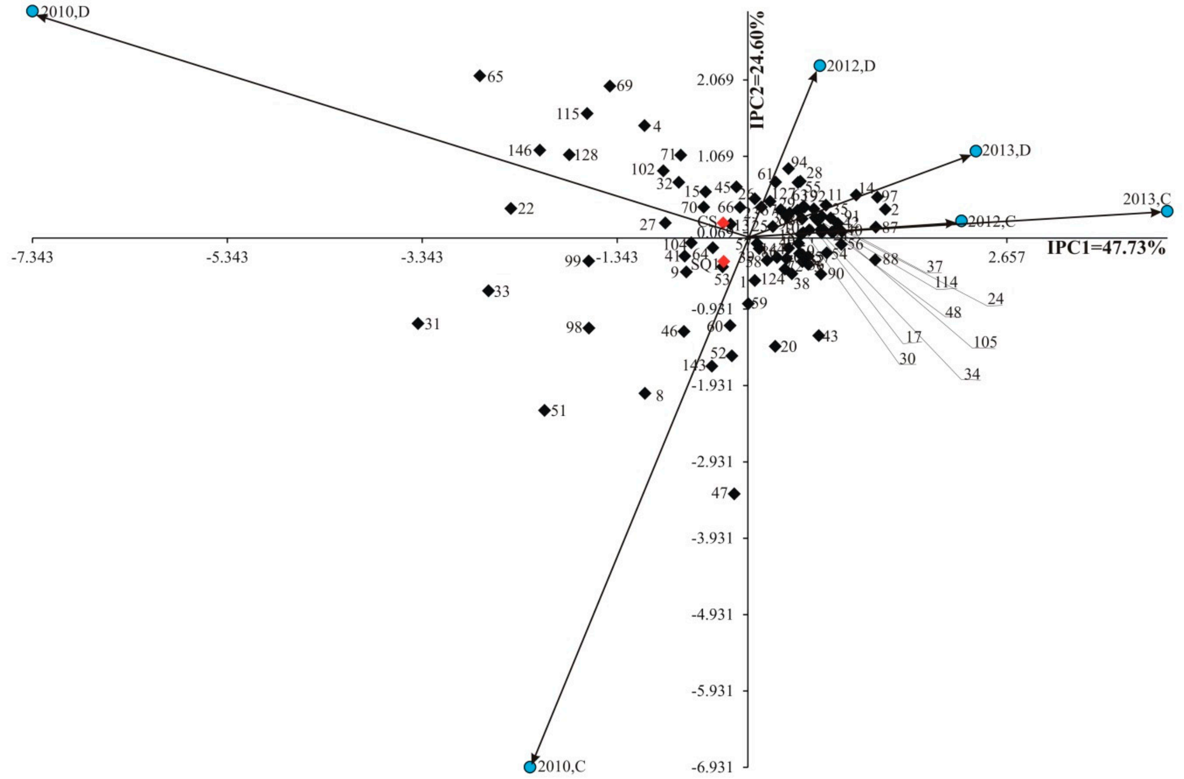

Figure S8. Biplot for genotype by environment interaction of maltose in doubled haploid lines of wheat and their parental components in six environments, showing the effects of primary and secondary components (IPCA 1 and IPCA 2, respectively)
